# ssGSEA score-based Ras dependency indexes derived from gene expression data reveal potential Ras addiction mechanisms with possible clinical implications

**DOI:** 10.1038/s41598-020-66986-8

**Published:** 2020-06-24

**Authors:** Ming Yi, Dwight V. Nissley, Frank McCormick, Robert M. Stephens

**Affiliations:** 1 NCI RAS Initiative, Cancer Research Technology Program, Frederick National Laboratory for Cancer Research, Frederick, MD USA; 2 UCSF Helen Diller Family Comprehensive Cancer Center, San Francisco, CA USA

**Keywords:** Cancer genomics, Data mining

## Abstract

For nearly a decade, the difficulties associated with both the determination and reproducibility of Ras-dependency indexes (RDIs) have limited their application and further delineation of the biology underlying Ras dependency. In this report, we describe the application of a computational single sample gene set enrichment analysis (ssGSEA) method to derive RDIs with gene expression data. The computationally derived RDIs across the Cancer Cell Line Encyclopedia (CCLE) cell lines show excellent agreement with the experimentally derived values and high correlation with a previous in-house siRNA effector node (siREN) study and external studies. Using EMT signature-derived RDIs and data from cell lines representing the extremes in RAS dependency, we identified enriched pathways distinguishing these classes, including the Fas signaling pathway and a putative Ras-independent pathway first identified in NK cells. Importantly, extension of the method to patient samples from The Cancer Genome Atlas (TCGA) showed the same consensus differential expression patterns for these two pathways across multiple tissue types. Last, the computational RDIs displayed a significant association with TCGA cancer patients’ survival outcomes. Together, these lines of evidence confirm that our computationally derived RDIs faithfully represent a measure of Ras dependency in both cancer cell lines and patient samples. The application of such computational RDIs can provide insights into Ras biology and potential clinical applications.

## Introduction

The Ras genes (KRAS, NRAS, HRAS) are the most frequently mutated oncogenes in human cancers, with KRAS showing the highest overall mutation frequencies^1,2^. Although KRAS mutations mainly occur in pancreatic, lung, and colon cancers, Ras gene mutations and amplifications are also found in many other cancer types. Given the significant role of these mutations, full-scale efforts to advance our understanding of Ras biology are underway with the hope of providing insights into oncogenesis mechanisms that will provide benefits for cancer diagnosis and treatment^2–5^.

Nearly a decade ago, efforts were made to investigate the oncogene “addiction” phenomenon, whereby tumors require the sustained expression and activity of a single aberrantly activated oncogene^6^. These efforts led to the classification of cancer cell lines into two categories: Ras dependent and Ras independent^7^. To assess variable KRAS dependency across a panel of KRAS mutant human cancer cell lines, Ras dependency indexes (RDIs) were proposed and measured experimentally to examine cancer cell addiction to oncogenic KRAS in a quantitative manner^7^. Many molecular features that distinguish KRAS-dependent and KRAS-independent cancer cell lines have been uncovered. For example, PI-3 kinase activation was found to contribute to the loss of KRAS dependency in a context-specific manner^7^. Similarly, KRAS dependency was found to be strongly linked to epithelial differentiation status^7^. In other words, the EMT (epithelial to mesenchymal transition) or MET (mesenchymal to epithelial transition) status is associated with Ras dependency. As a result, an EMT signature that was linked to KRAS addiction was identified, and this signature has value for the prediction of KRAS dependency by classification using the PAM (prediction analysis for microarrays) method applied to gene expression data^7^.

Interestingly, a subsequent effort was made with a colon-specific study that derived a colon-specific gene signature^8^. Inspired by these studies, recent efforts have revealed that all KRAS-deficient cells exhibit phosphoinositide 3-kinase (PI3K)-dependent mitogen-activated protein kinase (MAPK) signaling

and induced sensitivity to PI3K inhibitors^9^. This topic was further discussed in the Ras Central Dialogue^10^.

The concept of RDIs has provided new insights into oncogene addition phenomena. However, the experimental procedure used to determine RDIs is both time and labor consuming and is not easily reproduced. This likely stems from the diversity in the culture conditions of a variety of cancer cell lines (in-house observations) and, potentially, the biological readout assessed (apoptosis, survival or proliferation). Thus, there are only a handful of cancer lines for which RDIs exist that are available for researchers to utilize. This obviously represents a hurdle to the application of RDIs to wider libraries of existing cancer lines and consequently has slowed progress in understanding the mechanisms of Ras dependency and their relevance across different cancer samples.

Derivation of RDIs for the full panel of available cancer cell lines from CCLE collections (and other sources) would greatly facilitate the study of Ras dependency and stimulate research for understanding the underlying biological mechanisms. We postulate that although the current experimentally derived list of RDIs is relatively small, using the experimental values that have been determined as a metric to validate computationally derived estimates would permit extension across the full breadth of the CCLE collection of cell lines. Such a computational process would avoid many of the experimental obstacles outlined above. Further, this would allow an extension to those cell lines derived from cancers presumed to be unrelated to RAS dependency and possibly even application to patient samples.

Based on this rationale and given the expression data and existing gene signatures, an existing method, namely, the ssGSEA method, seems a perfect fit for our need^11^. The ssGSEA method is an extension of the GSEA method^12,13^, working at the level of a single sample rather than a sample population as in the original GSEA application. The score derived from ssGSEA reflects the degree to which the input gene signature is coordinately up- or downregulated within a sample^11^.

In this report, we used previously reported Ras-dependency-related gene signatures and expression data to computationally derive ssGSEA score-based RDIs for the full panel of cell lines from CCLE. Surprisingly, the derived ssGSEA RDI scores, especially those derived using the EMT signature^7^, show excellent correlation with experimentally measured RDIs independent of the technology platform (RNAseq and microarray). Interestingly, these ssGSEA RDI scores also showed a close relationship with our previous in-house siREN study that examined oncogene dependency^14^ and high-throughput RNAi (RNA interference) and CRISPR (clustered regularly interspaced short palindromic repeats) screening studies from the Broad Institute that defined a cancer dependency map and identified cancer-type-specific vulnerabilities on genes in the entire genome, respectively^15,16^.

Using these computed RDIs, we applied our comprehensive pathway pattern extraction pipeline (PPEP) analysis^17^ and gene-level pathway analysis to the expression profiles of cell lines with high and low derived ssGSEA scores. This showed that the main differences between the most highly Ras-dependent and Ras-independent cell lines were related to differences in the Fas signaling pathway and an NK cell activation pathway that leads to MAPK activation independent of Ras. The latter pathway, originally identified in NK cells, is functionally relevant and operates in a RAS-independent manner in a generic cancer context, thus extending its importance beyond the immune cell functional domain. Additionally, using expression data to derive EMT signature-based ssGSEA scores for individual TCGA patient tumor samples, the same two pathways were identified, extending the applicability of RDIs to patient samples. Finally, the ssGSEA scores of RAS dependency-related signature and Ras pathway genes showed a significant association with cancer patients’ survival outcome by our in-house survival analysis method^18^. Together, these observations indicate that computationally derived ssGSEA scores faithfully represent the levels of Ras dependency of cancer cell lines and cancer patient samples, directly providing insights for cancer research and potential clinical applications. To our knowledge, this is the first report of a computational method that uses genome-wide gene expression profiling to represent RDIs, and we assert that these findings constitute an opportunity to revitalize the RAS dependency discussion.

## Methods

### Data preparation

Gene expression data, including microarray and RNAseq data for CCLE, were downloaded from the CCLE portal (https://portals.broadinstitute.org/ccle). RNAseq data, either processed by CCLE or raw data, were downloaded and processed in-house with a customized procedure for the purpose of comparison. The RNAseq data from TCGA for selected tumor types were downloaded from the TCGA data portal (https://portal.gdc.cancer.gov/). Gene signatures were obtained from various sources. In summary, a list of many gene signatures was used to derive ssGSEA scores, including EMT_RasDep_Sig: Ras-dependent EMT signatures^7^; EMT_RasDep_Sig_Valid: Ras-dependent EMT signatures of only validated genes^7^; Colon_RasDep_Sig: colon signatures^8^; BMC_RasDep_Sig: Ras pathway dependency signatures^19^; RAS_pathway_v3: Ras pathway gene signature updated and derived from the Ras pathway from Ras Central (https://www.cancer.gov/research/key-initiatives/ras/ras-central/blog/2015/ras-pathway-v2). siREN_DEGs_KRAS_vs_RSK_Full and siREN_KRAS_vs_RSK_sig are differentially expressed genes (DEGs) between KRAS- and RSK-type lines and EN-derived gene signatures, respectively, from a previous siREN study (Table S4 in Yuan *et al*.^14^). “Up” and “Dn” in the colon signature indicate upregulated or downregulated signature subsets, respectively, as defined in the original publication^8^. The Ras pathway dependency signature was obtained from the original publication^19^. The KEGG (Kyoto Encyclopedia of Genes and Genomes) pathway signatures used KEGG pathway gene lists (http://www.genome.jp/kegg/pathway.html). The Biocarta pathway signature used the Biocarta pathway gene lists from MSigDB of the Broad Institute, and all Biocarta pathway maps used in the manuscript with data overlay were manually drawn based on the pathway information from MSigDB of UC San Diego and the Broad Institute.

(http://software.broadinstitute.org/gsea/msigdb/genesets.jsp?collection=CP:BIOCARTA) (note: the original Biocarta pathway collection database has been retired), which is licensed under CC BY 4.0 (https://creativecommons.org/licenses/by/4.0/) as described (https://www.gsea-msigdb.org/gsea/msigdb_license_terms.jsp).

MEK or RAF inhibitor IC50 data were downloaded from the GDSC database^20^. The siREN EGFP channel AUC data for chosen nodes were in-house data^14^. KRAS microarray data, copy number data, and many metadata for CCLE lines, including KRAS gene mutation status, tissue type, and zygosity, were all downloaded from the CCLE portal (https://portals.broadinstitute.org/ccle) and used as controls for the purpose of comparison of their behaviors with the derived ssGSEA scores, e.g., correlations with experimentally measured RDIs from the original study^7^ as well as correlation with the EMT signature. DEMETER scores and CERES scores were obtained from the Cancer Dependency Portal (https://portals.broadinstitute.org/achilles) based on initial publications^15,16^.

### Data analysis

Data manipulation and analysis were mostly performed using customized R scripts or existing R packages (www.r-project.org). The R code for the ssGSEA analysis method was obtained through email communications with the original authors of ssGSEA^11^ and customized to fit the needs for computing ssGSEA scores for each CCLE cell line or TCGA patient sample for the selected EMT signature^7^, colon signature^8^, BMC Ras dependency signature^19^, MSigDB gene sets or genes from individual Biocarta or KEGG pathways using microarray or RNAseq data. The two original experimentally measured RDI datasets were taken from supplementary figure 1B (referred to as RDI_SF1B) and supplementary figure 7 (referred to as RDI_SF7) from the original publication by Settleman^7^. RDI_SF7 predicted Ras dependencies based on the PAM algorithm but with subsequent experimental validation^7^. For comparison with ssGSEA scores, the RDI_SF7 data were first transformed by base 10 logarithm and then multiplied by (−1) for RAS-dependent lines or by (1) for RAS-independent lines to reflect the direction of Ras dependency (positive for Ras dependency and negative for Ras independency). The data were then standardized to z-scores across these cell lines for comparison. ssGSEA scores or the RDI data were also converted to z-scores for the purpose of comparison in heatmaps and the creation of correlation matrix heatmaps and correlation statistics.

DEGs for microarray data were derived using the limma^21^ R package, and DEGs for RNAseq data were derived using three popular analysis methods: the DESeq2^22^, edgeR^23^, and limma-voom^24^ R packages (www.r-project.org) to evaluate robustness/consistency. The top 15 vs. bottom 15 samples or top 30 vs. bottom 30 samples were also used for evaluating robustness/consistency. The corresponding DEG lists were collected and applied to customized R scripts that implemented the in-house developed pathway pattern analysis method called PPEP, which was previously described^17^. Briefly, the gene lists were subjected to Fisher’s exact test-based pathway or gene set enrichment analysis using annotated databases including Gene Ontology (www.geneontology.org/), KEGG pathways (www.genome.jp/kegg/pathway.html) and MSigDB (http://software.broadinstitute.org/gsea/msigdb/index.jsp). The derived p-values were transformed by using the formula (-1)*log10(p-value) with all p-values less than 0.05, and p-values with the number of “hit” genes from the gene list for the corresponding pathway/gene set less than 2 were all converted to 0. The transformed p-value data matrix was used to derive pathway-level heatmaps using either customized R scripts that used the pheatmap or gplots R package or the TM4 MeV tool from TIGR (mev.tm4.org/). The “hit” genes for specific pathways or gene sets from selected DEG lists were retrieved using PPEP-based customized R scripts, and gene-level heatmaps were generated similarly to the pathway-level heatmaps. PCA (principal component analysis) plots were generated using the Partek genomic suite (www.partek.com). The significance of the overlaps between the gene lists and gene signatures was assessed using Fisher’s exact test with customized R scripts. Survival analysis for association of ssGSEA scores of Ras-dependency related signatures or Ras pathway genes with cancer patients’ survival outcome from TCGA was performed using an in-house analysis method described previously^18^. GeneGO database query and network analysis were performed using the GeneGO portal (https://portal.genego.com/cgi/data_manager.cgi#).

## Results

### ssGSEA scores derived using the EMT signature are highly correlated with experimentally measured RDIs regardless of the data source and technology platform

To expand the cell line coverage of experimentally derived Ras dependency indexes, we developed a robust computationally derived metric that could be calculated from gene expression data that was able to faithfully reproduce the experimentally derived values. As a proof of concept, we used this method to compute the ssGSEA scores for each CCLE cell line using gene expression data.

**Figure 1:**
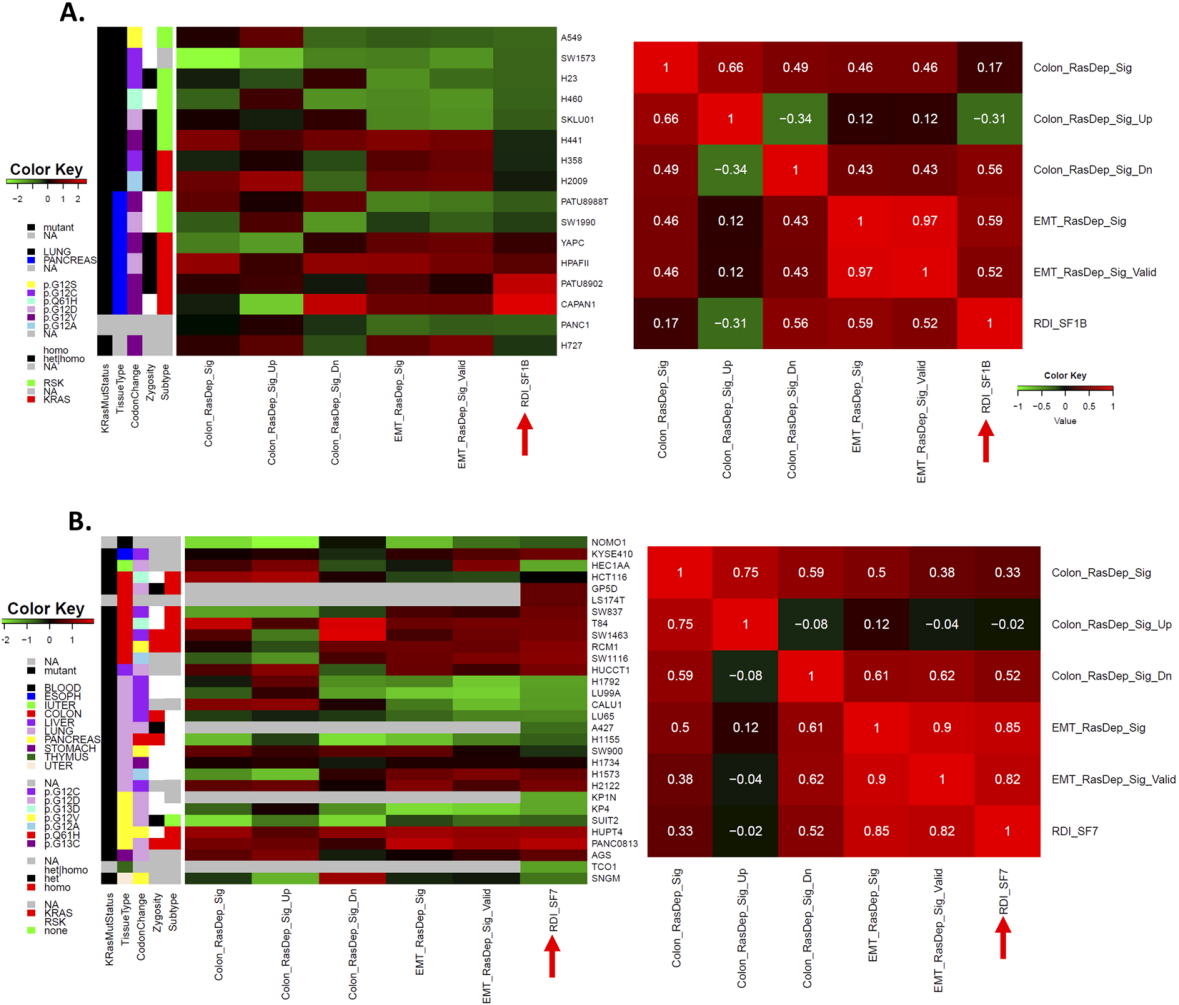
A comparison of derived ssGSEA scores and Ras dependency indexes (RDIs) showed strong and consistent correlation patterns. (**A**) Left panel: Derived ssGSEA scores and experimentally measured Ras dependency indexes (RDI_SF1B, red arrow, also see method section) showed consistent patterns with very few exceptions in the heatmap. Right panel: Heatmap of the correlation matrix for all data from the left panel. RDI_SF1B is indicated by a red arrow. (**B**) Left panel: Derived ssGSEA scores and predicted Ras dependency indexes (RDI_SF7, red arrow, also see the Method section) showed consistent patterns with very few exceptions in the heatmap. ssGSEA scores for each cell line with available microarray data were computationally derived using EMT signatures (Singh *et al*. 2009) and colon signatures (Singh *et al*. 2012) and standardized into z-scores across cell lines. Right panel: Heatmap of the correlation matrix for all data from the left panel. RDI_SF7 is indicated by a red arrow. The gray color in all heatmaps indicates missing data. EMT_RasDep_Sig: EMT signatures; EMT_RasDep_Sig_Valid: EMT signatures of only validated genes; Colon_RasDep_Sig: colon Signature. “Up” or “Dn” are upregulated or downregulated signatures, respectively, as defined in the original publication. KRAS gene mutation status (KRasMutStatus), tissue types, actual KRAS mutations, heterozygosity, and classification of RSK and KRAS by siren data for these cell lines are also displayed (also see the Methods section).

There are two sets of RDI data derived from the original study that were validated experimentally^7^, each of which covers different cell lines. We named these sets RDI_SF1B and RDI_SF7, since they are from supplementary figure 1B and supplementary figure 7 of the original paper, respectively^7^. We first used both of these sets of data for comparison with ssGSEA-computed scores from the corresponding cell lines using the CCLE microarray data. Both sets of data showed a very consistent pattern with the computationally derived data in heatmaps of actual data (left panels of Fig. 1A,B) and their derived correlation matrices (right panels of Fig. 1A,B) between the original RDI values and the derived ssGSEA scores using the EMT gene signature that was derived in the initial report^7^. Among others, RDI_SF7 shows a better correlation (Fig. 1B) than RDI_SF1B (Fig. 1A). The same observation was obvious both from the plots of the actual data and from the statistical metrics of correlation, which showed great statistical significance (Supplementary Figures 1A,B). Interestingly, the consistency in the patterns seems to be independent of the KRAS mutation status/type and tissue type (Fig. 1A,B).

As still further support, the validated 5-gene signature subset from the reported EMT signature showed a very good correlation that was only slightly worse than the full EMT signature (Figs. 1A,B, Supplementary Figures 1C,D), suggesting a potentially critical role of these five genes in Ras dependency, as predicted. In contrast, the ssGSEA scores derived using the reported colon signatures^8^ do have some level of consistency and correlation with both sets of RDI data, but to a lesser extent (Fig. 1, Supplementary Figure 1).

**Figure 2:**
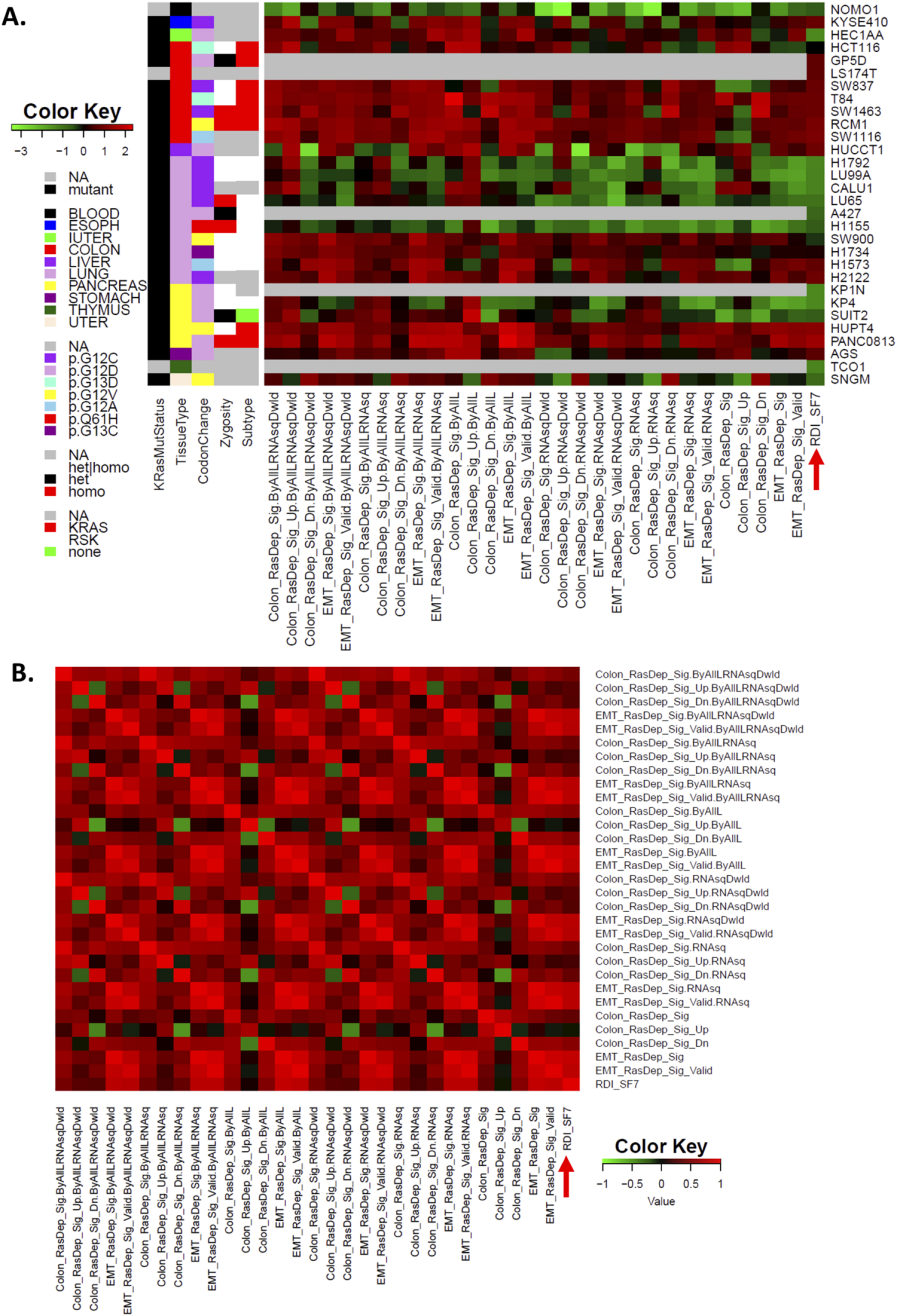
Comparison of derived ssGSEA scores and Ras dependency indexes (RDIs) showed overall consistent and correlation patterns regardless of data resources and technology platforms. (**A**) Derived ssGSEA scores and RDI_SF7 (red arrow, also see the Methods section) showed overall consistent patterns in the heatmap regardless of data resources and technology platforms. ssGSEA scores for each cell line were computationally derived using EMT signatures and the colon Signature and standardized into z-scores across cell lines as in Settleman’s publication or all of the CCLE lines (ByAllL) using either microarray or RNAseq data. RNAseq data was directly downloaded (RNAseqDwld) or processed by us (RNAseq). (**B**) Heatmap of the correlation matrix for all data from **A**. RDI_SF7 is indicated by a red arrow.

To test the robustness of the observed correlations, we also used RNAseq data either processed by the CCLE group or processed in-house to derive the ssGSEA scores. The z-scores produced from the derived ssGSEA scores from all CCLE cell lines or just within the cell lines used in the original publication (Singh *et al*. 2009) were used for comparison. The consistent pattern and high correlation levels are very similar between the ssGSEA scores from the EMT signature and both sets of RDI data regardless of the data source and technology platform (Fig. 2, Supplementary Figure 2). These results demonstrate that the ssGSEA computational method does indeed faithfully reproduce the experimentally derived RDIs and warrants the extension of the metric beyond those limited cell lines.

### ssGSEA scores derived using the EMT signature are highly correlated with data from related studies

The EMT signature-derived ssGSEA scores showed a relatively weak correlation with our in-house-derived siREN data in its original form^14^, KRAS gene expression, and other data (Supplementary Figures 1C,D). However, surprisingly, the EMT signature-derived ssGSEA scores showed a high correlation with the ssGSEA scores derived from KRAS-RSK signature genes (Supplementary Figures 3A,B) from that study despite a relatively weak correlation with ssGSEA scores derived from other signatures, including the colon signature, the list of Ras pathway genes or related signatures (Supplementary Figure 3B). The in-house siREN study^14^ was designed to study Ras-related oncogene dependency at the signaling node level as it related to cancer cell survival and other parameters, which is closely related to our study. In addition, the KRAS and RSK classification of the cell lines defined by that study indeed coincided quite well with the Ras-dependent and Ras-independent classification by the corresponding ssGSEA scores of EMT signatures and the colon signature (Supplementary Figure 3C). Correlation analysis with relevant data from an external large study, i.e., DEMETER data^15^ of RNAi screening showed moderate level correlation but with highly statistical significance for the data of a total of 460 cell lines shared between this dataset and ours (Supplementary Figure 3D), although the CERES scores^16^ of CRISPR screening showed only modest correlation with marginal significance (Supplementary Figure 3E). The relatively weak correlation with CERES scores was probably due to the limited sample size (only 33 shared cell lines) in contrast to a much larger sample size for DEMETER data (460 shared cell lines), but the trend was still detectable (Supplementary Figure 3E). All these observations indicated a potential biological connection between our computational RDIs and related studies, further consolidating their potential biological implications.

Interestingly, a direct assessment of the overlapping genes between the gene signatures revealed that the EMT signature and the KRAS-RSK signature have the most statistically significant overlap of shared genes (Table 1), which may partially explain why their derived ssGSEA scores showed the highest correlations. This result is not simply due to the size of the gene lists, since the large gene list for Colon_RasDep_Sig had a small overlap with EMT_RasDep_Sig. These observations demonstrate a strong connection between our novel ssGSEA score-based Ras dependency index and a previous relevant study^14^, consolidating the differences in two major subtypes that may define Ras dependency.

**Table 1:** Enrichment analysis of the level of shared genes between the gene signature lists.

List Pairs	Colon_RasDep_Sig	EMT_RasDep_Sig	EMT_RasDep_Sig_Valid	BMC_RasDep_Sig	RAS_pathway_v3	siREN_DEGs_KRAS_vs_RSK_Full	siREN_KRAS_RSK_sig
Colon_RasDep_Sig	1405	0.3634	0.3858	0.7009	2.49E-05	0.1434	0.239
EMT_RasDep_Sig	34	342	5.74E-09	0.0009	0.0732	**1.78E-37**	0.0085
EMT_RasDep_Sig_Valid	1	5	5	1	1	0.0039	1
BMC_RasDep_Sig	11	10	0	133	0.0039	0.3069	0.0398
RAS_pathway_v3	41	9	0	7	227	0.1986	0.1013
siREN_DEGs_KRAS_vs_RSK_Full	117	**105**	3	12	21	1146	1.13E-26
siREN_KRAS_RSK_sig	5	4	0	2	2	29	36

The EMT signature and the derived differential gene signatures in the siREN study have the most significantly overlapping genes (highlighted in bold text). Enrichment p-values were derived from Fisher’s exact tests on the levels of overlap between each pair of gene signature lists in the table using genes from the CCLE microarray data with valid signals as the background total number of genes in the 2 × 2 contingency table. The diagonal line (left top to right bottom) has the actual number of genes for each gene signature in bold. The right upper part of the diagonal line shows the p-values of the enrichment level assessed by Fisher’s exact test; the lower left part of the diagonal line shows the number of genes shared by the corresponding two-gene signatures. Colon_RasDep_Sig: colon signature; EMT_RasDep_Sig: EMT signatures; EMT_RasDep_Sig_Valid: EMT signatures of only validated genes; BMC_RasDep_Sig: BMC Ras signature; RAS_pathway_v3. siREN_DEGs_KRAS_vs_RSK_Full and siREN_KRAS_vs_RSK_sig are differentially expressed genes between KRAS- and RSK-type lines and EN-derived gene signatures, respectively, from Table S4 in Yuan *et al*.^14^.

### EMT signature-derived ssGSEA scores display wide ranges across tissues, suggesting that Ras dependence/independence is a relevant parameter in diverse tissues

The concept of Ras dependency has conventionally been applied primarily in the context of tissues in which KRAS mutations are frequent, including pancreatic, lung and colon cancers. With the availability of broadly computed RDI scores across tumor-derived cell lines from a variety of tumor types and tissue sources, we wanted to evaluate the diversity in scores from different tissue lineages. Interestingly, the EMT signature-derived ssGSEA scores from both microarray and RNAseq data showed broad but similar data ranges within various tissues, including the three main tissue types (lung, pancreas, and colon) (Supplementary Figure 4A), in which KRAS is known to play a critical role in oncogenesis. As seen from the figure, the distributions showed similar ranges regardless of KRAS mutation status (Supplementary Figure 4A), suggesting that both wild-type and KRAS mutant lines can be associated with Ras dependency. Furthermore, these ranges extend to the available additional tissue types, including breast and, to a lesser extent, thyroid (Supplementary Figure 4A), kidney, hematopoietic, central nervous system, ovary, and skin (Supplementary Figure 4B) tissues. Compared to the cell lines for which experimentally measured RDIs were determined, the computationally derived RDIs of other lines in various tissue types showed similar data ranges (Supplementary Figure 4A). This implies that the computational RDIs may indeed be able to represent the range of experimentally measured RDIs. Furthermore, cell lines derived from other tissues also display a range of Ras dependency, indicative of a potentially global impact of Ras genes on the oncogenic potential of the cell, which is perhaps to be expected given the critical roles played by these genes.

The correlations between the EMT signature-derived ssGSEA scores and the experimentally determined RDI scores were the best and higher than those observed with the colon signature-derived ssGSEA scores at an intermediate level, especially for RDI_SF1B (Fig. 1A,B). Thus, we wanted to also map the colon signature-derived ssGSEA scores against the larger cell line panel in the context of different tissues. The results of these projections are shown in Supplementary Figure 4C-4D.

We were surprised to note that the distribution of both EMT and colon signature-derived ssGSEA scores was rather similar between the microarray and RNAseq data and was minimally impacted by the KRAS mutation status, even though the actual original values were very different (note scales in left vs. right panels, Supplementary Figure 4A-4D). However, the distribution of colon signature-derived ssGSEA scores spanned a smaller range for the cell lines (Union_of Lines_SF1B_SF7) that have been experimentally measured for RDIs^7^ compared to other CCLE cell lines. This is in contrast to the EMT signature-derived ssGSEA scores, where Union_of Lines_SF1B_SF7 covers almost the same data range as the other CCLE lines (Supplementary Figure 4A and 4C). Since the previous study^7^ used very typical Ras-dependent and Ras-independent lines as examples, their derived RDIs would be expected to span the full range of dependency indexes. Thus, it seems likely that the EMT signature-derived ssGSEA scores represent a more valid and reliable way to reflect Ras dependency across global tissue types than those derived from the colon signature. In addition, the colon signature was derived using colon-based data, so it is also less likely to be broadly applicable than the EMT signature that was derived using cell lines from multiple tissue types^7,8^. Furthermore, although to a lesser extent, the distribution of colon signature-derived ssGSEA scores is more dissimilar between the microarray and RNAseq data-derived values than that obtained using the EMT signatures (Supplementary Figure 4A,B vs. 4C,4D), especially considering the extreme cases at both ends of the Ras dependency spectrum. This is consistent with the high relative correlation of the EMT signature-based ssGSEA scores with the experimentally measured RDIs that we described previously (Fig. 1 and Supplementary Figure 1). These observations further confirm that the EMT signature-based ssGSEA scores are highly stable regardless of the technology platform and KRAS mutation status, which may be attributed to increased biological relevance.

### EMT signature-derived ssGSEA scores are the best computational RDIs compared to those derived from other gene signatures or gene sets

We wanted to further investigate whether the EMT signature-derived ssGSEA scores represented the best metric of Ras dependency. For this, we leveraged several additional sources of Ras pathway involvement-associated gene lists. One was obtained through data mining for coherent expression of RAS pathway-related genes across multiple datasets for the specific purpose of prediction of RAS pathway dependency^19^. We also assessed the ability of the Ras pathway genes themselves to serve as a gene list source. For this, a consensus view of the Ras pathway was established and curated through cancer community efforts (https://www.cancer.gov/research/key-initiatives/ras/ras-central/blog/2015/ras-pathway-v2), as well as the BioCarta and KEGG Ras pathway gene lists.

We computed the ssGSEA scores with these gene lists/signatures using the cell line data and then compared the scores with the EMT signature and colon-derived scores described above. The ssGSEA scores derived from the EMT signature showed the best correlation with the original RDI datasets regardless of the data source or technology platform (Supplementary Figures 5 and 6A–C). Combining both sets of original RDI data and their corresponding cell lines, the EMT signature-derived ssGSEA scores from these lines showed the best correlation with both RDI datasets regardless of data source or platform (Supplementary Figure 6D). The robustness of the performance, consistently showing the best correlation with the original RDI datasets, indicates that the EMT signature-derived ssGSEA scores were the best and most robust computational RDIs that we tested.

### Expression profiling of computationally defined Ras-dependent and independent CCLE cell lines reveals two Biocarta pathways relevant to Ras dependency

Using our computational ssGSEA RDI scores, we next wanted to assess any underlying biology associated with Ras dependency across the much larger panel of cell lines with dependency scores. Based on the correlation of the ssGSEA scores with the original RDI datasets^7^, the cell lines with high ssGSEA scores correspond to Ras-dependent lines, whereas those with low ssGSEA scores are likely Ras independent. To identify biological differences or themes associated with dependence/independence, we applied a theme discovery approach to the 15 most dependent/independent (highest and lowest scores) cell lines along the dependency continuum. Interestingly, high-level PCA of the gene expression data between the top 15 vs. the bottom 15 cell lines (highest or lowest EMT signature-derived ssGSEA scores) in the lung, pancreas, and colon showed very different expression profiles between these two groups of cell lines in all three tissue types (Supplementary Figure 7A). This is indicative of very distinct transcriptional profiles between the Ras-dependent and Ras-independent lines. However, the majority of the Ras-independent lines reside along the PC1 axis (the PC that explains the most variability) in the PCA plot, whereas the Ras-dependent lines align at the other side of the same axis regardless of tissue type (Supplementary Figure 7A), displaying some commonality in the expression profile differences between Ras-dependent lines and Ras-independent lines for each of the three issue types.

To determine whether the observed differences between the top 15 highest or lowest ssGSEA scores of the EMT signatures were directly related to the EMT signature, we examined the most critical 5 genes of the EMT signature, which are the key genes comprising the EMT validated signature^7^. Interestingly, all five genes showed a consistent and expected expression pattern (Supplementary Figure 7B–D) compared to the original study^7^.

**Figure 3:**
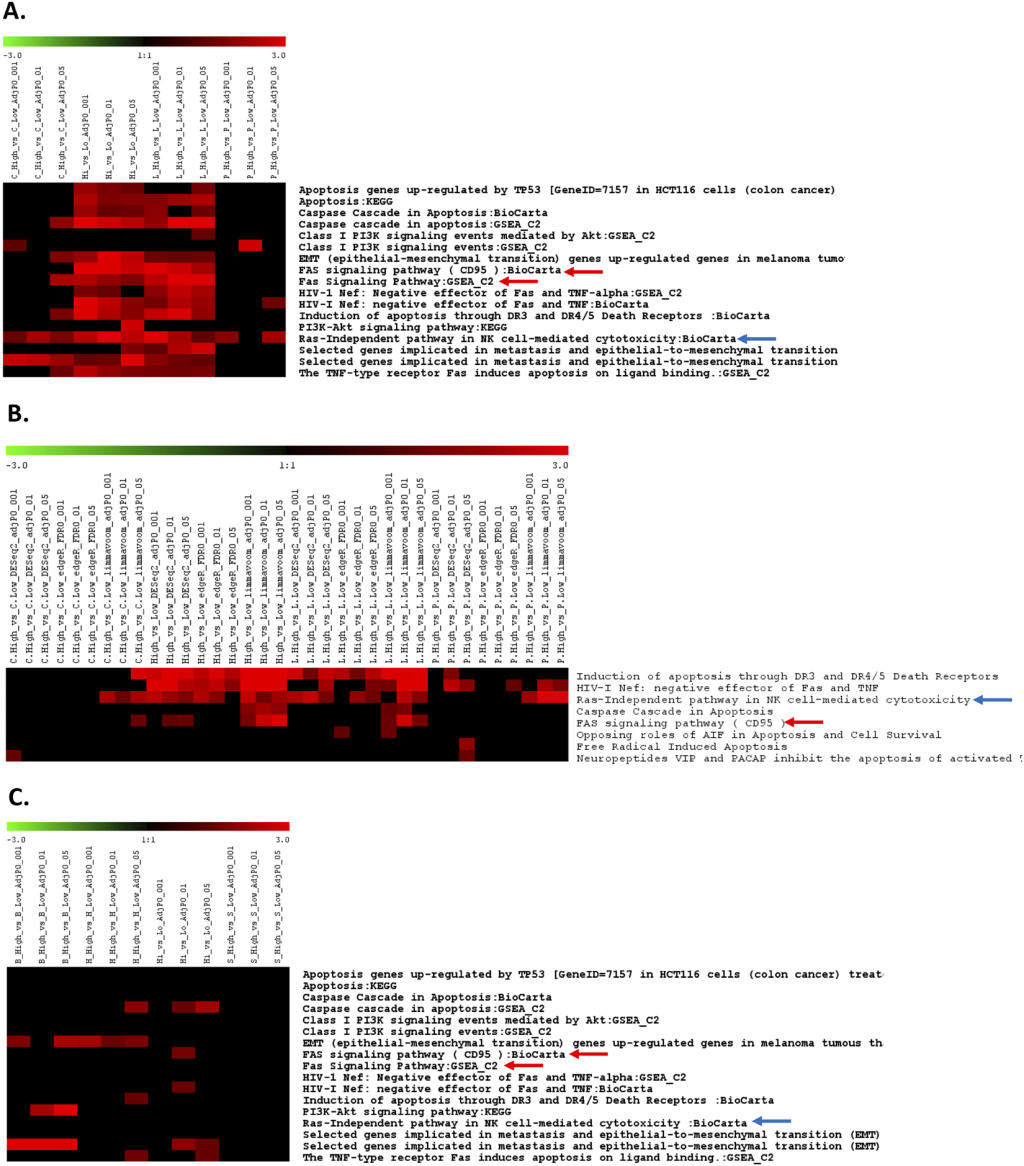
Analysis of differentially expressed genes between the top 15 vs. bottom 15 CCLE cell lines with the highest vs. lowest ssGSEA scores in three tissue types: lung, pancreas, and colon or large intestine. (**A**) Collective pathway enrichment pattern or PPEP analysis result of differentially expressed gene lists (detailed in Supplementary Table 1) of top 15 vs. bottom 15 CCLE cell lines with highest vs. lowest ssGSEA scores, respectively, in three tissue types, namely, lung (L), pancreas (P), and colon (C), and Hi_vs_Lo (pooled from three tissues) using microarray data. (**B**) Collective pathway enrichment pattern or PPEP analysis result of differentially expressed gene lists of top 15 vs. bottom 15 CCLE cell lines with highest vs. lowest ssGSEA scores, respectively, in three tissue types, namely, lung (L), pancreas (P), and colon (C), and Hi_vs Lo (pooled from all three tissues) using RNAseq data. (**C**) Collective pathway enrichment pattern or PPEP analysis result of differentially expressed gene lists of top 15 vs. bottom 15 CCLE cell lines with highest vs. lowest ssGSEA scores, respectively, in three tissue types, namely, hematopoietic and lymphoid tissue (H), breast (B), and skin (S), and Hi vs. Lo (pooled from three tissues) using microarray data.

To study the underlying biology associated with the difference between the Ras-dependent and Ras-independent cell lines defined by their corresponding computational RDIs, differentially expressed genes (DEGs) between the cell lines with the top and bottom ssGSEA scores were derived from expression data, including microarray and RNAseq data (Supplementary Tables 1 and 2), and subjected to in-house PPEP pathway analysis^7^ (Fig. 3A–C).

Based on scanning through the enriched terms identified by that analysis, the primary themes are the expected EMT-related terms, a Ras-independent pathway in NK cells, Fas signaling and apoptosis/caspase signaling, except for a relatively narrowly observed enrichment pattern for PI3k/AKT-related terms only in the pooled contrast DEG list and only with one cutoff. The Ras-independent pathway in NK cells and Fas signaling are the most commonly enriched among the DEGs. Based on their prevalent patterns and/or relevance to Ras dependency, we selected the RAS-independent NK pathway and FAS signaling pathway for further exploratory analysis. These pathways are intended to serve as prototypes for validation of the computational RDI scores rather than as parameters for exhaustive evaluation of Ras-dependence-associated biology, which will no doubt be revealed by further studies.

From both the microarray and RNAseq datasets, the Ras-independent NK pathway appears to show the broadest significance across the selected tissue types in which KRAS is thought to play a major role, including lung, colon, and pancreas; this is especially true for the microarray data (Fig. 3A) but is also true for the RNAseq data for at least one or more of the analysis methods applied, showing the highest prevalence in lung tissue (Fig. 3B). In contrast, this pathway did not show any enrichment patterns within other tissue types in which KRAS may be less involved (Fig. 3C), indicating a potential tissue-specific context component. This pathway, as indicated by its name, is relevant to Ras dependency and was originally found to be a pathway that signals the activation of downstream MAP kinase in a Ras-independent manner in NK cells^25,26^, so we could anticipate its identification if, indeed, the computational RDI scores represented a Ras-dependency metric.

The second pathway selection was inspired by the main theme of the observations from a recent report about the role of Fas in Ras dependency^27^; coincidentally, this pathway involves many of the apoptosis-related genes (caspases and death receptors). In contrast to the more universal NK pathway, the “FAS signaling pathway (CD95)” was specifically enriched in all of the DEGs from the lung in the microarray data and enriched in all of the limma-voom method-derived DEGs from the lung in the RNAseq data (Fig. 3A,B). This observation seemed indicative of lung-specific involvement of this pathway relevant to Ras dependency. As will be described later, this pathway may be more broadly applicable to Ras dependency (e.g., not limited to lung tissues) at the gene level than what was observed in the PPEP pathway enrichment pattern analysis shown here.

Surprisingly, the EMT signature has very few genes that overlap with these two Biocarta pathways, although two critical kinase genes, namely, SYK and PAK1, are shared between the EMT signature and Ras-independent pathway in NK cells, and PAK1 is one of the two genes shared between the EMT signature and FAS signaling pathway (Supplementary Table 3). We decided to examine whether there was any correlation between the ssGSEA scores derived from the EMT signature and these two pathways. Surprisingly, the pathway “Ras-independent pathway in NK cells” showed a very high level of correlation (correlation coefficients all above 0.8), with the best correlation (top 1 compared with other pathways) observed for their ssGSEA scores with those of the EMT signatures across CCLE cell lines from three different tissue types, namely, lung (Supplementary Figure 8A), pancreas (Supplementary Figure 8B) and colon (Supplementary Figure 8C), regardless of the technology platform (e.g., microarray or RNAseq data). This pathway was also among the top pathways with relatively good correlation (correlation coefficients between 0.37 and 0.47) between their ssGSEA scores and those of the EMT signature in pooled cell lines from these three tissue types (Supplementary Figure 8D). This suggested that the observed Ras-independent NK pathway in the PPEP analysis was observed not simply due to the same set of overlapping genes between this pathway and the EMT signature but rather due to the underlying biological connections indicated by the strong correlation in their expression profiles. In contrast, the FAS signaling pathway (CD95) showed enrichment with a relatively high correlation coefficient in lung cell lines (Supplementary Figure 8A) but not in the other two tissue types (Supplementary Figure 8B,C) and not in the pooled cell lines from all three tissue types (Supplementary Figure 8D). These observations suggest that these two pathways may indeed have strong biological relevance and connections with the concept of Ras dependency, with the Ras-independent pathway in NK cells having a wider and more global impact and appearing to be less tissue context sensitive than the Fas pathway (Fig. 3A,B).

### Comprehensive expression analysis of the canonical Ras-independent Biocarta pathway of NK cells uncovered consensus genes with unique expression patterns and critical roles in signaling

**Figure 4:**
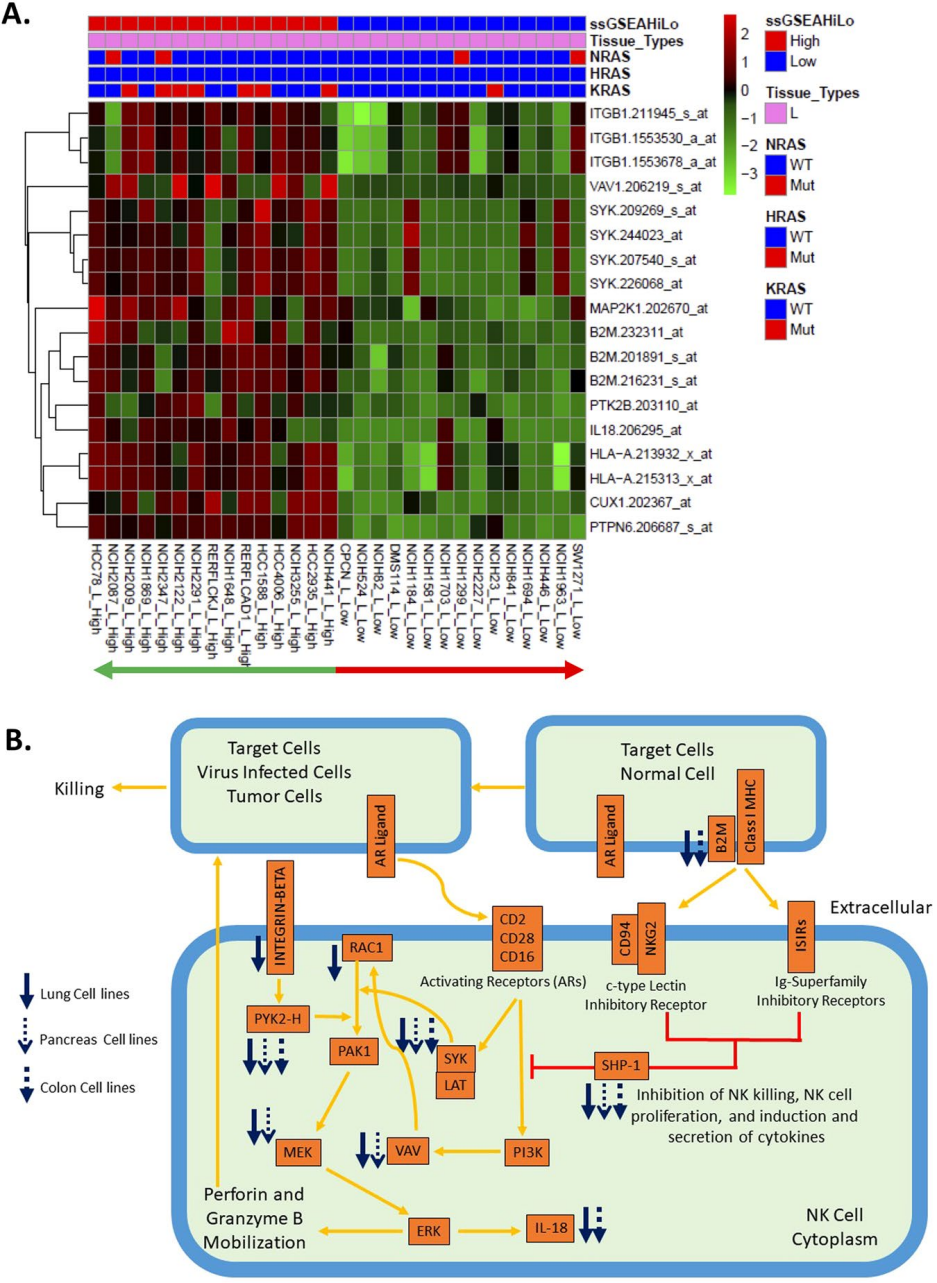
Differentially expressed genes from CCLE microarray data showed coordinated and consensus downregulated expression patterns in Ras-independent cell lines of three different tissue types, namely, lung, pancreas and colon, in a Biocarta pathway: Ras-independent pathway in NK cell-mediated cytotoxicity (Ras_Ind NK Pathway). (**A**) Heatmap of the differentially expressed genes (DEGs) in the Ras_Ind NK pathway between the top 15 vs. bottom 15 CCLE lung cell lines with the highest vs. lowest ssGSEA scores. DEGs are from L_High_vs_L_Low_AdjP0_05FC1_5 (detailed in Supplementary Table 1). (**B**) Pathway overlay of DEGs from all three tissue types (A, B, C) on the Ras_Ind NK pathway. Cell lines with high ssGSEA scores are similar to Ras-dependent lines, whereas those with low ssGSEA scores are similar to Ras-independent lines. Blue arrows show downregulated corresponding genes in the microarray data of CCLE cell lines with low ssGSEA scores (Ras-independent lines) for cell lines of different tissue origins. Gene aliases are as follows: MAP2K1 = MEK; B2M = beta-2-macroglobulin associated with MHC; PTK2B = PYK2; PTPN6 = SHP-1. Expression data used are CCLE microarray data.

To confirm the pathway PPEP analysis result (Fig. 3A–C) at the gene level and establish the biological relevance and connections with Ras dependency, a comprehensive analysis was performed on the genes in the canonical Biocarta pathway: “Ras-Independent pathway in NK cell”. First, the DEGs between the top 15 vs. bottom 15 CCLE cell lines with the highest or lowest ssGSEA scores derived using the EMT signatures were specifically retrieved for this pathway from microarray data for the three tissue types in which KRAS is believed to be heavily involved based on the high frequency of mutation: lung (Fig. 4A), pancreas (Supplementary Figure 9A), and colon (Supplementary Figure 9B). Overlay of the DEGs from all three tissue types on the map of this pathway clearly showed how strikingly consistent the expression pattern among these DEGs is, especially for the critical genes in each of the main signaling branch cascades that lead to Ras-independent MAP kinase activation: PYK2, SYK, VAV, and SHP-1, which converge at PAK1 (Fig. 4B). The downstream genes, such as MEK and IL-18, also show a markedly consistent response. Strikingly, although there is some variation in the composition of the DEGs from each tissue type, all DEGs are downregulated in Ras-independent lines across all three tissue types, with lung cell lines having the most consistent expression behavior across individual lines (Fig. 4B). This was confirmed with RNAseq data from CCLE cell lines from all three tissue types, and similar observations were made with the limma-voom method for RNAseq analysis (Supplementary Figure 10). Similar observations were also made using two other differential expression analysis methods: edgeR and DESeq2 for CCLE RNAseq data (Supplementary Figure 11). Considering the critical roles of SYK and PAK1 in this pathway and the fact that these two kinase genes are shared between the EMT signature and Ras-independent pathway in NK cells (Supplementary Table 3), these two genes may play a critical role in Ras dependency globally.

These observations were made in all three KRAS-associated tissue types. Interestingly, when three other tissue types in which KRAS is believed to be less involved were chosen for similar tests, we observed completely different results (Supplementary Figure 12), consistent with the pathway analysis results (Fig. 3C). For the total 17 genes in this pathway, only 2 for hematopoietic and lymphoid cell lines, 3 for breast cells, and none for skin cells were found to be DEGs (Supplementary Figure 12A,B; no gene to plot for skin) in contrast to 10 for lung, 5 for pancreas and 5 for colon cell lines (Fig. 4A, Supplementary Figure 9A,B, respectively). These results suggest that tissue specificity may exist for Ras dependency represented by the derived ssGSEA scores. However, there is also a possibility that the limited sample size in these tissue types led to little difference between the high and low classes in their Ras dependency. Independent datasets with a larger sample size would be needed to validate these possibilities.

Of course, identification of biological themes associated with Ras dependency in cell lines represents only a portion of the potential of using a computational estimation method. Similarly, it is likely that cell lines exhibit significant alterations from the tumor from which they were produced. Therefore, we wanted to determine whether we could compute similar metrics using actual tumor data and then apply those to assess consistency with our observed biological themes. To test whether these observations were also true for human cancer patient samples, RNAseq data from TCGA for three cancer types, namely, LUAD, PAAD, and COAD, were subjected to a similar DEG analysis between the top 15 vs. bottom 15 patient samples with the highest or lowest EMT signature-based ssGSEA scores. The retrieved DEGs from this pathway also showed very similar gene expression behavior (Supplementary Figure 13). Although slight variations are observed in the actual DEGs, the DEGs remain anchored at the main signaling branch cascades that lead to MAP kinase activation, showing very consistent expression patterns among all three cancer types (Supplementary Figure 13D). Furthermore, the expression pattern of these DEGs was very similar to that in CCLE cell lines (Fig. 4B and Supplementary Figure 10D) in that many of the genes at the main signaling branch cascades that lead to MAP kinase activation in this pathway were DEGs and all of these DEGs were consistently downregulated in the Ras-independent cell lines or tumors from cancer patients. With two other RNAseq differential expression analysis methods, similar observations were made (Supplementary Figure 14A,B). Interestingly, PAK1 was found as a DEG in TCGA data from both LUAD and PAAD patients (Supplementary Figure 13D, 14A,B, 15A,–C), whereas SYK was found as a DEG in CCLE data from lung, pancreas and colon cell lines (Fig. 4B, 10D) as well as in TCGA data from LUAD and PAAD patients (Supplementary Figure 13D, 15A), which collectively revealed that PAK1 and SYK indeed may be critical genes for downstream signaling in the context of Ras independency.

Interestingly, using TCGA BRCA, LAML, and SKCM data to run a similar analysis, relatively few DEGs were found for the same pathway except for SKCM. CCLE skin-derived cell lines (no single DEGs, see above) thus behaved differently from TCGA SKCM (7 DEGs) in terms of the number of DEGs in this pathway, which may suggest that differences between patient tumors and derived cell lines may be dependent upon the source tissue and perhaps may reflect context-dependent adaptation regimens for tumors from different tissues. However, overall, cell lines and cancer patients’ tumors showed relatively consistent expression profile behavior in this pathway, highlighting the potential connection between this pathway and Ras dependency.

To further test the robustness of these results, instead of the top 15 vs. the bottom 15 patient samples, a similar DEG analysis was performed using the top 30 vs. bottom 30 patient samples with the highest or lowest EMT signature-based ssGSEA scores from the three types of cancer. Regardless of the analysis method selected, similar observations were made (Supplementary Figure 15A–C).

Interestingly, although this pathway describes a reportedly Ras-independent MAP kinase cascade specific to NK cells, it is very likely that its activity is highly generic, acting as a common theme in various tissues and cell types, given that the behavior of genes in this pathway is common in cell lines from multiple tissue types and tumor samples from multiple cancer types, which were picked up in both microarray and RNAseq technology platforms. This is further confirmed by a tissue type-independent network view through a global database query and network analysis using GeneGO databases and a network tool in that both Ras-dependent and Ras-independent cascades may be context dependent and converge at MEK1/2 for downstream MAPK signaling (Supplementary Figure 15D). This Ras-independent cascade includes SYK, PAK1 and PI3K genes (Supplementary Figure 15D), consistent with the emerging notion described above that PAK1 and SYK are postulated as critical genes in the context of Ras independency as well as the role of PI3K in Ras independency suggested by others^9^.

Furthermore, in a recent gene essentiality profiling study^28^, a few genes were found to be synthetic lethal partners with oncogenic Ras genes, including the top-scoring gene PREX1, which encodes a guanine exchange factor (GEF) for RAC GTPases (e.g., RAC1) and works as an AML-specific activator of MAPK signaling. Notably, RAC1 is the key component for one of the signaling branches that converges at the PAK1 gene in the Ras-independent pathway in NK cell-mediated cytotoxicity from Biocarta (Fig. 4B, Supplementary Figure 10D). Although the Biocarta annotation of this pathway does not include PREX1 as a component of this pathway, likely due to the focus of its main theme of the Ras-independent pathway, the fact that PREX1 works as the GEF for RAC1 indicates its indispensable role in this pathway for Ras-independent signaling. Interestingly, PREX1 was indeed significantly upregulated in Ras-independent samples of TCGA PAAD patients (Supplementary Tables 4 and 5), with the opposite direction of alteration for RAC1 being suggestive of tight transcriptional regulation in RAC1 signaling. Similar trends of opposite expression patterns were also observed in COAD and LUAD, although the differences in gene expression were not as significant as those in PAAD (Supplementary Table 6). This transcriptionally complementary behavior between PREX1 and RAC1 is consistent with a previous report that PREX1 is negatively regulated by RAC1-activated PAKs at the phosphorylation level as a possible negative feedback mechanism in a Ras-independent cellular context^29^. In addition, other related genes, including SHOC2, RACGAP1, and RAC3, all showed significant transcriptional changes between Ras-dependent and Ras-independent samples in various ways from COAD and LUAD (Supplementary Tables 4 and 5), indicative of the diversity of regulatory mechanisms for this pathway in different cellular and tissue contexts. In the cell line data, we only observed transcriptional alternation in cell lines of lung tissue for the RAC1 gene (Fig. 4B, Supplementary 10D). However, we also observed that PREX1 genes were significantly altered between Ras-dependent and Ras-independent lines but in the opposite direction from RAC1 in lung cell lines in both RNAseq and microarray data (Supplementary Tables 7 and 8), although in microarray data, RAC2 was found to be a DEG rather than RAC1. In cell lines of pancreas and colon tissues, various RAC1-related genes were identified as DEGs, which may compensate for the roles of RAC1 and PREX1 in corresponding cellular and tissue contexts. All the evidence suggests the interplay between PREX1 and RAC1 and their potentially critical role in Ras-independent MAPK signaling, consistent with the uncovered synthetic lethality with RAS genes in the previous study^28^.

### Comprehensive expression analysis of the canonical Biocarta Fas signaling pathway recapitulated the role of Fas in Ras dependency

Fas and its mediation of apoptosis have been reported to be involved in the killing of lung cancer cells upon triggered sensitization and possibly Ras dependency regulation^27^. As with the NK pathway, to confirm the pathway PPEP analysis result (Fig. 3A,B) at the gene level and study the biological relevance and connections with Ras dependency for this pathway, a comprehensive DEG analysis was also performed on the genes in the canonical Biocarta pathway: “Fas signaling pathway”. DEGs were retrieved from this pathway between the top 15 vs. bottom 15 CCLE lung cell lines with the highest vs. lowest EMT signature-derived ssGSEA scores. The results showed a coordinated and consistent downregulated expression pattern for multiple probes of the FAS gene and many caspase genes in Ras-independent lung cell lines (Supplementary Figure 16A). After close examination of the behavior of the DEGs from microarray data in the context of this pathway, surprisingly, FAS and its immediate downstream signaling gene FLICE and many of the caspases all showed downregulation in the Ras-independent lung cell lines (Supplementary Figure 16B). With CCLE RNAseq data analyzed by three different methods, the DEGs derived from all three methods also showed very consistent patterns in that the FAS gene and its immediate downstream signaling gene FLICE and many of the caspases all showed downregulation in the Ras-independent lung cell lines (Supplementary Figure 16C). These patterns were further confirmed by the expression levels of DEGs in the selected individual cell lines for all three analysis methods for RNAseq data (Supplementary Figure 17A–C).

As with the NK pathway described above, to determine whether these phenomena can be observed in human cancer patient samples, RNAseq data from LUAD tumors from TCGA cancer patients were subjected to a similar DEG analysis using the top 15 vs. the bottom 15 patient samples with highest or lowest ssGSEA scores using the EMT signature. Interestingly, all of the critical DEGs in the pathway derived from the three analysis methods, including FAS, FLICE, and some of the caspases (Caspase 7 and Caspase 10), were consistently downregulated in Ras-independent LUAD patient samples (Supplementary Figures 18A–C), which is evident in the pathway view overlaid with DEGs (Supplementary Figure 18D).

The PPEP pathway analysis showed that the lung was the only tissue type in which the Fas signaling pathway was significantly enriched (Fig. 3A,B). In contrast, the other Biocarta pathway described earlier, “Ras-Independent pathway in NK cell”, showed a tissue context-independent response for KRAS-associated tissue types. We wanted to assess whether the trends observed in the colon and pancreas were consistent even though they did not reach statistical significance for pathway level enrichment. Interestingly, upstream signaling genes, including FAS, FLICE, and caspases, generally showed similar and consistent downregulation patterns in Ras-independent samples from PAAD and COAD, consistent with LUAD, regardless of the analysis method, although higher variation in the behavior of downstream genes was observed (Supplementary Figure 19A–C). As above, when the sample size was increased to the top 30 vs. bottom 30 samples with the highest or lowest EMT signature-based ssGSEA scores, essentially similar results were observed (Supplementary Figure 20A–C). These observations indicate that this pathway may be associated with RAS dependency more generally in other tissues, as was the case with the NK pathway, albeit weaker than it was in lung samples.

### ssGSEA scores derived using the Ras dependency signatures and Ras pathway genes are associated with cancer patient survival outcomes

To explore the potential connection between the ssGSEA scores derived from the Ras dependency signatures and cancer patient survival outcomes, the in-house survival analysis method GradientScanSurv^18^ was applied to the TCGA data. Our method was demonstrated to have better performance than other methods for association assessment of continuous variables such as gene expression data with censored survival outcome^18^. The ssGSEA scores derived from the Ras dependency signatures are also continuous variables, which are suitable for such an association test with our GradientScanSurv method. Ras pathway genes were used as a control for comparison.

**Figure 5:**
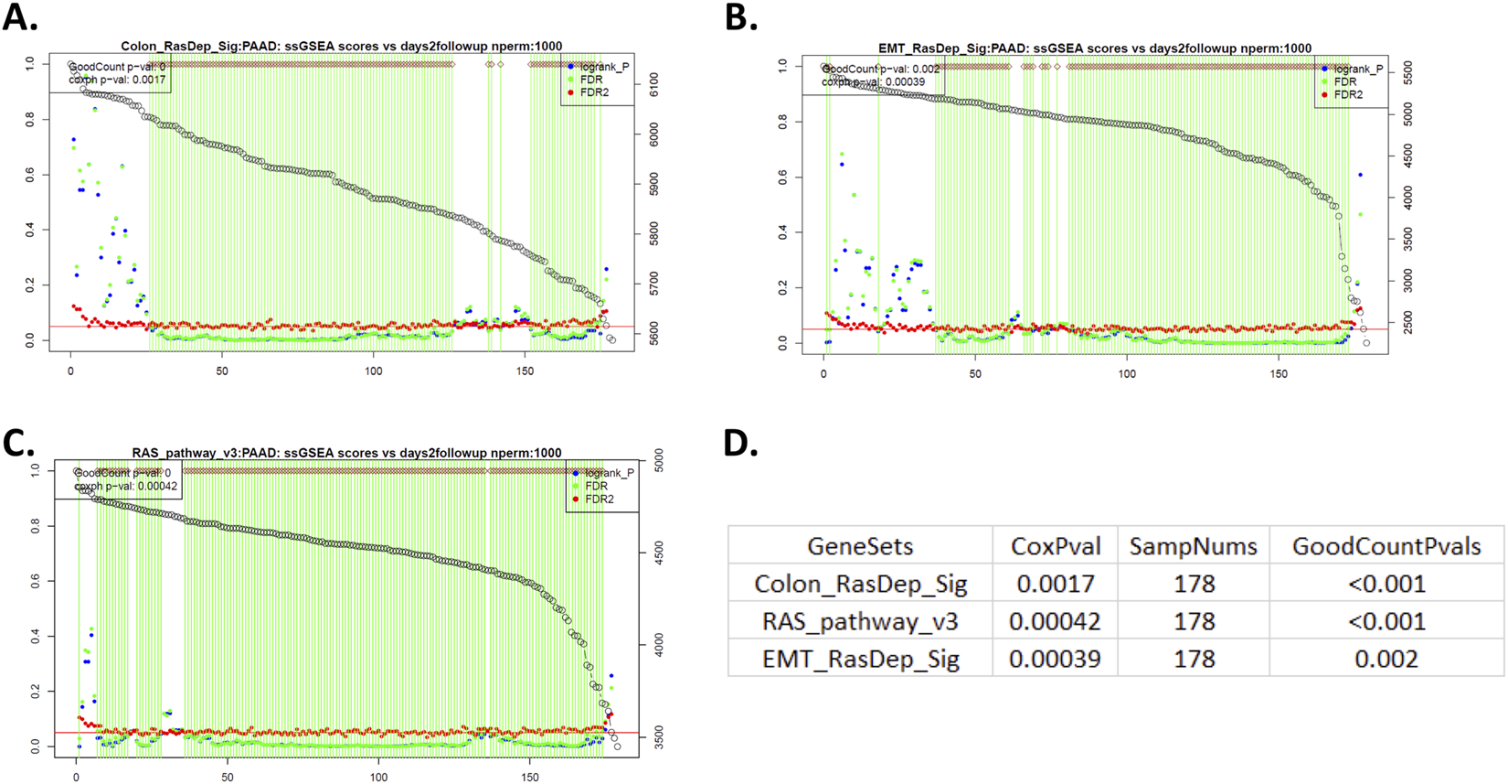
GradientScanSurv results in TCGA PAAD data (pancreatic adenocarcinoma) using ssGSEA scores of RDI gene signatures and the RAS pathway. Survival-gradient plots derived from the GradientScanSurv method with gradients of selected gene signatures: (**A**) Colon_RasDep_Sig: colon signature (Singh *et al*. 2012); (**B**) EMT_RasDep_Sig: EMT signatures; (**C**) RAS_pathway_v3 (Ras pathway genes annotated from Ras Central). (**D**) Statistical summary in the table, where GoodCountPvals were derived from the GradientScanSurv method, showing that all three signatures’ ssGSEA scores have a significant association with patients’ survival outcomes. SampNums: numbers of samples in the dataset; CoxPval: Cox regression model-derived p-values.

As expected, the ssGSEA scores derived from the Ras pathway genes showed a significant association with pancreas cancer patient survival outcomes in TCGA PAAD data (Fig. 5), which is consistent with the initial observation that many individual Ras pathway genes have a significant association with patient survival outcome^18^. This shows that the aggregate scores derived from the ssGSEA method indeed may faithfully reflect the combined impact of individual genes if many of the genes already show significant association individually, indicating the power of the ssGSEA method and its potential broad application. Interestingly and surprisingly, ssGSEA scores from the EMT signature and the colon signature were also significantly associated with PAAD cancer patients’ survival outcome (Fig. 5A,B), postulating that Ras dependency has a significant impact on pancreatic cancer patient prognosis as a potential clinical application of these derived scores. As expected, in the TCGA LUAD and KIRC data, the ssGSEA scores from the Ras pathway genes also showed a significant association with survival outcome (Supplementary Figure 21 and 22). However, only those of the colon signature but not the EMT signature showed a significant association in the LUAD data (Supplementary Figure 21). In contrast, only those of the EMT signature but not the colon signature showed a significant association in the KIRC data (Supplementary Figure 22). These results collectively suggest that ssGSEA scores derived with the EMT signature and the colon signature that correlated with each other at a moderate level may have tissue specificity as a component of their relationship with Ras dependency, considering the multiple tissue origins of the EMT signature^7^ compared with restricted colon origin of colon signature^8^. In contrast, none of the three signatures’ ssGSEA scores showed significant association with survival outcome in TCGA COAD colon cancer data (Supplementary Figure 23), which was consistent with the initial observation that in colon cancer, there are no Ras pathway genes that show significant association with patient survival outcome^18^. We surmise that this may be due to mixed subtypes among patients with COAD, which is a highly clinically heterogeneous cancer type. Data from a larger study may be needed to validate these results and investigate the relationship between Ras dependency and survival outcome and their potential in the prediction of cancer patients’ prognosis for the colon subtype(s).

## Discussion

The very high frequency of observation of perturbations of genes in the RAS pathway through mutation and copy number alterations confirms the involvement of the pathway in many cancers. Therefore, further understanding of the biology that underlies the concept of Ras dependency is an important objective. In this report, we sought to derive and apply Ras-dependency-related gene signatures and expression data to derive ssGSEA score-based RDIs for the full panel of cell lines from CCLE in a computational manner by utilizing the single-sample GSEA (ssGSEA) method^11^. Surprisingly, the derived ssGSEA scores, specifically from the EMT signature, showed excellent correlation with experimentally measured RDIs compared to other signatures that we evaluated. We also found that the ssGSEA scores derived using microarray and RNAseq expression data were highly correlated with the experimentally measured RDIs, making our observations independent of the data source and platform applied. The correlation levels and significance that we observed rule out all but the remotest possibility that these observations occurred by chance, which is consistent with the notion that the computationally derived dependency indexes indeed recapitulate those that are experimentally derived. In addition, the indexes were very specific to the EMT signature-based ssGSEA scores, which are superior to any control KEGG and Biocarta pathway gene list or other RAS-dependent or RAS-related gene signatures that we evaluated. This finding further confirms the hypothesis that Ras dependency may be determined by the EMT (epithelial to mesenchymal transition) or MET (mesenchymal to epithelial transition) status, which is consistent with the original viewpoint about RAS dependency^7^.

Furthermore, the computationally derived RDIs indeed showed high correlations and connections with previous relevant studies, including a siREN in-house study^14^, and modest-to-low levels of correlation with an external high-throughput RNAi screening study and CRISPR studies from the Broad Institute^15,16^. The large overlap of the genes and high correlation of the derived ssGSEA scores between the EMT signature and the siREN study-derived gene signatures indicate a strong biological connection, given that these data were derived from two independent studies with completely different technologies. The high concordance between the EMT and colon signature-derived ssGSEA scores with the RSK/KRAS classification in the siREN study also indicates the strong connection between Ras dependency and the identified RSK/KRAS subtypes. The cancer dependency map-based data from the Broad Institute showed modest levels of correlation with the EMT signature-derived ssGSEA scores but with high statistical significance, providing another line of evidence showing that the EMT-derived ssGSEA scores indeed reflect the Ras dependency level metric of each cancer cell line.

Perhaps more surprisingly, the computationally derived RDIs showed applicability beyond cell lines to include human cancer patient samples. The extension to both cell lines perhaps not conventionally thought to involve Ras dependency as well as patient samples significantly extends the range for which this concept can be experimentally assessed and further investigated. Thus, this study has shown that the ssGSEA method can be very useful for ranking certain behaviors of cell lines or samples using any related signature and gene expression data.

By selecting cell lines representing the extremes in Ras dependency and then characterizing DEGs between them, we probed the biological themes underlying Ras dependence. Expression profiling showed significant involvement of two Biocarta pathways, namely, the Ras-independent pathway in NK cell-mediated cytotoxicity and the Fas signaling pathway, although other themes were observed to a lesser extent. The other identified pathways are potentially also interesting and warrant further investigation.

The first pathway is annotated specifically for the Ras-independent MAP kinase activation that occurs in the NK cell context. However, we found that the genes in this pathway are not NK-cell specific; we observed consistent expression patterns for these genes in CCLE cell lines from multiple tissue types and in TCGA patient samples regardless of the data source, technology platform or differential expression analysis method applied to RNAseq data. Thus, it seems likely that a generic Ras-independent activation pathway exists for various tissue types or cell types that may comprise the same components as the one annotated for NK cells. We also identified an aspect of tissue specificity for Ras dependency, where we observed consistent gene expression profile alterations in KRAS-associated tissue types, including lung, pancreas, and colon, but not in other tissue types, in which KRAS may be less involved, such as breast. We propose that this pathway may constitute a global mechanism underlying Ras-independent signaling for MAP kinase activation and that its “wake-up” status may depend upon the cellular context, tumor status, and tumor stage as well as the environmental context. The fact that we see the involvement of this pathway even in tumor-derived cell line data suggests that the observed expression is derived from tumor cells and not immune infiltrating cells. Again, this is indicative of the more global and generic nature of this pathway across a wide range of tissue or cell types beyond NK cells. Interestingly, it was initially reported that KRAS knockout in cells revealed an alternative bypass mechanism supported by noncanonical MAPK signaling, and nearly all KRAS-deficient cells exhibited PI3K-dependent MAPK signaling and induced sensitivity to PI3K inhibitors^9^. Consistent with this initial report, the gene expression patterns of this pathway in our data revealed that PI3K is located upstream in this putative generic Ras-independent pathway and plays a role in Ras-independent MAPK signaling. Furthermore, our observation that this Ras-independent signaling pathway includes SYK, PAK1 and PI3K as the critical genes in the context of Ras independency provides additional insights into the role of PI3K in the Ras independency postulated by the initial report^9^. Lastly, the uncovered fact that two critical kinase genes, namely, SYK and PAK1, are the only shared genes between the EMT signature and Ras-independent pathway in NK cells also highlights the underlying biological connections between the computational RDIs and Ras-independent contexts.

Interestingly, we also observed complementary differential expression patterns of the RAC1 and PREX1 genes, which are among the critical upstream driving components in the pathway and its GEF, respectively, in TCGA PAAD patient samples and CCLE lung cell lines and various potential make-up expression schemes from other RAC1-related genes in our data. This is highly consistent with the uncovered synthetic lethality scheme of RAC1/PREX1 and Ras genes as well as the postulated PI3K/VAV-triggered and RAC1/PREX1-mediated MAPK signaling in a Ras-independent context from a previous essentiality profiling study^28^, which is also depicted as one of the schemes for Ras-independent MAPK signaling in this Biocarta pathway. This provides additional support for MAPK signaling in Ras-independent contexts that were defined by our ssGSEA score-based computational Ras dependency index. In addition, the previously observed feedback mechanism at the phosphorylation level between PREX and PAK^29^ is also consistent with our gene expression analysis in that these genes may have a tight regulation mechanism in Ras-independent contexts.

It should be noted that many of the previous studies did uncover the biological connections of these genes piece by piece. However, these previous studies did not indicate the role of PAK in the activation of downstream MAPK signaling in a RAS-independent context, as uncovered by our study. In addition, the Ras-independent pathway in NK cell-mediated cytotoxicity from Biocarta that depicts PAK as the convergence point for downstream MAPK signaling was originally found and described in NK cells. The integrative and comprehensive data mining and analysis in our study revealed such a scenario to be highly generic in Ras-independent contexts that can be defined by our computational RDIs. This is our novel notion for the field, that is, one can use computational RDIs to define the Ras dependency of a biological system with great application potential in both research and clinical settings. Furthermore, our report emphasizes the whole picture of these connections of these critical gene players in Ras-dependent or Ras-independent contexts to help understand and investigate their interplay, which we believe may help reveal the potential mechanisms of drug resistance for Ras-related treatment, given that Ras dependency and drug resistance are believed to be interconnected^7,8^. Finally, the involvement of mediators of this pathway may be consistent with tumor cells transmitting a signal that does impact immune cell activity in the context of a tumor environment with EMT involvement, which is consistent with a recent report^30^.

Interestingly, although more specific for lung tissue in our statistically assessed pathway enrichment analysis, the second pathway, namely, “Fas signaling pathway”, did show highly generic behavior at the gene level, especially for the upstream signaling genes, including FAS, FLICE and many caspases, in pancreas and colon samples in addition to lung samples. This may also suggest a highly generic role of FAS and FAS-mediated apoptosis in Ras dependency, which is consistent with a previous report^27^. Taken together, these two pathways may play essential roles in Ras dependency and represent or be associated with the underlying mechanisms that seem worthy of further investigation and experimental validation.

It is intriguing that the dynamic range of the derived ssGSEA scores is very broad and not limited to tissues/samples in which RAS dependency would be expected to play a role, thus permitting easy delineation of the high and low ends of the spectrum characteristic of the Ras-dependent and Ras-independent natures, respectively. This allows for assessment of the relative strength of the Ras dependency or Ras independency values as a continuum rather than as discrete categories. We also observed that the KRAS mutation status showed no connection with Ras dependency, suggesting that Ras dependency may be a unique feature that might allow us to assess a patient’s prognosis for drug resistance in the clinical setting beyond the conventional scenarios in which Ras gene involvement might be considered a priority (e.g., pancreas, colon and lung tumors for KRAS and possibly melanoma for NRAS). It will be interesting to evaluate the copy number and other genomic changes beyond Ras mutation status in this new light. It should be noted that some of our observations indicate that such Ras dependency may be tissue specific, and this tissue dependency may be linked to the levels of KRAS involvement in the tumors of such tissue types, although larger sample sizes and independent datasets may be needed to confirm these observations.

In addition, the large overlap between the dynamic ranges of the EMT signature-based ssGSEA scores across a variety of tissue types suggests that Ras dependency may not just operate in tissue types in which KRAS was conventionally believed to play a major role, such as pancreas, lung, and colon. In fact, our recent report showed that KRAS or RAS pathway genes not only play a critical role in these major “RAS-engaged” tissue types but also very likely play important roles in many other tissue types^4^. This is also supported by our initial analysis that assessed the impact of mutations, copy number alternations and elevated gene expression of RAS pathway genes on individual patients across all TCGA tumor types and found that the vast majority, if not all, of the TCGA cancer patients were “hit” one way or another by RAS pathway genes (unpublished observations). Ras dependency may be an intrinsic nature of all cell types, and it is the “wake-up” status of the intended cells that determines whether Ras dependency becomes an influential factor for the behavior of these cells dependent upon their cellular and environmental contexts.

The association between the EMT signature or colon signature-based ssGSEA scores with TCGA cancer patients’ survival outcomes in various tumor types shed further light on the importance of Ras dependency in cancer patient prognosis and drug resistance during treatment. This is indeed the first line of direct evidence showing a strong connection between Ras dependency and cancer patient survival outcome and its clinical application potential, although additional independent datasets with larger sample numbers may be needed to further validate the connections established here.

This is the first computational application that uses genome-wide gene expression profiling to represent RDIs. Conceivably, such a gene signature-based RDI scoring system can be essentially applied to cancer patients’ tumors using gene expression data. Consequently, we may be able to evaluate the ability to use EMT signature-based RDIs as a biomarker to predict the sensitivity of individual patient tumors to KRAS inhibition, a currently unmet need for effective therapeutic translation. In the context of the target of delivering precision medicine, it seems likely that a Ras dependency index can represent one dimension in a complex interplay of “usual” suspects that, when fully integrated, can help to correctly type a patient’s optimal treatment regimen. We encourage others in the community to investigate the relationships of these two pathways with Ras dependency and validate our findings that may help expedite our understanding of drug resistance in terms of the underlying mechanisms for Ras dependency and potential interaction with the immune system as well as the clinical values of such computational RDIs.

## Supplementary information

Supplementary Information File.

## Data Availability

The datasets generated during and/or analysed during the current study are available from the corresponding author on reasonable request.
