# Supplementary material for: ssGSEA score-based Ras dependency indexes derived from gene expression data reveal potential Ras addiction mechanisms with possible clinical implications
Source: Sci Rep. 2020 Jun 24;10:10258. doi: 10.1038/s41598-020-66986-8 (PMC7314760; doi:10.1038/s41598-020-66986-8)
Supplement: Supplementary file 1 — Supplementary Information File. [file 41598_2020_66986_MOESM1_ESM.docx]

**Supplementary Information File for:**

**ssGSEA score-based Ras dependency indexes derived from gene expression data reveal potential Ras addiction mechanisms with possible clinical implications**

Ming Yi^1,^*, Dwight V. Nissley^1^, Frank McCormick^1,2^, Robert M. Stephens^1,^*

^1^NCI RAS Initiative, Cancer Research Technology Program, Frederick National Laboratory for Cancer Research, Frederick, MD, USA.

^2^UCSF Helen Diller Family Comprehensive Cancer Center, San Francisco, CA, USA.

*Corresponding Authors

This document included Supplementary Figures 1 to 23 and Supplementary Tables 1 to 8


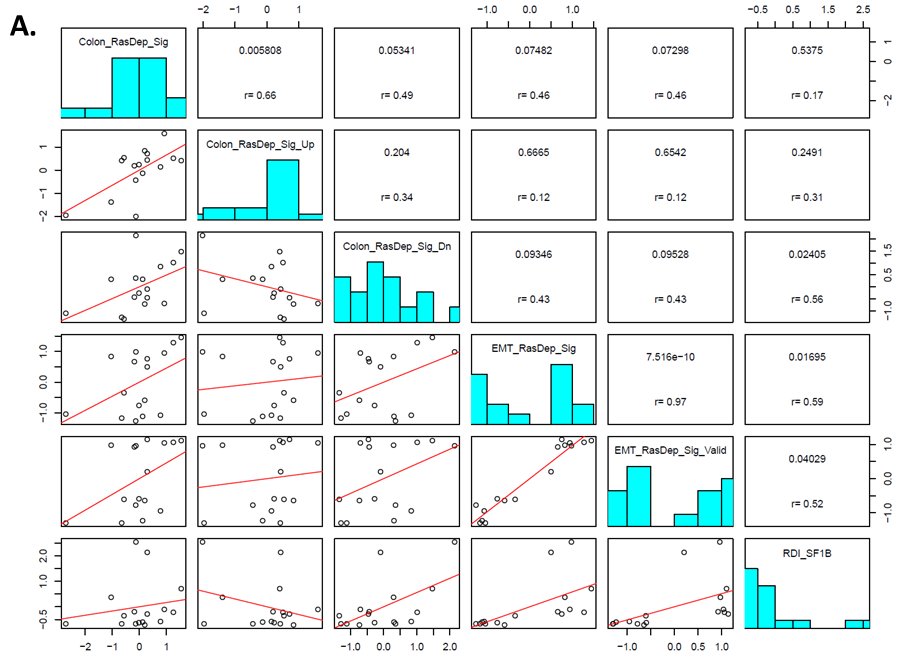


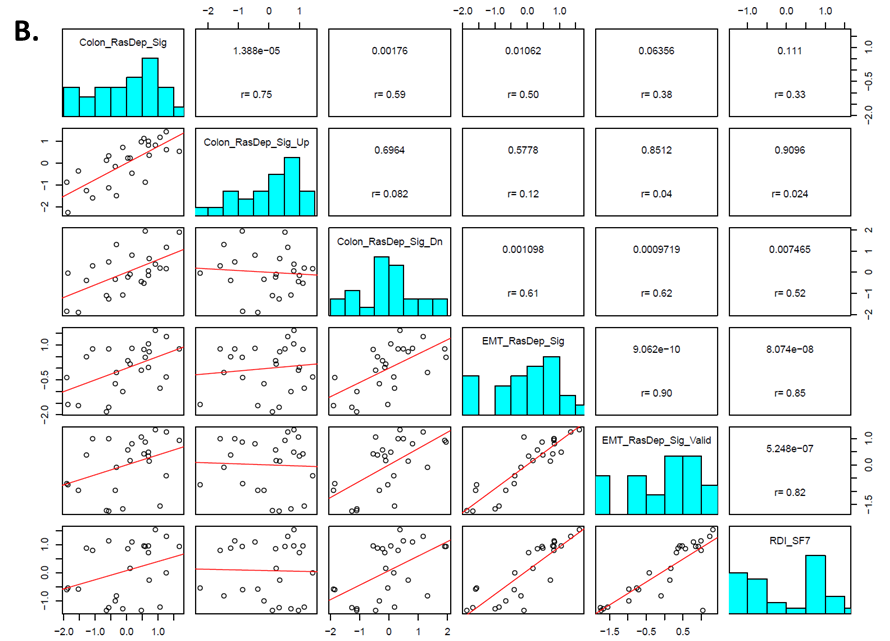


**
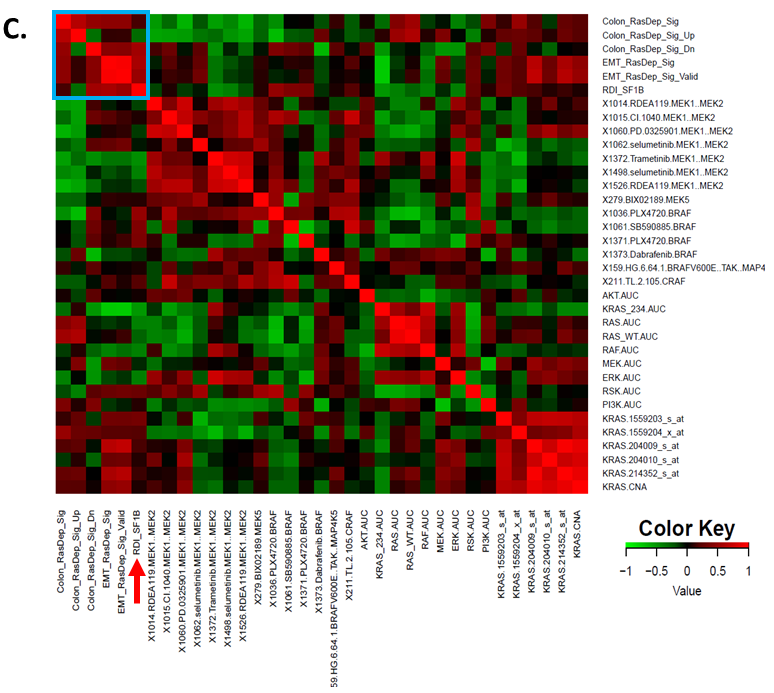
**

**
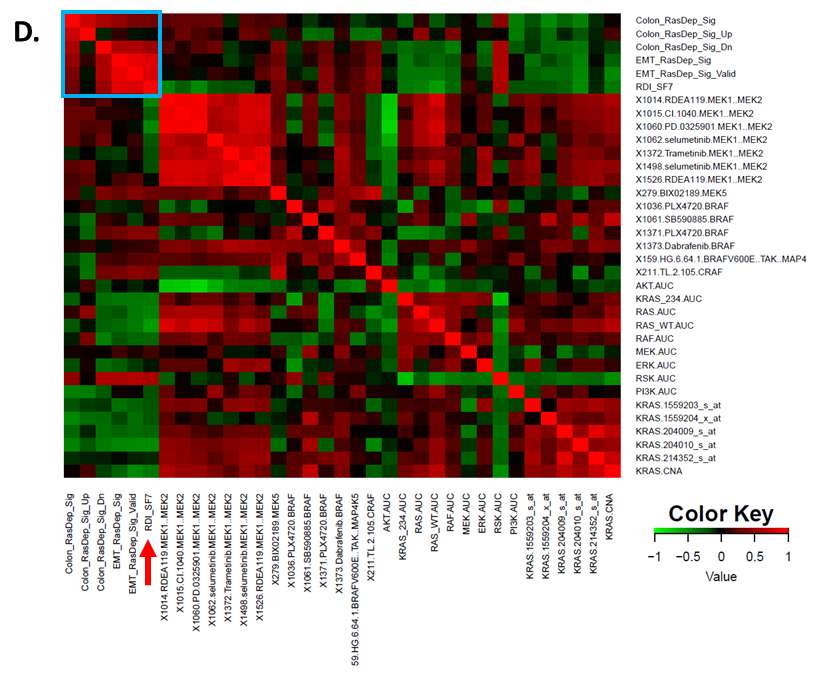
**

**Supplementary Figure 1**. Correlation details and correlation matrix of the ssGSEA scores of selected signatures and RDIs. **A.** Correlation details of the selected signatures and RDI_SF1B from Figure 1A. RDI_SF1B is described in Methods section. Left and bottom panel showed scatter plot of actual data points; right and top panel showed actual statistics of correlation including p-value and absolute values of correlation coefficients of each pair. **B.** Correlation details of the ssGSEA scores of selected signatures and RDI_SF7 from Figure 1B. RDI_SF7 is described in Methods section. Left and bottom panel showed scatter plot of actual data points; right and top panel showed actual statistics of correlation including p-value and absolute values of correlation coefficients of each pair. **C.** Heatmap of correlation matrix for all data from Fig 1A and various control data. RDI_SF1B is indicated by a red arrow. **D.** Heatmap of correlation matrix for all data from Fig 1B and various control data. RDI_SF7 is indicated by a red arrow. Control data included from some MEK or RAF inhibitors’ IC50 data, siren EGFP channel AUCs data of chosen nodes (Yuan et al 2018^14^), and KRAS microarray and copy number data from CCLE that were used as controls for purpose of comparison. Blue boxes highlighted the comparison between ssGSEA scores of different signatures.


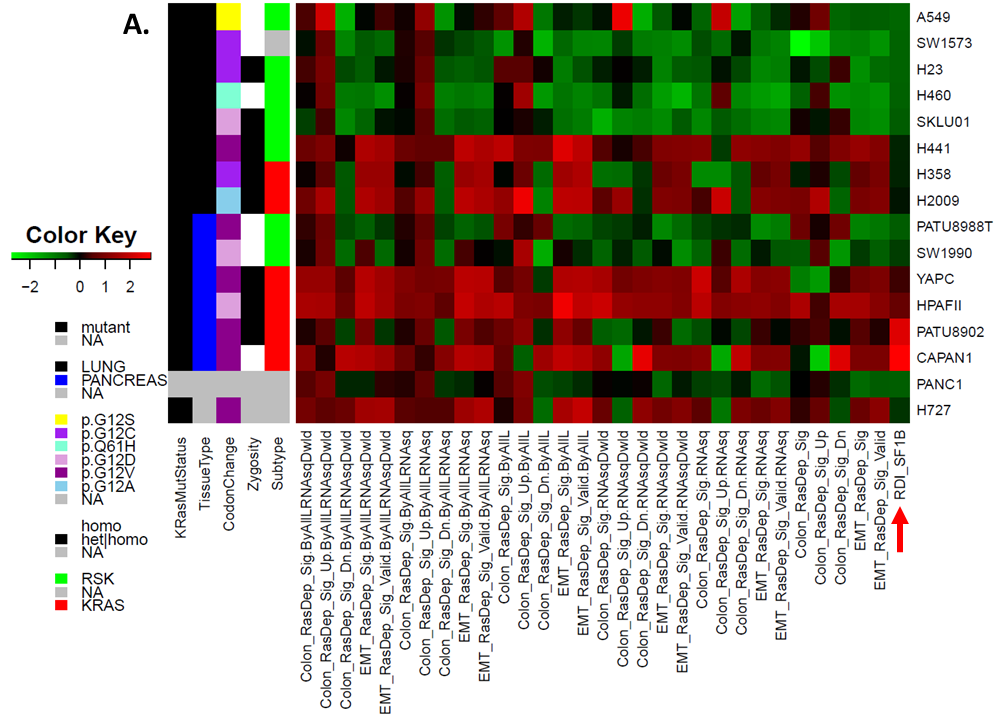


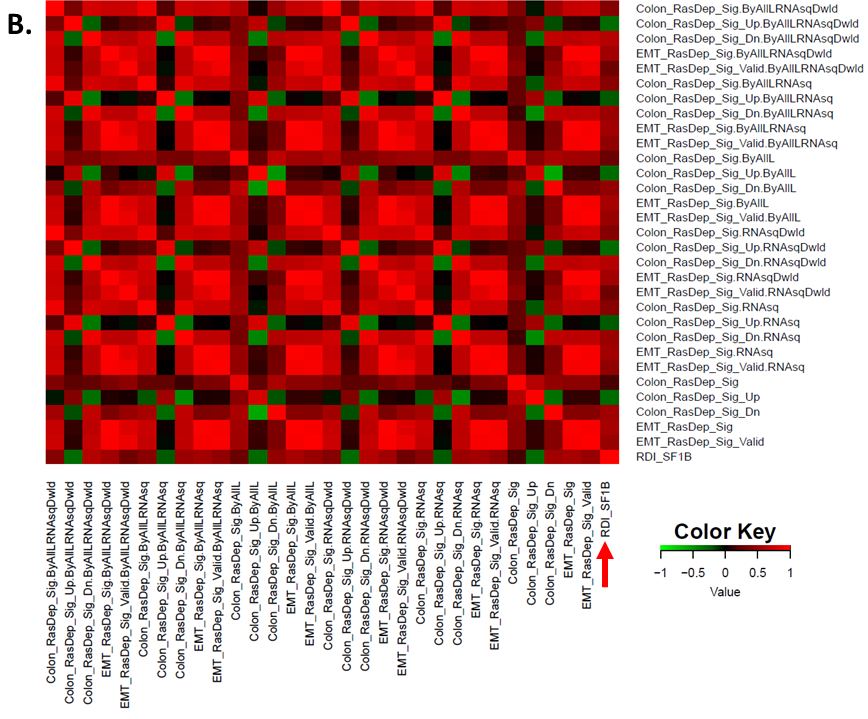


**Supplementary Figure 2**. Comparison of derived ssGSEA scores and Ras Dependency Indexes (RDIs) showed overall consistent and correlation patterns regardless of data resources and technology platforms. **A.** Derived ssGSEA scores and experimentally measured Ras Dependency Indexes (RDI_SF1B, red arrow, see Methods section) showed overall consistent patterns in heatmap regardless of data resources and technology platforms. ssGSEA scores for each cell line were computationally derived using EMT signatures, Colon Signature and also standardized into z-scores across cell lines the same as used in the previous study^7^ or of all CCLE lines (ByAllL) using expression data either of microarray or of RNAseq data (RNAsq). RNAseq data can be directly downloaded (RNAseqDwld) or processed by ourselves (RNAseq). **B.** Heatmap of correlation matrix for all data from **A**. RDI_SF1B is indicated by a red arrow.


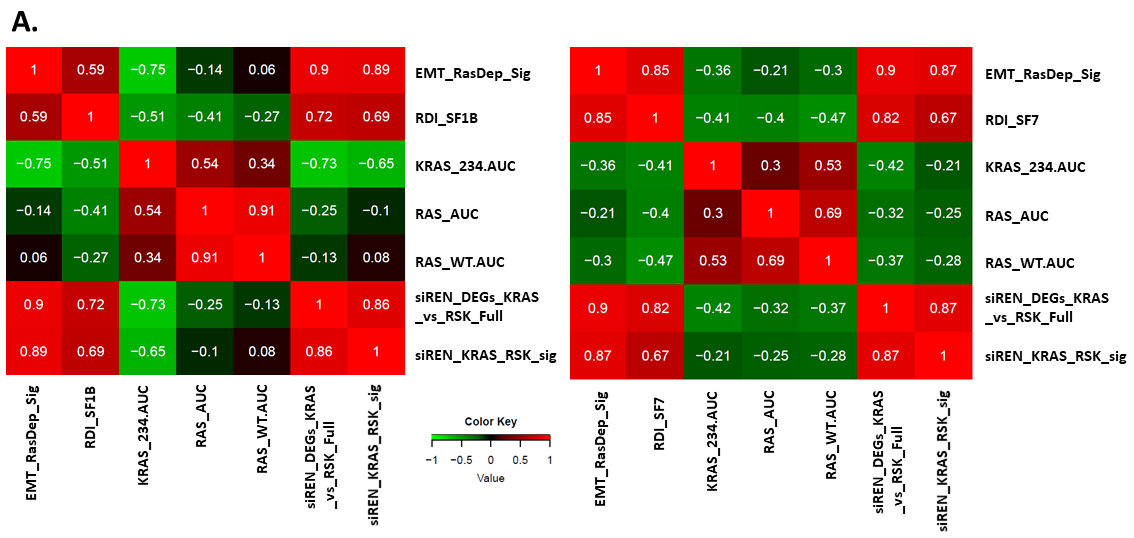


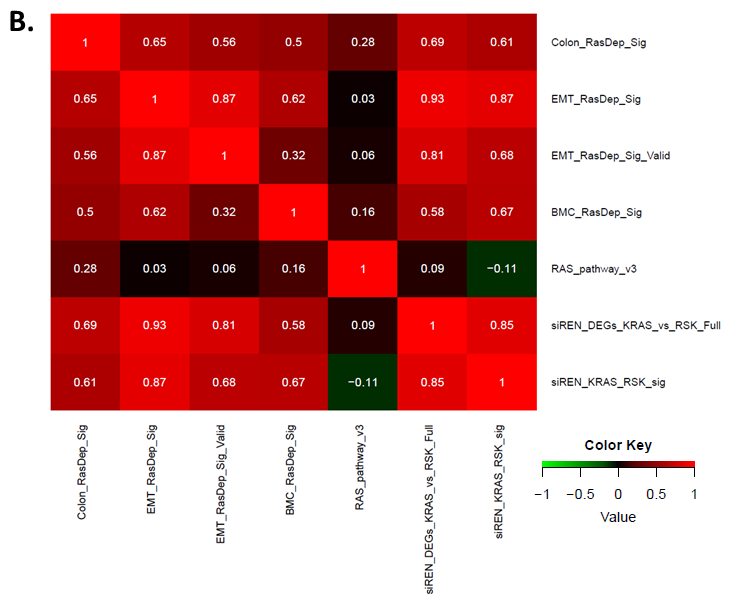


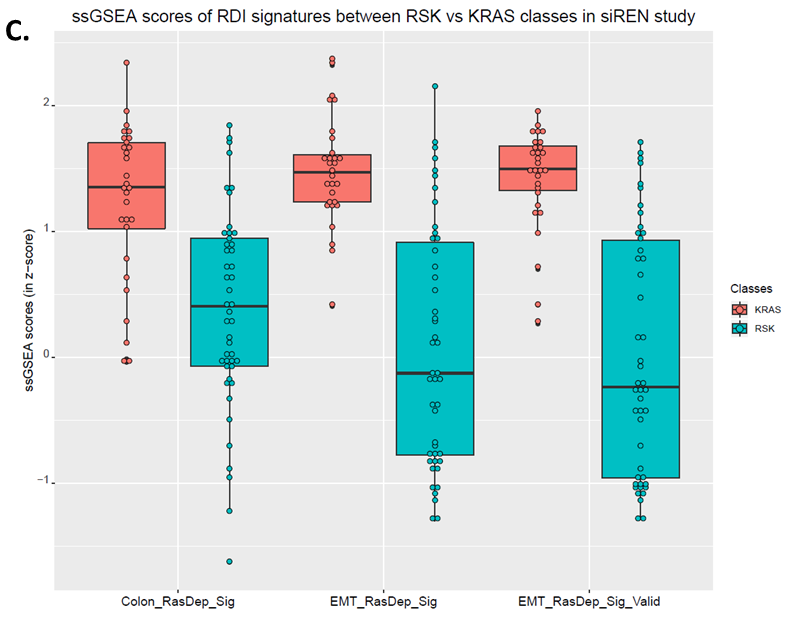


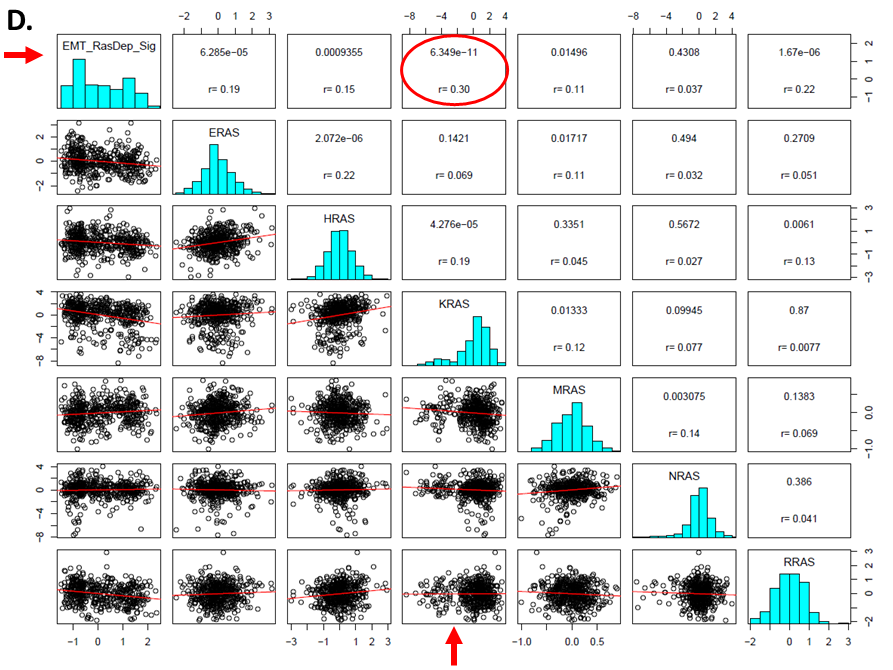


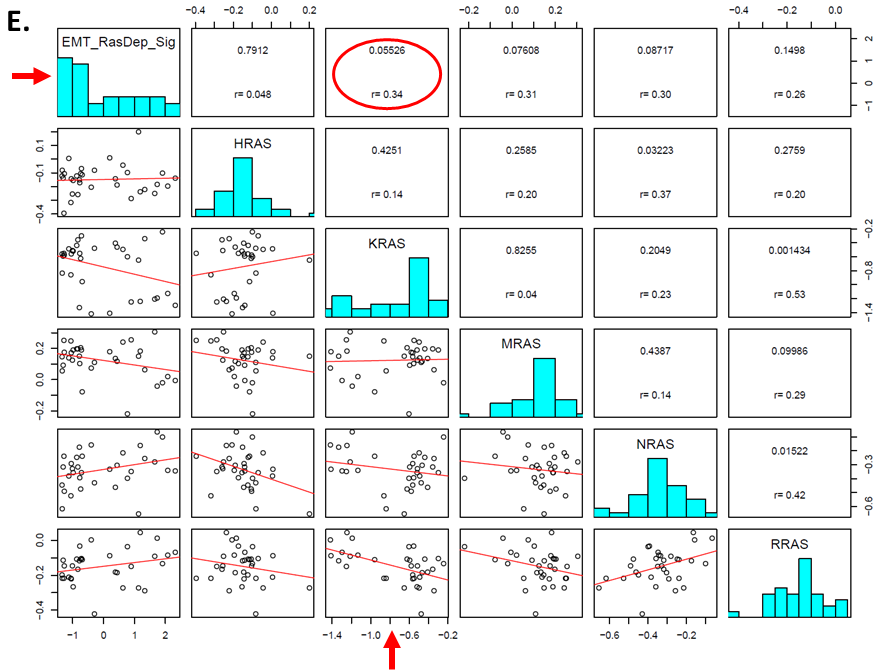


**Supplementary Figure 3.** Biological connections of ssGSEA scores of selected signatures and RDIs with relevant studies. **A.** Heatmap of correlation matrix of RDIs (RDI_SF1B left panel, RDI_SF7 right panel), siren EGFP channel AUCs data of KRAS nodes (Table S3 of Yuan et al 2018^14^), ssGSEA scores derived from EMT signatures and KRAS_RSK signatures (siREN_DEGs_KRAS_vs_RSK_Full and siREN_KRAS_vs_RSK_sig are differential expressed genes between KRAS- and RSK-type lines and EN-derived gene signatures respectively from Table S4 in Yuan et al 2018^14^) within the cell lines having measured RDIs (Singh et al 2009^7^). **B.** Heatmap of correlation matrix of ssGSEA scores derived from EMT signatures, Colon Signature, KRAS_RSK signatures (Yuan et al 2018^14^; ), RAS pathway gene signatures (Ras central) and BMC Ras signature^19^ within all 1037 CCLE cell lines with microarray expression data. **C.** Distribution of ssGSEA scores of EMT and Colon signatures between RSK and KRAS classes defined from a previous in-house siREN study^14^. **D.** Modest correlation with highly statistical significance between BROAD Institute’s cancer dependency DEMETER scores^15^ of KRAS gene and EMT signature ssGSEA scores in total 460 CCLE cell lines in common, indicated by red circle and red arrows. **E.** Modest correlation with marginal statistical significance between BROAD Institute’s cancer dependency CERES scores^16^ of KRAS gene at r=(-0.34) with p-value =0.055 and EMT signature ssGSEA scores in only 33 CCLE cell lines in common, indicated by red circle and red arrows

**
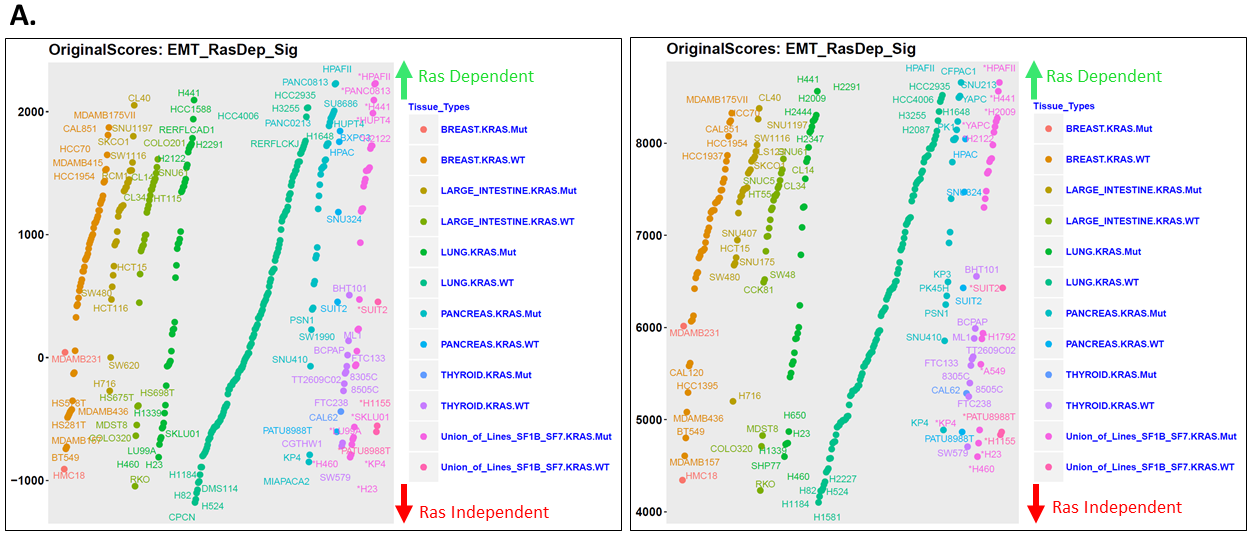
**

**
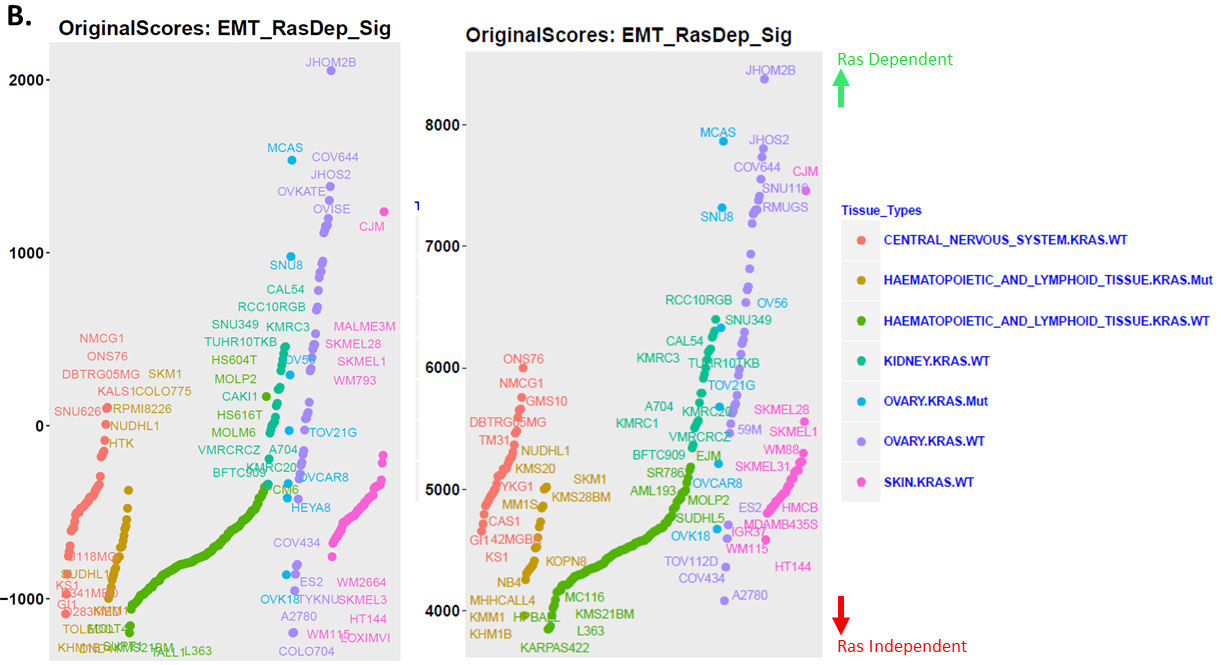
**

**
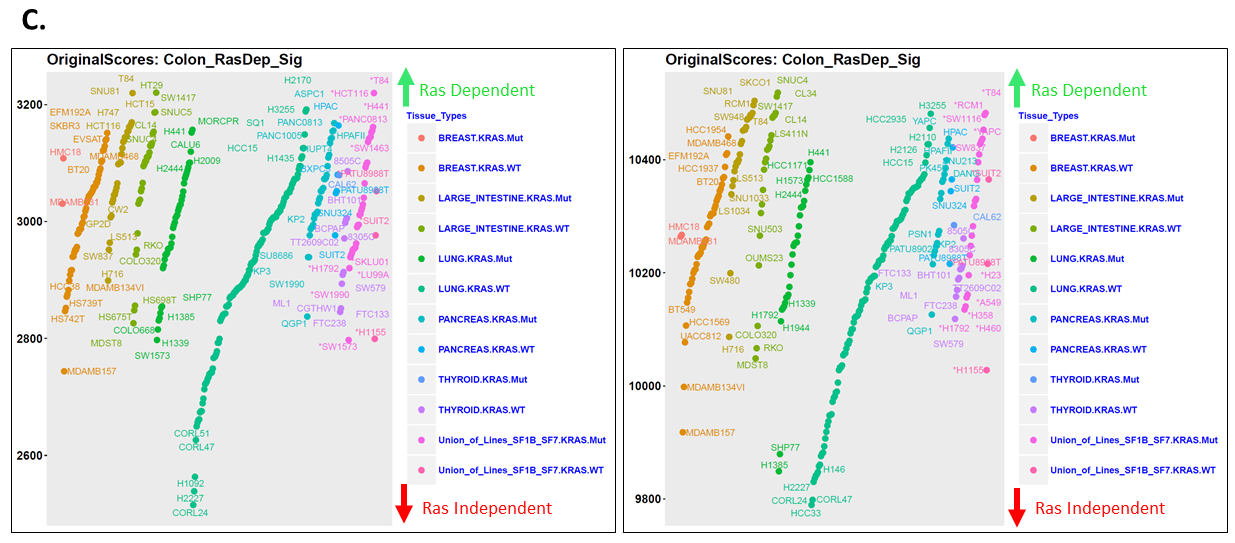
**

**
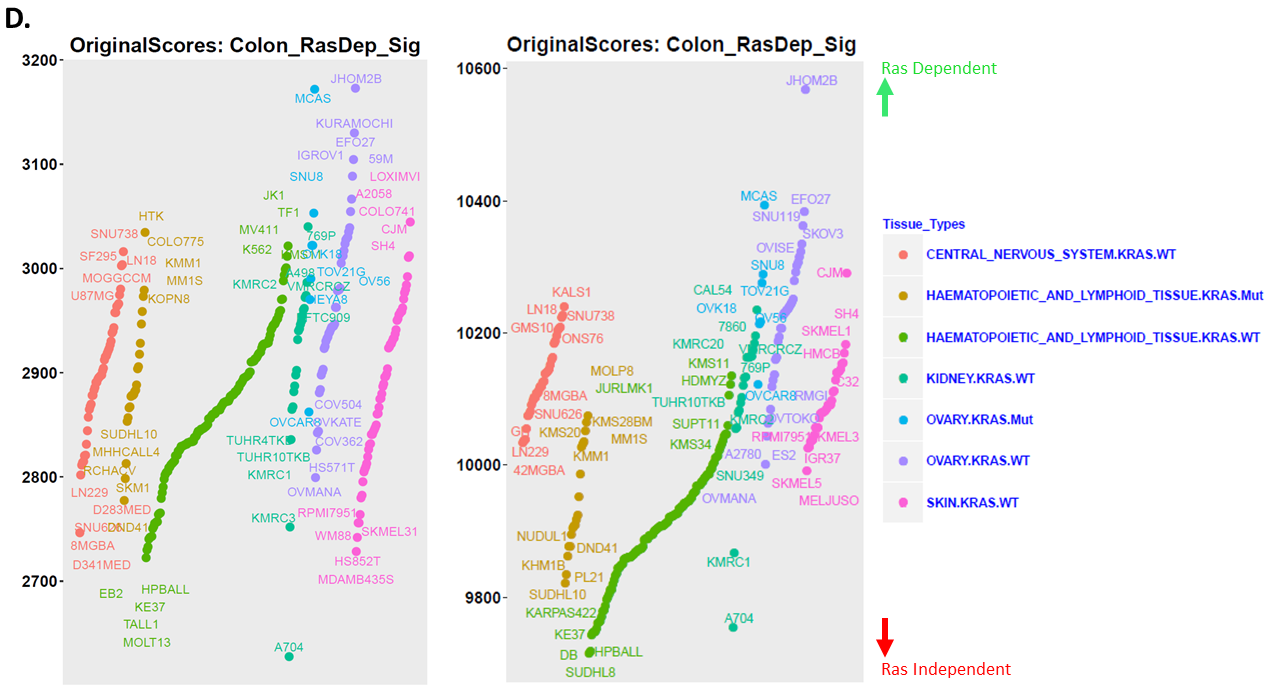
**

**Supplementary Figure 4**. Tissue types and KRAS mutation status do not obviously impact the ssGSEA scores distribution, which is relatively stable between microarray and RNAseq data especially for ssGSEA scores derived from the EMT signature. One cell line AU565 showing extreme data was taken out for plotting clarity for **A-D**, since it showed very different derived ssGSEA scores by colon Ras Dependent Signatures. In **A.** and **C.**, Union_of_Lines_SF1B_SF7: the cell lines that were measured initially for RDIs (RDI_SF1B and RDI_SF7, see Methods section) and have gene expression data, were plotted at the right side of plot (in pink and red), each of which were labeled starting with “*” in distinction from the original lines plotted for different tissue types. **A.** Distribution of ssGSEA scores derived from microarray (left) and RNASeq (right) data using EMT Ras Dependent Signature in cell lines from various tissue types with or without KRAS mutations including the three tissue types (Lung, Pancreas and Colon) that Ras genes play a central role in oncogenesis. **B.** Distribution of ssGSEA scores derived from microarray (left) and RNASeq (right) data using EMT Ras Dependent Signature in cell lines from additional tissue types with or without KRAS mutations. **C.** Distribution of ssGSEA scores derived from microarray (left) and RNASeq (right) data using colon Ras Dependent Signature in cell lines from various tissue types with or without KRAS mutations including the three tissue types (Lung, Pancreas and Colon) that Ras genes play a central role in oncogenesis; The ssGSEA scores derived from colon Ras Dependent Signatures seem having higher variations between the microarray and RNAseq platforms comparing EMT Ras Dependent Signature. KRAS mutation seem not significantly impact the ssGSEA score distribution. It seems there exist higher variations between the microarray and RNAseq platforms comparing with EMT Ras Dependent Signature. **D.** Distribution of ssGSEA scores derived from microarray (left) and RNASeq (right) data using colon Ras Dependent Signature in cell lines from additional tissue types with or without KRAS mutations. Red arrows and green arrows in all figures indicated the tendency and directions of Ras independent and Ras dependent respectively.


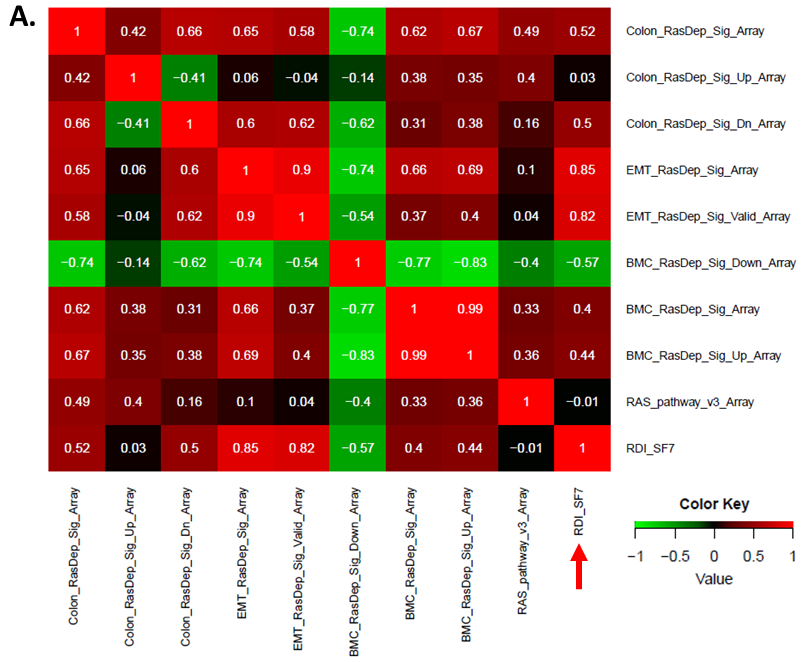


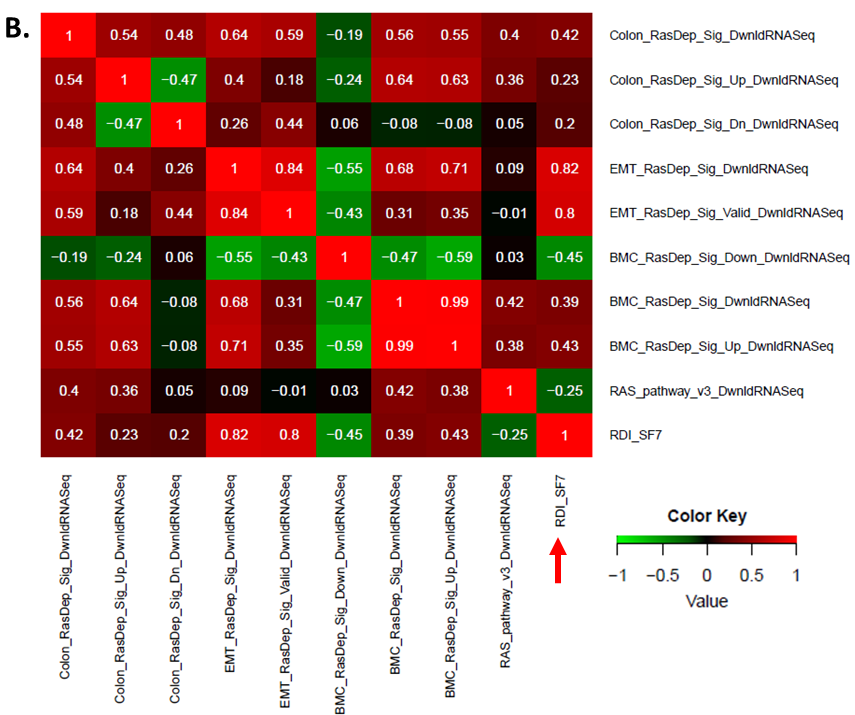


**
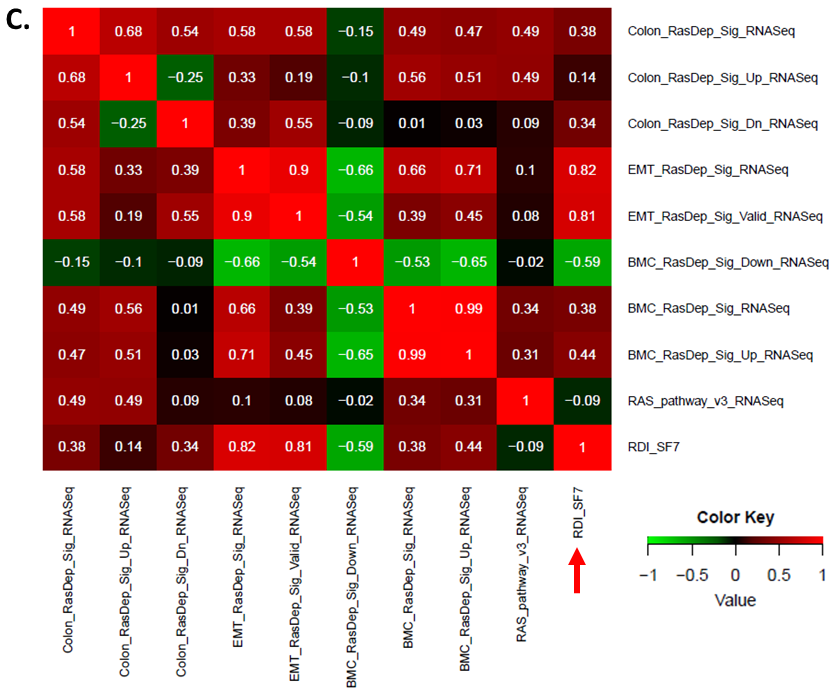
**

**Supplementary Figure 5.** Ras Dependency Indexes (RDIs) showed better correlation with ssGSEA scores of EMT signature comparing with many Ras-related signatures regardless of the data resources and platforms. Derived ssGSEA scores of EMT signatures showed better correlation with RDI_SF7 (red arrow, also see Methods section) comparing colon signatures, BMC Ras signatures, Ras pathway genes as well as other KEGG or Biocarta pathway genes (not show) regardless of the data resources and platforms. The gene signatures used for ssGSEA scores computation (described more details in Methods section) included: EMT_RasDep_Sig: EMT_RasDep_Sig_Valid, colon_RasDep_Sig, BMC_RasDep_Sig, RAS_pathway_v3. ssGSEA scores for all CCLE cell lines were computationally derived using corresponding gene signatures and expression data either of microarray or of RNASeq, and then standardized into z-scores across all of the CCLE lines. The correlation coefficients were computed pairwise for RDI_SF7 and gene-signature-based ssGSEA scores within cell lines that RDI_SF7 and expression data are both available. RNAseq data was either directly downloaded (RNAseqDwld) or processed in-house (RNAseq). **A.** Heatmap of correlation matrix for all data derived from CCLE array data and RDI_SF7 indicated by a red arrow. Values in cells of the heatmap are correlation coefficients. **B.** Heatmap of correlation matrix for all data derived from downloaded CCLE processed RNAseq data and RDI_SF7 indicated by a red arrow. Values in cells of the heatmap are correlation coefficients. **C.** Heatmap of correlation matrix for all data derived from in-house processed CCLE raw RNAseq data and RDI_SF7 indicated by a red arrow. Values in cells of the heatmap are correlation coefficients


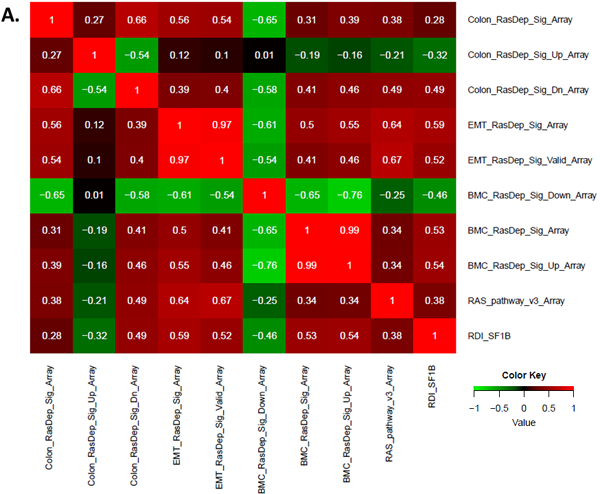


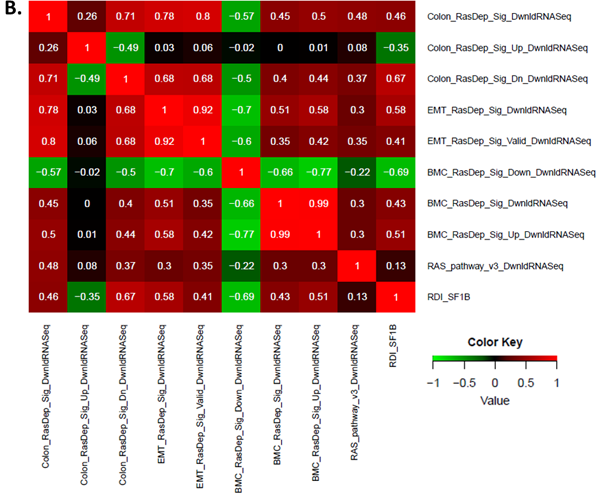


**
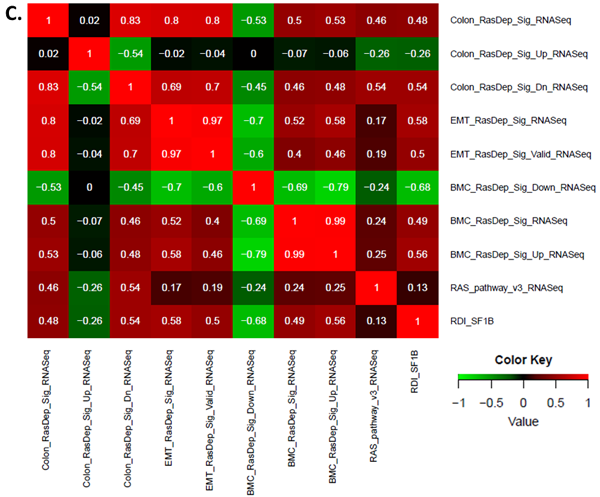
**

**
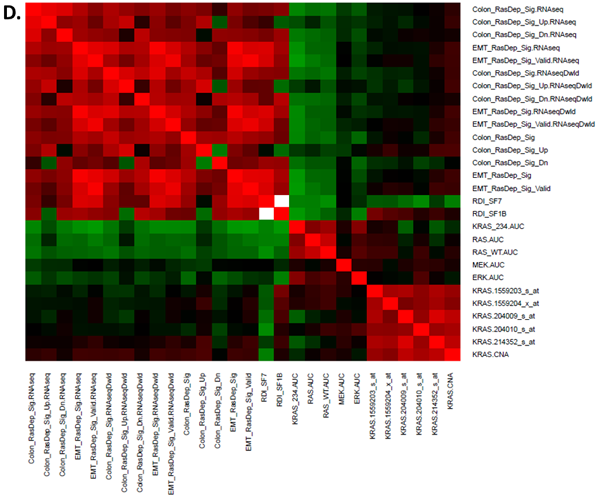
**

**Supplementary Figure 6.** Ras Dependency Indexes showed better correlation with ssGSEA scores of EMT signature comparing many Ras-related signatures regardless of the data resources and platforms. **A., B., C.:** Derived ssGSEA scores of EMT signatures showed better correlation with experimentally measured Ras Dependency Indexes (RDIs_SF1B, see Methods section) comparing colon signatures, EMT Ras dependent signature, BMC Ras signatures, Ras pathway genes as well as other KEGG or Biocarta pathway genes (data not show) regardless of the data resources and platforms. The correlation coefficients were computed pairwise for RDI_SF1B and gene-signature-based ssGSEA scores within cell lines that RDI_SF1B and expression data are both available. RNAseq data was either directly downloaded (RNAseqDwld) or processed in-house (RNAseq). **A.** Heatmap of correlation matrix for all data derived from CCLE array data and RDI_SF1B. Values in cells of the heatmap are correlation coefficients. **B.** Heatmap of correlation matrix for all data derived from downloaded CCLE processed RNAseq data and RDI_SF1B. Values in cells of the heatmap are correlation coefficients. **C.** Heatmap of correlation matrix for all data derived from self-processed CCLE raw RNAseq data and RDI_SF1B. Values in cells of the heatmap are correlation coefficients. **D**. Both Sets of Ras Dependency Indexes data (RDI_SF1B and RDI_SF7) show great correlation with EMT Ras dependent signature from both microarray and RNAseq platforms, although RD_SF7 showed better correlations. siREN data (RAS_AUC, RAS_WT_AUC, MEK_AUC, ERK_AUC; Yuan et al 2018) as well as KRAS expression (KRAS_XXX_X_at probeset) and copy number data (KRAS_CNA) were used as controls. White color indicated missing data.

| **Supplementary Table 1.** Differential gene lists of top 15 vs bottom 15 CCLE lines ranked by their ssGSEA scores of EMT signature in microarray data | | |
| --- | --- | --- |
| **Gene Lists** | **Number of Genes** |  |
| C_High_vs_C_Low_AdjP0_001 | 320 |  |
| C_High_vs_C_Low_AdjP0_01 | 759 |  |
| C_High_vs_C_Low_AdjP0_05 | 1632 |  |
| Hi_vs_Lo_AdjP0_001 | 2484 |  |
| Hi_vs_Lo_AdjP0_01 | 3229 |  |
| Hi_vs_Lo_AdjP0_05 | 3723 |  |
| L_High_vs_L_Low_AdjP0_001 | 2680 |  |
| L_High_vs_L_Low_AdjP0_01 | 4233 |  |
| L_High_vs_L_Low_AdjP0_05 | 5561 |  |
| P_High_vs_P_Low_AdjP0_001 | 194 |  |
| P_High_vs_P_Low_AdjP0_01 | 493 |  |
| P_High_vs_P_Low_AdjP0_05 | 1120 |  |
| **Supplementary Table 1.** Differential gene lists of top 15 vs bottom 15 CCLE lines ranked by their ssGSEA scores of EMT signature in microarray data from three tissues types or pooled lines. Expression data used are microarray data. C: Colon; P: Pancreas; L: Lung. High: top 15 cell lines with highest ssGSEA scores (Ras dependent lines); Low: bottom 15 cell lines with lowest ssGSEA scores (Ras-independent lines). Hi vs Lo: Pooled samples. adjP0_05,adjp0_01, adjp0_001: adjusted p-value <=0.05, 0.01,0.001, respectively. Each gene list is with cutoff of fold change at 1.5 fold for both directions of changing (up or down). Genes with multiple probesets are combined and unified for unique counting of genes. | |  |
|  |  |  |
|  |  |  |
|  |  |  |

| **Supplementary Table 2.** Differential gene lists of top 15 vs bottom 15 CCLE Lines ranked by their ssGSEA scores of EMT signature in RNAseq data | |
| --- | --- |
| **Gene Lists** | **Numbr of Genes** |
| C.High_vs_C.Low_DESeq2_adjP0_001 | 404 |
| C.High_vs_C.Low_DESeq2_adjP0_01 | 791 |
| C.High_vs_C.Low_DESeq2_adjP0_05 | 1466 |
| C.High_vs_C.Low_edgeR_FDR0_001 | 334 |
| C.High_vs_C.Low_edgeR_FDR0_01 | 793 |
| C.High_vs_C.Low_edgeR_FDR0_05 | 1591 |
| C.High_vs_C.Low_limmavoom_adjP0_001 | 152 |
| C.High_vs_C.Low_limmavoom_adjP0_01 | 456 |
| C.High_vs_C.Low_limmavoom_adjP0_05 | 991 |
| High_vs_Low_DESeq2_adjP0_001 | 2029 |
| High_vs_Low_DESeq2_adjP0_01 | 3156 |
| High_vs_Low_DESeq2_adjP0_05 | 4631 |
| High_vs_Low_edgeR_FDR0_001 | 2242 |
| High_vs_Low_edgeR_FDR0_01 | 3606 |
| High_vs_Low_edgeR_FDR0_05 | 5298 |
| High_vs_Low_limmavoom_adjP0_001 | 1930 |
| High_vs_Low_limmavoom_adjP0_01 | 3010 |
| High_vs_Low_limmavoom_adjP0_05 | 4380 |
| L.High_vs_L.Low_DESeq2_adjP0_001 | 1425 |
| L.High_vs_L.Low_DESeq2_adjP0_01 | 2363 |
| L.High_vs_L.Low_DESeq2_adjP0_05 | 3671 |
| L.High_vs_L.Low_edgeR_FDR0_001 | 1370 |
| L.High_vs_L.Low_edgeR_FDR0_01 | 2461 |
| L.High_vs_L.Low_edgeR_FDR0_05 | 3957 |
| L.High_vs_L.Low_limmavoom_adjP0_001 | 1157 |
| L.High_vs_L.Low_limmavoom_adjP0_01 | 2322 |
| L.High_vs_L.Low_limmavoom_adjP0_05 | 3870 |
| P.High_vs_P.Low_DESeq2_adjP0_001 | 138 |
| P.High_vs_P.Low_DESeq2_adjP0_01 | 358 |
| P.High_vs_P.Low_DESeq2_adjP0_05 | 818 |
| P.High_vs_P.Low_edgeR_FDR0_001 | 79 |
| P.High_vs_P.Low_edgeR_FDR0_01 | 304 |
| P.High_vs_P.Low_edgeR_FDR0_05 | 856 |
| P.High_vs_P.Low_limmavoom_adjP0_001 | 210 |
| P.High_vs_P.Low_limmavoom_adjP0_01 | 523 |
| P.High_vs_P.Low_limmavoom_adjP0_05 | 1129 |

**Supplementary Table 2.** Differential gene lists of top 15 vs bottom 15 CCLE Lines ranked by their ssGSEA scores of EMT signature from three tissues types or pooled lines. Expression data used are RNAseq data and analyzed by three methods: limmavoom, edgeR and DESeq2. C: Colon; P: Pancreas; L: Lung. High: top 15 cell lines with highest ssGSEA scores (Ras dependent lines); Low: bottom 15 cell lines with lowest ssGSEA scores (Ras-independent lines). adjP0_05,adjp0_01, adjp0_001: adjusted p-value <=0.05, 0.01,0.001, respectively;FDR0_05, FDR0_01, FDR0_001: FDR <=0.05, 0.01,0.001, respectively.


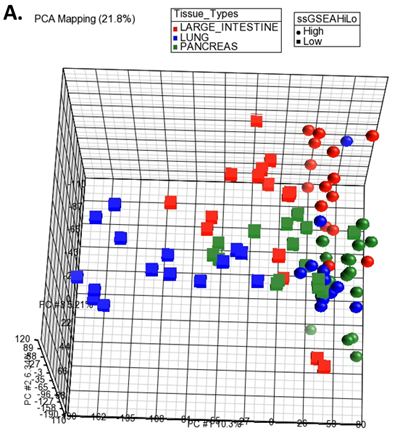


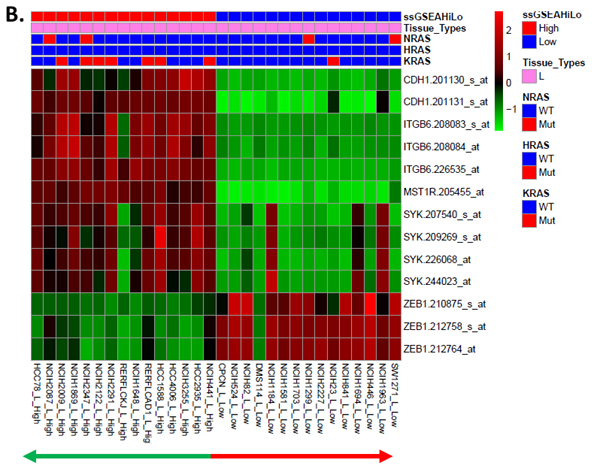


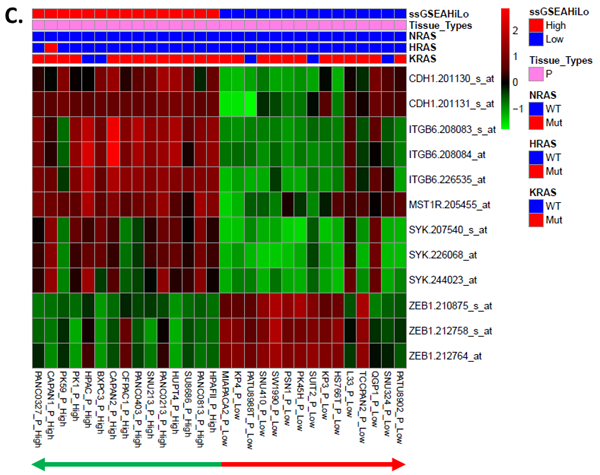


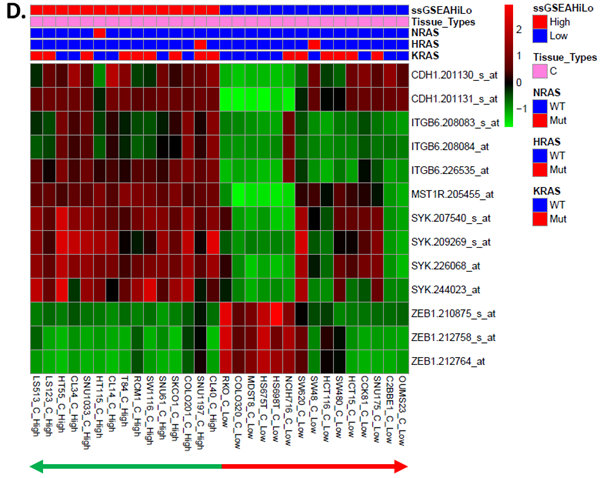


**Supplementary Figure 7.** Expression analysis of microarray data from top 15 vs bottom 15 CCLE cell lines with highest vs lowest ssGSEA scores in three tissue types: lung, pancreas and colon or large intestine. **A.** PCA plot. **B.,C.,D.:** The five critical signature genes from the short Validated EMT Signature behaved the same or similar as original Settleman’s Paper (Singh et al 2009) in microarray data of top 15 vs bottom 15 cell lines with highest vs lowest ssGSEA scores in three tissues types: lung, pancreas, and colon **B.** lung cell lines. **C.** pancreas cell lines. **D.** colon cell lines. Alias of genes: ZEB1=TCF8. Green arrows: Ras-dependent cell lines; Red arrows: Ras-independent cell lines.

**Supplementary Table 3.** SYK and PAK1 are critical genes shared by EMT signature and Ras Independent Pathway in NK cells.

**Supplementary Table 3.** SYK and PAK1 are critical genes shared by EMT signature and Ras Independent Pathway in NK cells. The diagonal line (left top to right bottom) has the actual number of genes for each gene signature in bold. The right upper part of the diagonal line are p-values of enrichment level assessed by Fisher’s exact test; Enrichment p-values were derived from Fisher’s exact tests on the levels of overlap between each pair of gene signature lists in table using genes from the CCLE microarray data with valid signals as background total number of genes in 2X2 contingency table. The lower left part of the diagonal line are number of genes shared by corresponding two genes signatures followed by the names of shared actual genes with “||” if the number of shared genes is not too large. Colored cells are pairs of gene signatures with significant overlap in their shared genes assessed by Fisher’s exact test. The cells with the same color showed p-values and numbers of shared genes between each corresponding pair of gene signatures in right upper part or lower left part respectively. There is no significant overlap with EMT signature and Ras Independent Pathway in NK cells. Only two genes SYK and PAK1 as the potential critical genes shared by EMT signature and Ras Independent Pathway in NK cells. SYK and PAK1 are highlighted in red, while KRAS highlighted in orange.

**
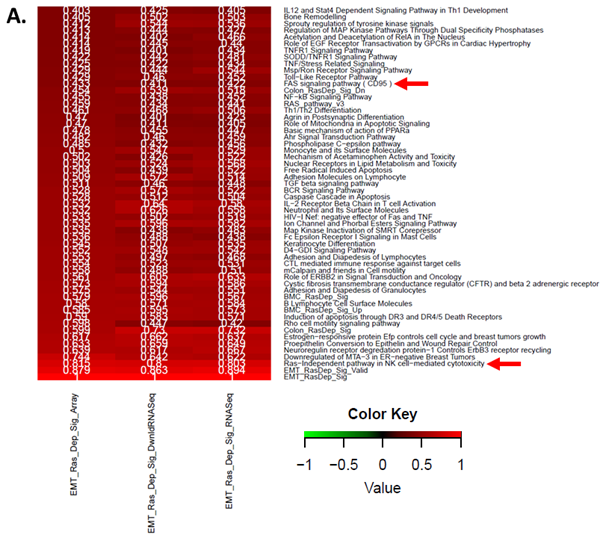
**

**
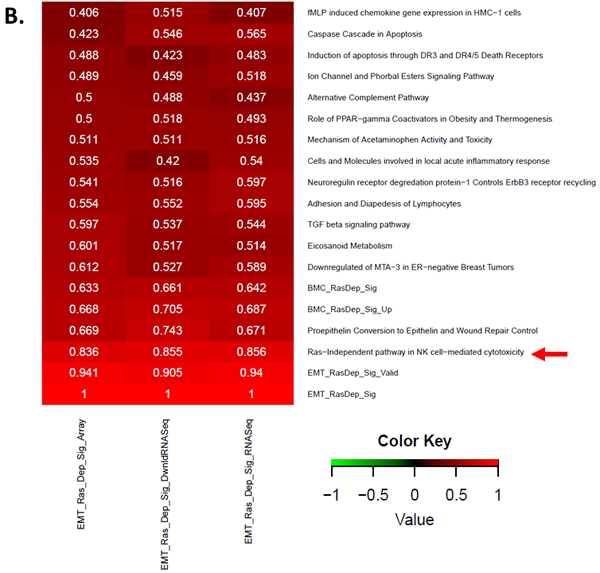
**

**
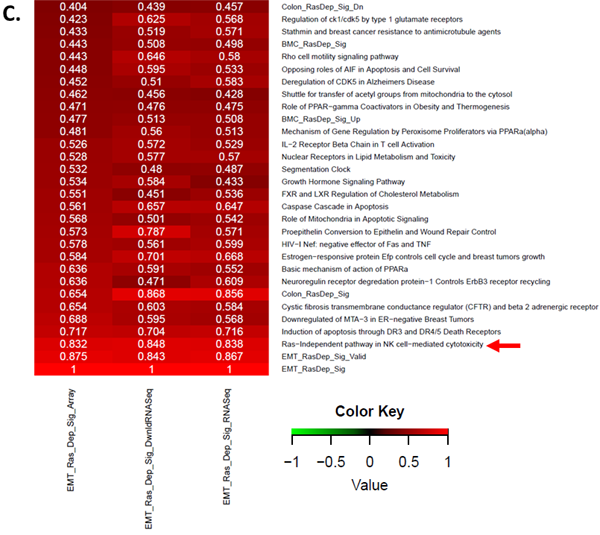
**

**
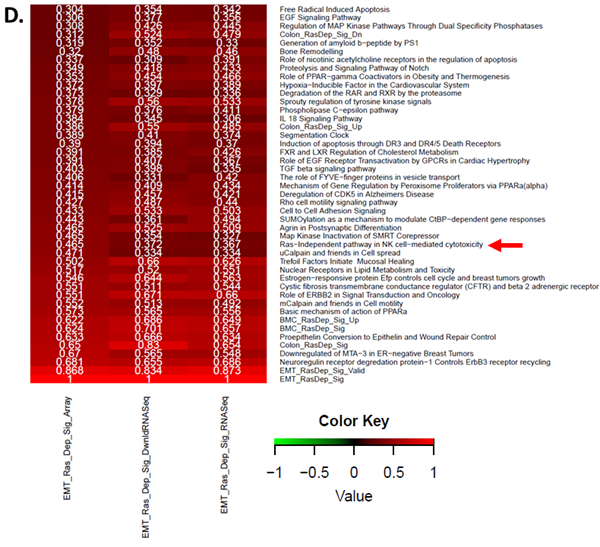
**

**Supplementary Figure 8.** Correlation heatmap between EMT_RasDep_Sig gene signature and individual Biocarta pathways as gene signatures for their corresponding derived ssGSEA scores. Data used are CCLE microarray (EMT_Ras_Dep_Sig_Array), or RNAseq data either processed data downloaded from CCLE (EMT_Ras_Dep_Sig_DwnldRNASeq) or processed in-house (EMT_Ras_Dep_Sig_RNASeq). **A**. Amongst CCLE lung cell lines; **B**. Amongst CCLE pancreas cell lines; **C**. Amongst CCLE colon cell lines. **D**. Amongst all CCLE cell lines. Red arrows indicated the Biocarta pathways that were uncovered from DEGs and PPEP analysis of top vs bottom ssGSEA samples from Figure 3B and 3C. Ras-Independent pathway in NK cell-mediated cytotoxicity are common and at the top as the most correlated pathway in all 3 types of cell lines, only Fas signaling pathway (CD95) is shown up in lung cell lines, but not in pancreas and colon cell lines, consistent with DEGs analysis results of high vs low ssGSEA samples.

**
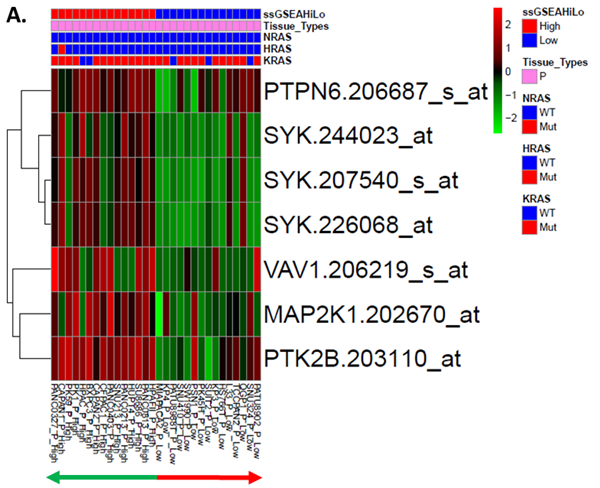
**

**
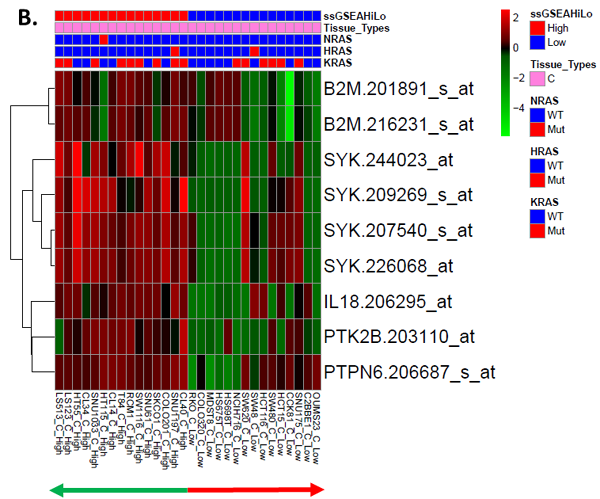
**

**Supplementary Figure 9**. Differential expressed genes from CCLE microarray data

showed coordinate and consensus down-regulated expression pattern in Ras-independent cell lines of different tissue types: pancreas and colon in a Biocarta pathway: Ras-Independent pathway in NK cell-mediated cytotoxicity (Ras_Ind NK Pathway). **A**. Heatmap of the DEGs in Ras_Ind NK Pathway between top 15 vs bottom 15 CCLE pancreas cell lines with highest vs lowest ssGSEA scores. DEGs are from P_High_vs_P_Low_AdjP0_05FC1_5 (detailed in Supplementary Table 1). **B**. Heatmap of the DEGs in Ras_Ind NK Pathway between top 15 vs bottom 15 CCLE colon cell lines with highest vs lowest ssGSEA scores. DEGs are from C_High_vs_C_Low_AdjP0_05FC1_5 (detailed in Supplementary Table 1). Green arrows: Ras-dependent cell lines; Red arrows: Ras-independent cell lines.


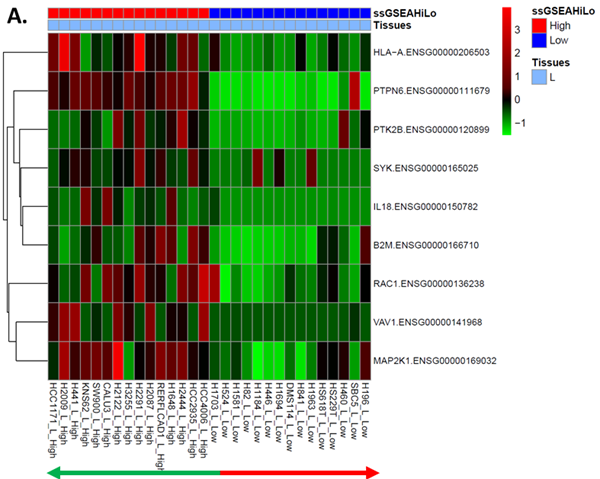


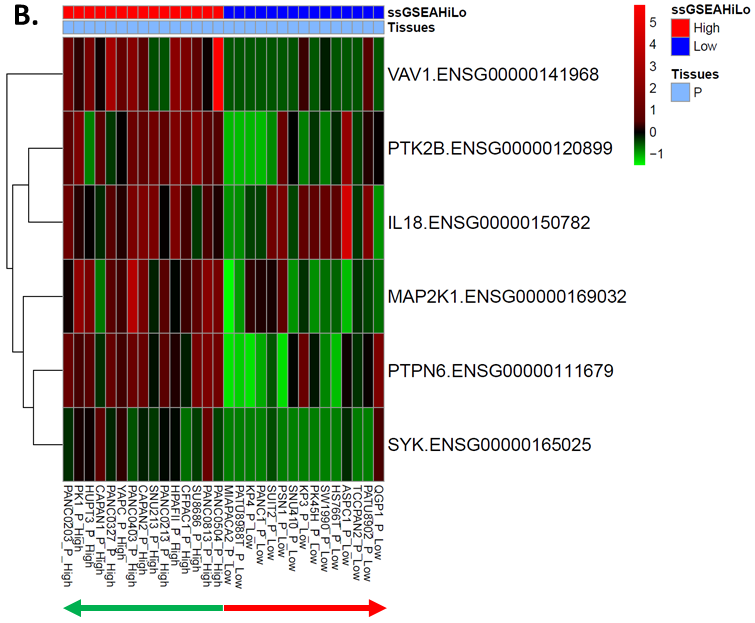


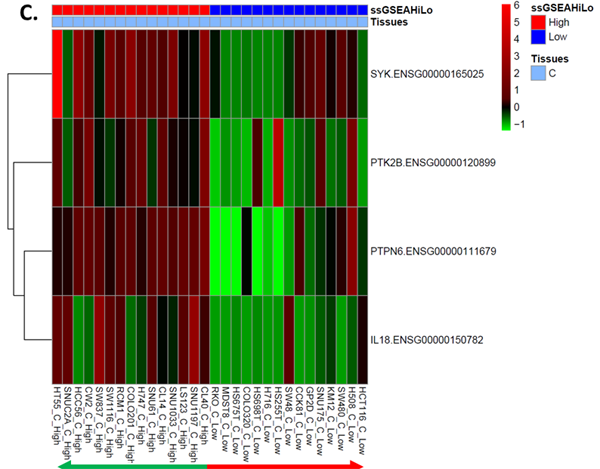


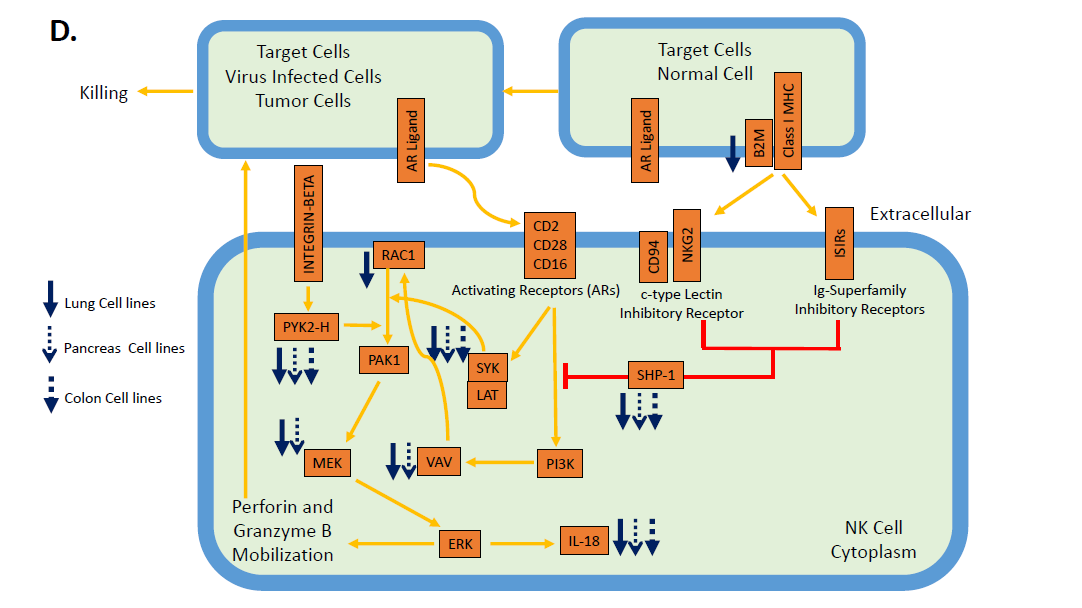


**Supplementary Figure 10**. Differential expressed genes from CCLE RNAseq data showed coordinate and consensus down-regulated expression pattern in Ras-independent cell lines of three different tissue types: lung, pancreas and colon in a Biocarta pathway: Ras-Independent pathway in NK cell-mediated cytotoxicity (Ras_Ind NK Pathway). **A.** Heatmap of the differential expressed genes (DEGs) in Ras_Ind NK Pathway between top 15 vs bottom 15 CCLE lung cell lines with highest vs lowest ssGSEA scores. DEGs are from L.High_vs_L.Low_limmavoom_adjP0_05 (detailed in Supplementary Table 2). **B.** Heatmap of the DEGs in Ras_Ind NK Pathway between top 15 vs bottom 15 CCLE pancreas cell lines with highest vs lowest ssGSEA scores. DEGs are from

P.High_vs_P.Low_limmavoom_adjP0_05 (detailed in Supplementary Table 2). **C.** Heatmap of the DEGs in Ras_Ind NK Pathway between top 15 vs bottom 15 CCLE colon cell lines with highest vs lowest ssGSEA scores. DEGs are from C.High_vs_C.Low_limmavoom_adjP0_05 (detailed in Supplementary Table 2). **D.** Pathway overlay of DEGs from all three tissue types (A,B,C) on Ras_Ind NK Pathway. High ssGSEA scores cell lines are more like Ras-dependent lines whereas low ssGSEA score cell lines are more like Ras-independent lines. Blue arrows showed down-regulated of corresponding genes in RNAseq data of CCLE cell lines with low ssGSEA scores (Ras independent lines) for cell lines of different tissue origins. Gene alias as followed: MAP2K1=MEK; B2M=Beta-2-macroglobulin associated with MHC; PTK2B=PYK2; PTPN6=SHP-1; Expression data used are CCLE RNAseq data and DEGs are derived using limma-voom approach (Law et al 2014). Green arrows: Ras-dependent cell lines; Red arrows: Ras-independent cell lines.


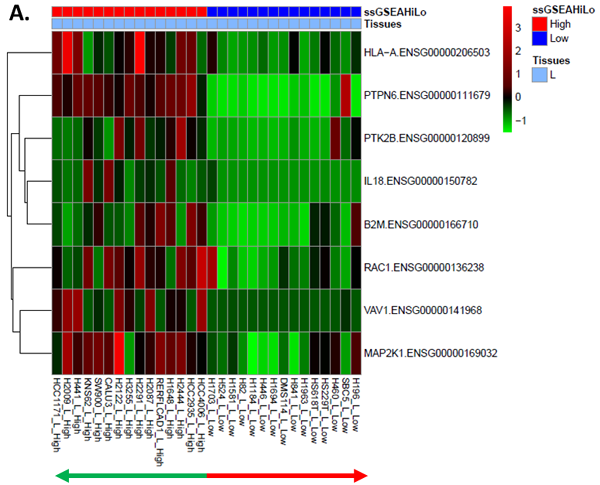


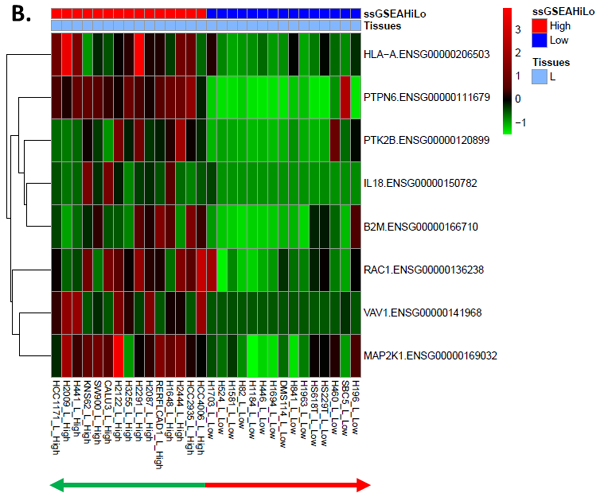


**Supplementary Figure 11.** Differential expressed genes from CCLE RNAseq data showed coordinate and consensus down-regulated expression pattern in Ras-independent cell lines of lung in a Biocarta pathway: Ras-Independent pathway in NK cell-mediated cytotoxicity (Ras_Ind NK Pathway) validated by two other analysis methods besides limma-voom method (Supplementary Figure 10). **A**. Heatmap of the differential expressed genes (DEGs) in Ras_Ind NK Pathway between top 15 vs bottom 15 CCLE lung cell lines with highest vs lowest ssGSEA scores. DEGs are from L.High_vs_L.Low_edgeR_FDR0_05 (detailed in Supplementary Table 2). **B.** Heatmap of the DEGs in Ras_Ind NK Pathway between top 15 vs bottom 15 CCLE pancreas cell lines with highest vs lowest ssGSEA scores. DEGs are from L.High_vs_L.Low_DESeq2_adjP0_05 (detailed in Supplementary Table 2). High ssGSEA scores cell lines are more like Ras-dependent lines whereas low ssGSEA score cell lines are more like Ras-independent lines. Gene alias as followed: MAP2K1=MEK; B2M=Beta-2-macroglobulin associated with MHC; PTK2B=PYK2; PTPN6=SHP-1; Expression data used are CCLE RNAseq data and DEGs are derived using edgeR and DESeq2 approaches respectively. Green arrows: Ras-dependent cell lines; Red arrows: Ras-independent cell lines.


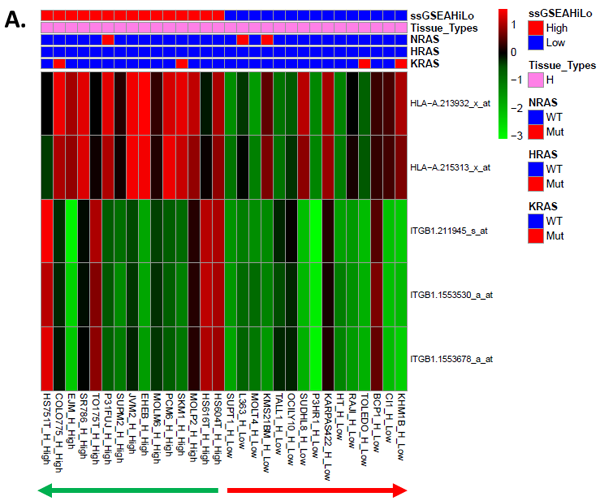


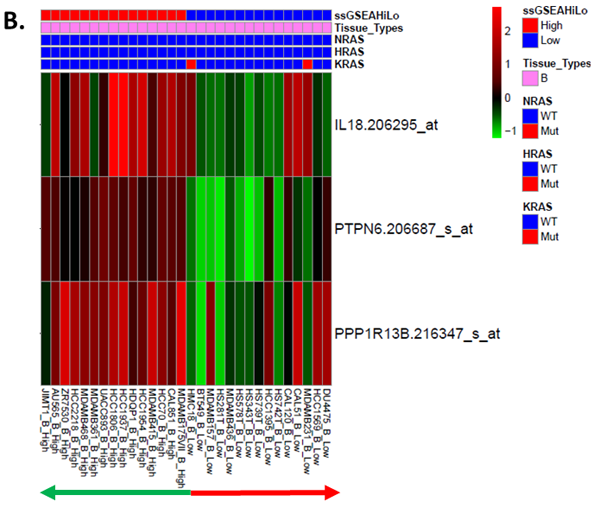


**Supplementary Figure 12**. Differential expressed genes from CCLE microarray data showed coordinate and consensus down-regulated expression pattern in Ras-independent cell lines of three different tissue types: haematopoietic_and_lymphoid, breast, and skin, in a Biocarta pathway: Ras-Independent pathway in NK cell-mediated cytotoxicity (Ras_Ind NK Pathway). **A**. Heatmap of the differential expressed genes (DEGs) in Ras_Ind NK Pathway between top 15 vs bottom 15 CCLE haematopoietic_and_lymphoid cell lines with highest vs lowest ssGSEA scores. Total 2 DEGs out of total 17 genes in this pathway (one gene has two probesets and the other has 3 probesets); H: Haematopoietic_and_Lymphoid Cell Lines. **B**. Heatmap of the DEGs in Ras_Ind NK Pathway between top 15 vs bottom 15 CCLE breast cell lines with highest vs lowest ssGSEA scores. The same DEGs cutoff was applied similar to Figure 4. B: Breast Cell Lines. Only 3 DEGs out of total 17 genes in this pathway for breast result. No single DEGs for skin result (no plot to show). DEGs were derived at adjusted p-value <=0.05 and FC>=1.5. Gene alias as followed: MAP2K1=MEK; B2M=Beta-2-macroglobulin associated w/ MHC; PTK2B=PYK2; PTPN6=SHP-1. Green arrows: Ras-dependent cell lines; Red arrows: Ras-independent cell lines.

**
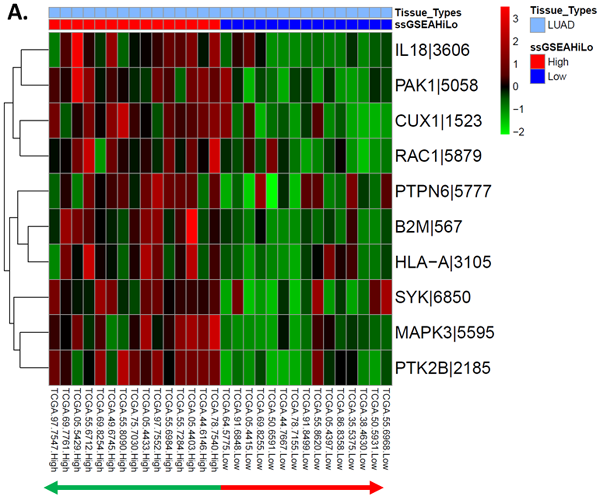
**

**
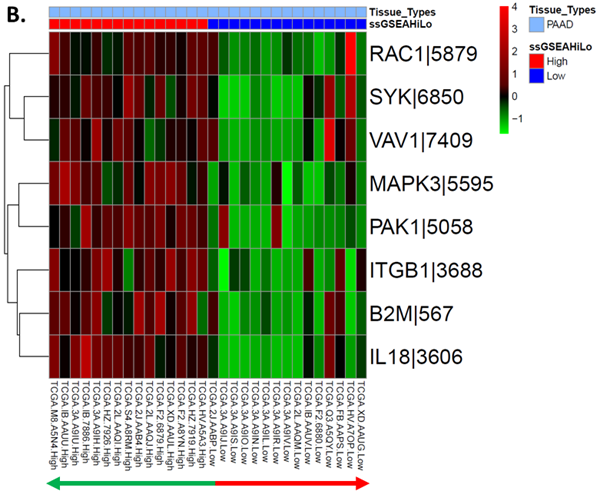
**

**
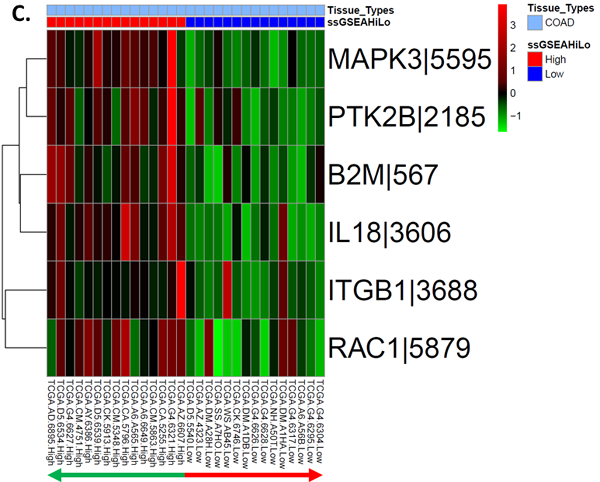
**


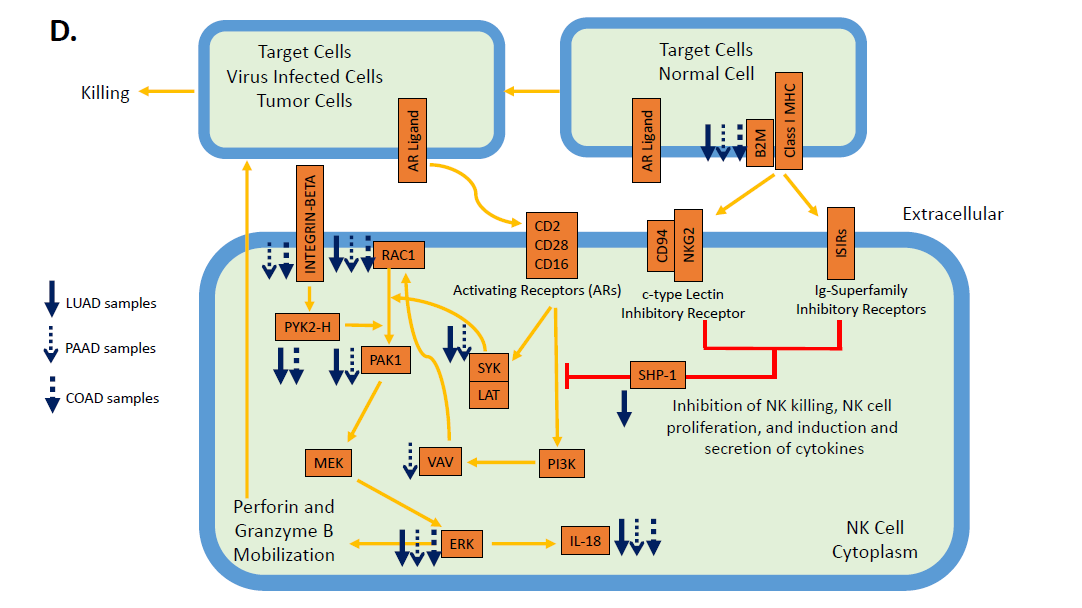


**Supplementary Figure 13**. Differential expressed genes from TCGA RNAseq data showed coordinate and consensus down-regulated expression pattern in Ras-independent patient samples of three different tumor types: LUAD(lung), PAAD(pancreas) and COAD(colon) in a Biocarta pathway: Ras-Independent pathway in NK cell-mediated cytotoxicity (Ras_Ind NK Pathway). **A.** Heatmap of the differential expressed genes (DEGs) in Ras_Ind NK Pathway between top 15 vs bottom 15 TCGA LUAD samples with highest vs lowest ssGSEA scores. **B.** Heatmap of the DEGs in Ras_Ind NK Pathway between top 15 vs bottom 15 TCGA PAAD samples with highest vs lowest ssGSEA scores. **C.** Heatmap of the DEGs in Ras_Ind NK Pathway between top 15 vs bottom 15 TCGA COAD samples with highest vs lowest ssGSEA scores. **D.** Pathway overlay of DEGs from all three tissue types (**A,B,C**) on Ras_Ind NK Pathway. High ssGSEA scores cell lines are more like Ras-dependent samples whereas low ssGSEA score cell lines are more like Ras-independent samples. Blue arrows showed down-regulated of corresponding genes in RNAseq data of TCGA patient samples with low ssGSEA scores (Ras independent samples) for patients of different tumor types. Gene alias as followed: MAP2K1=MEK; B2M=Beta-2-macroglobulin associated with MHC; PTK2B=PYK2; PTPN6=SHP-1; Expression data used was TCGA RNAseq data and DEGs were derived using limma-voom method at adjusted p-value<=0_05 (**A.,B.,C.)**. Green arrows: Ras-dependent cell lines; Red arrows: Ras-independent cell lines.


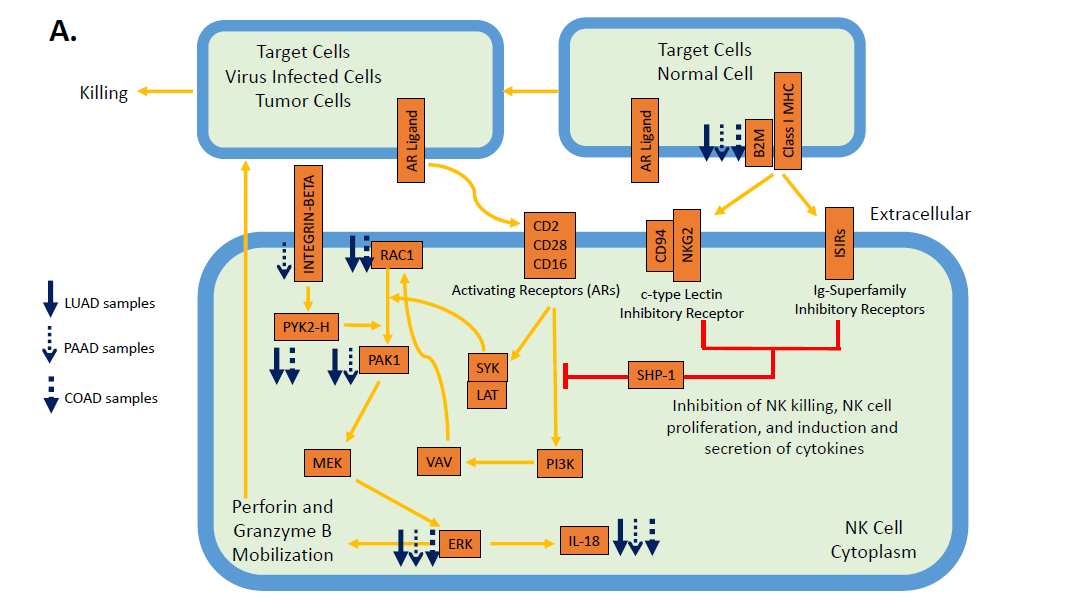


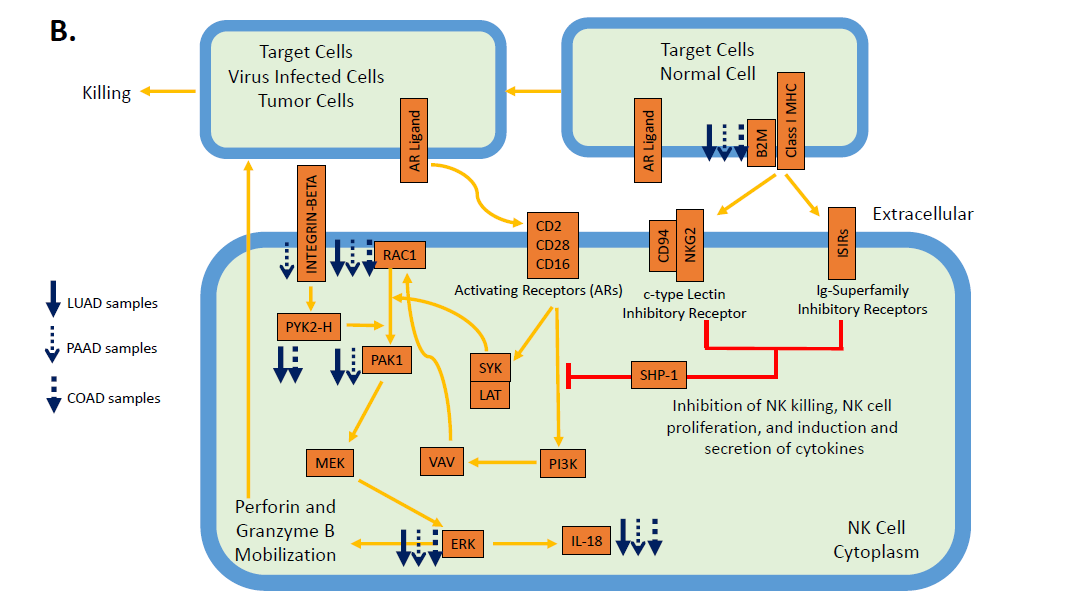


**Supplementary Figure 14**. Differential expressed genes from TCGA RNAseq data showed coordinate and consensus down-regulated expression pattern in Ras-independent patient samples of three different tumor types: LUAD(lung), PAAD(pancreas) and COAD(colon) in a Biocarta pathway: Ras-Independent pathway in NK cell-mediated cytotoxicity (Ras_Ind NK Pathway) validated by two other analysis methods besides limma-voom method (Supplementary Figure 13). **A.** Pathway overlay of DEGs between top 15 vs bottom 15 TCGA patient samples with highest vs lowest ssGSEA scores from TCGA RNAseq data from all three tissue types analyzed by edgeR method. **B.** Pathway overlay of DEGs between top 15 vs bottom 15 TCGA patient samples with highest vs lowest ssGSEA scores from TCGA RNAseq data from all three tissue types analyzed by DESeq2 method. High ssGSEA scores cell lines are more like Ras-dependent samples whereas low ssGSEA score cell lines are more like Ras-independent samples. Blue arrows showed down-regulated of corresponding genes in RNAseq data of TCGA patient samples with low ssGSEA scores (Ras independent samples) for patients of different tumor types. Gene alias as followed: MAP2K1=MEK; B2M=Beta-2-macroglobulin associated with MHC; PTK2B=PYK2; PTPN6=SHP-1; Expression data used are TCGA RNAseq data and DEGs are derived using edgeR and DESeq2 approaches.


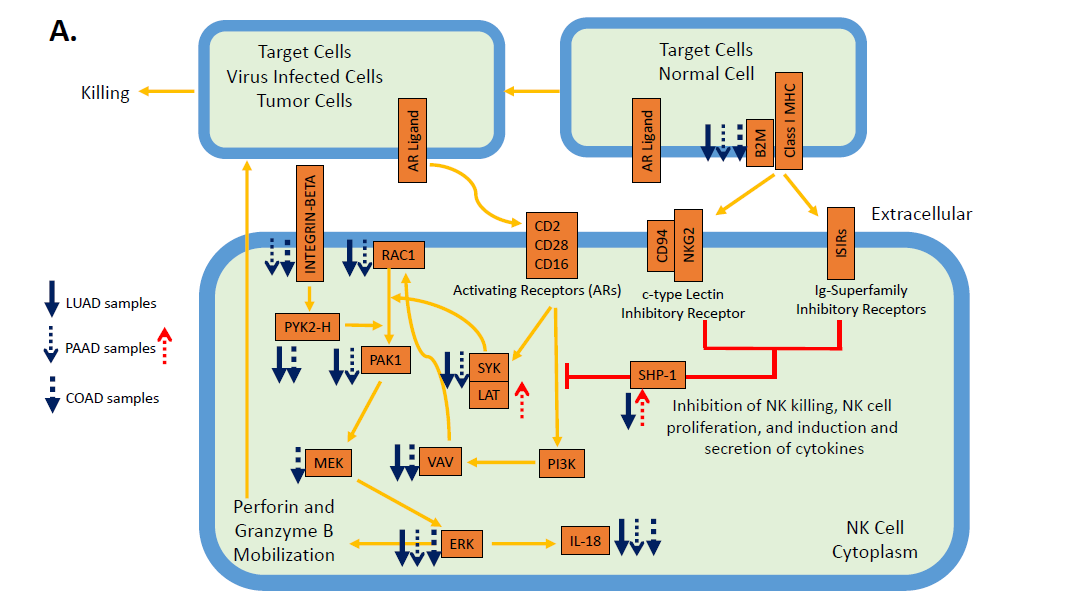


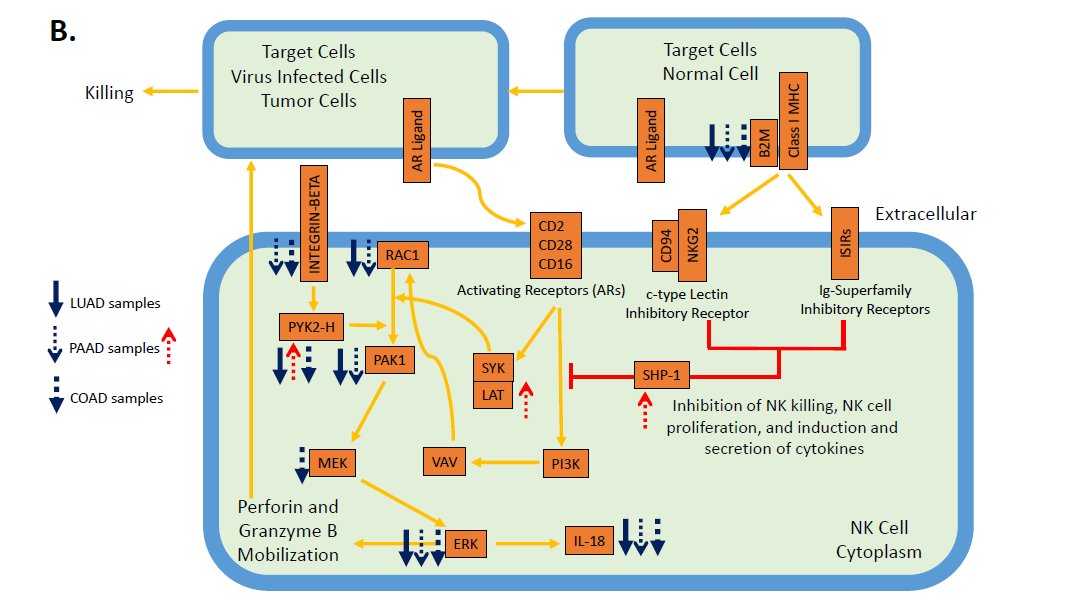


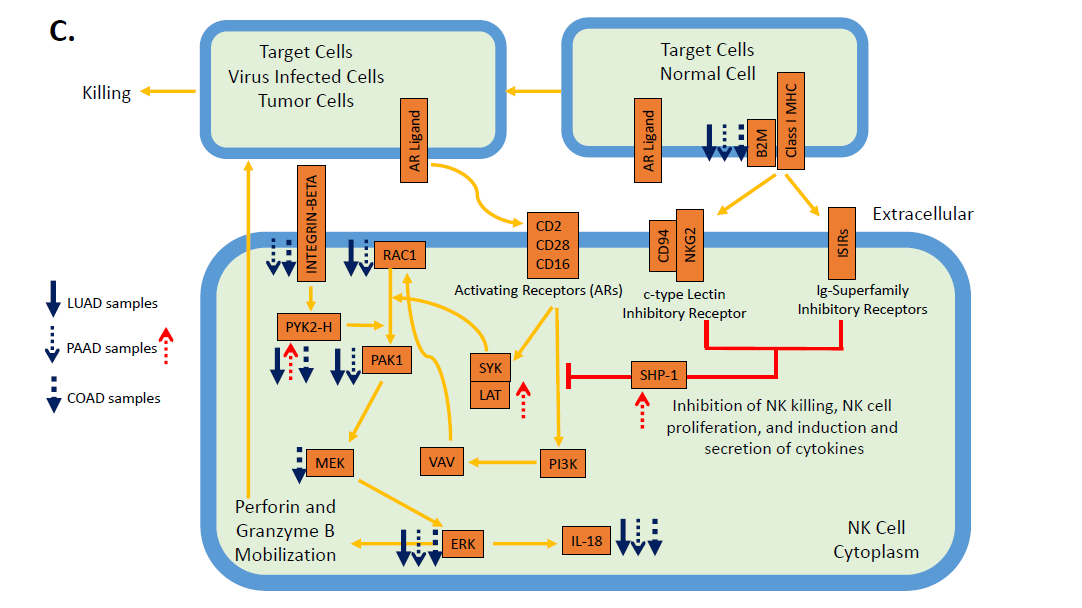


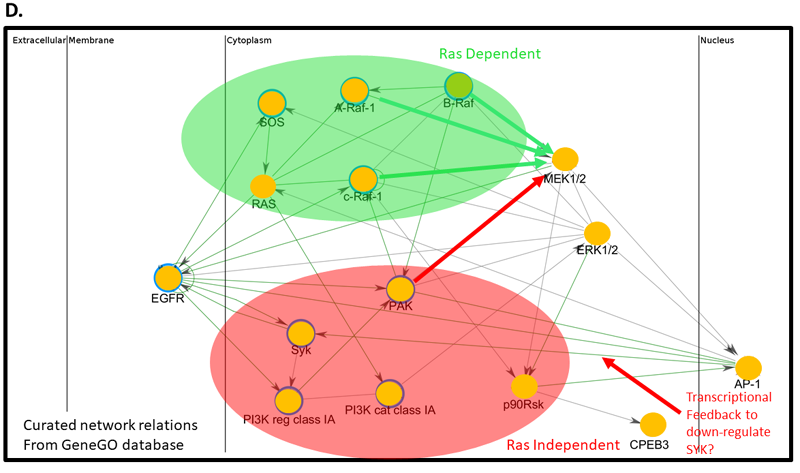


**Supplementary Figure 15**. Accumulated evidence led to postulated potential global presence of both Ras-dependent and Ras-independent cascades that lead to context-dependent activation of downstream MAPK signaling pathway. **A.** Overlay of DEGs between top 30 vs bottom 30 TCGA patient samples with highest vs lowest ssGSEA scores from TCGA RNAseq data from all three tissue types analyzed by limma-voom method. **B.** Overlay of DEGs between top 30 vs bottom 30 TCGA patient samples with highest vs lowest ssGSEA scores from TCGA RNAseq data from all three tissue types analyzed by edgeR method. **C.** Overlay of DEGs between top 30 vs bottom 30 TCGA patient samples with highest vs lowest ssGSEA scores from TCGA RNAseq data from all three tissue types analyzed by DESeq2 method. **A.**, **B.**, **C.**: Differential expressed genes from TCGA RNAseq data showed primarily coordinate and consensus down-regulated expression pattern in Ras-independent patient samples of three different tumor types: LUAD(lung), PAAD(pancreas) and COAD(colon) in a Biocarta pathway: Ras-Independent pathway in NK cell-mediated cytotoxicity (Ras_Ind NK Pathway) regardless of the DEGs analysis methods and numbers of top and bottom ssGSEA score samples chosen. High ssGSEA scores cell lines are more like Ras-dependent samples whereas low ssGSEA score cell lines are more like Ras-independent samples. Blue arrows showed down-regulated of corresponding genes in RNAseq data of TCGA patient samples with low ssGSEA scores (Ras independent samples) for patients of different tumor types, whereas red arrows for few genes up-regulated in samples with low ssGSEA scores. Gene alias as followed: MAP2K1=MEK; B2M=Beta-2-macroglobulin associated with MHC; PTK2B=PYK2; PTPN6=SHP-1; Expression data used are TCGA RNAseq data and DEGs are derived using edgeR and DESeq2 approaches. **D.** The network view showing potential global presence of both Ras-dependent and Ras-independent cascades that lead to context-dependent activation of downstream MAPK signaling pathway. Network relations were retrieved for relevant genes from curation databases of GeneGO ([https://portal.genego.com/cgi/data_manager.cgi#](https://portal.genego.com/cgi/data_manager.cgi)) using knowledge from protein-protein interaction database, canonical pathways, literature mining. There exist two signaling cascades highlighted either as conventional Ras-dependent context at the top (green oval) or Ras-independent context at the bottom (red oval) involving SYK, PAK1 and PI3K converged into MEK1/2 for downstream MAPK signaling pathway. The RSK gene, a critical gene/node in siREN study^14^, cross-talks with many components of this network. Also, SYK could be modulated by transcriptional feedback from downstream transcription factor that may explain why we saw SYK downregulated in our data.

**Supplementary Table 4.** RAC1-related differential gene lists of top 15 vs bottom 15 cancer patient samples ranked by their ssGSEA scores of EMT signature in three TCGA tumor types: COAD, LUAD, or PAAD.

**Supplementary Table 4.** RAC1-related differential gene lists (DEGs) of top 15 vs bottom 15 cancer patient samples ranked by their ssGSEA scores of EMT signature in three TCGA tumor type: COAD, LUAD, or PAAD. Expression data used are RNAseq data. For each tumor type, DEGs are derived between the top 15 patient samples with highest ssGSEA scores (Ras dependent) and the bottom 15 patient samples with lowest ssGSEA scores (Ras-independent) using three analysis methods: DESeq2,edgeR, and limma-voom. Shown in table are log2FC (log2 of fold change) at adjusted p-value <=0.05 or FDR <=0.05. NotDEG: not a DEG to meet the criteria of adjusted p-value <=0.05 or FDR <=0.05.

**Supplementary Table 5.** RAC1-related differential gene lists of top 30 vs bottom 30 cancer patient samples ranked by their ssGSEA scores of EMT signature in three TCGA tumor types: COAD, LUAD, or PAAD.

**Supplementary Table 5.** RAC1-related differential gene lists(DEGs) of top 30 vs bottom 30 cancer patient samples ranked by their ssGSEA scores of EMT signature in three TCGA tumor types: COAD, LUAD, or PAAD. Expression data used are RNAseq data. For each tumor type, DEGs are derived between top 30 patient samples with highest ssGSEA scores (Ras dependent) and the bottom 30 patient samples with lowest ssGSEA scores (Ras-independent) using three analysis methods: DESeq2, edgeR, and limma-voom. Shown in table are log2FC (log2 of fold change) at adjusted p-value <=0.05 or FDR <=0.05. NotDEG: not a DEG to meet the criteria of adjusted p-value <=0.05 or FDR <=0.05.

**Supplementary Table 6.** Opposite expression patterns of PREX1 and RAC1 genes in top 15 vs bottom 15 cancer patient samples ranked by their ssGSEA scores of EMT signature in three TCGA tumor types: COAD, LUAD, or PAAD.

**Supplementary Table 6.** Opposite expression patterns of PREX1 and RAC1 genes in top 15 vs bottom 15 cancer patient samples ranked by their ssGSEA scores of EMT signature in three TCGA tumor types: COAD, LUAD, or PAAD. Expression data used are RNAseq data. For each tumor type, DEGs are derived between the top 15 patient samples with highest ssGSEA scores (Ras dependent) and the bottom 15 patient samples with lowest ssGSEA scores (Ras-independent) using analysis methods: DESeq2, Shown in table are logFC (log2 of fold change) and adjP (adjusted p-value) for selected genes. Similar trends for opposite expression patterns of PREX1 and RAC1 (and RAC2 as well) were shown by their logFCs despite of insignificant adjusted p-values in some cases,

**Supplementary Table 7.** RAC1-related differential gene lists of top 15 vs bottom 15 CCLE cell lines ranked by their ssGSEA scores of EMT signature from three tissue types: colon, lung, or pancreas.

**Supplementary Table 7.** RAC1-related differential gene lists (DEGs) of top 15 vs bottom 15 CCLE cell lines ranked by their ssGSEA scores of EMT signature from three tissue types: colon, lung, or pancreas. Expression data used are RNAseq data. For each tissue type, DEGs are derived between top 15 cell lines with highest ssGSEA scores (Ras dependent) and the bottom 15 cell lines with lowest ssGSEA scores (Ras-independent) using three analysis methods: DESeq2, edgeR, and limma-voom. Shown in table are log2FC (log2 of fold change) at adjusted p-value <=0.05 or FDR <=0.05. NotDEG: not a DEG to meet the criteria of adjusted p-value <=0.05 or FDR <=0.05.

**Supplementary Table 8.** RAC1-related differential gene lists of top 15 vs bottom 15 CCLE cell lines ranked by their ssGSEA scores of EMT signature from three tissue types: colon, lung, or pancreas.

**Supplementary Table 8.** RAC1-related differential gene lists (DEGs) of top 15 vs bottom 15 CCLE cell lines ranked by their ssGSEA scores of EMT signature from three tissue types: colon, lung, or pancreas. Expression data used are microarray data. For each tissue type, DEGs are derived between the top 15 cell lines with highest ssGSEA scores (Ras dependent) and the bottom 15 cell lines with lowest ssGSEA scores (Ras-independent). Shown in table are log2FC (log2 of fold change) at adjusted p-value <=0.05 or FDR <=0.05. NotDEG: not a DEG to meet the criteria of adjusted p-value <=0.05 or FDR <=0.05. Each column is a combination of a gene with one of its probesets (separated by “.”) due to many genes have multiple probesets in the microarray. Due to limited space, PAK2 and PAK3 data not shown since they are all “NotDEG”


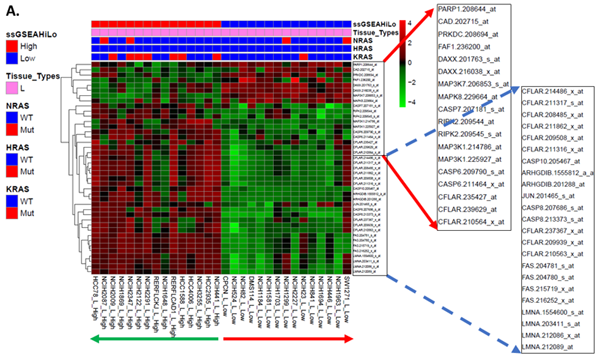


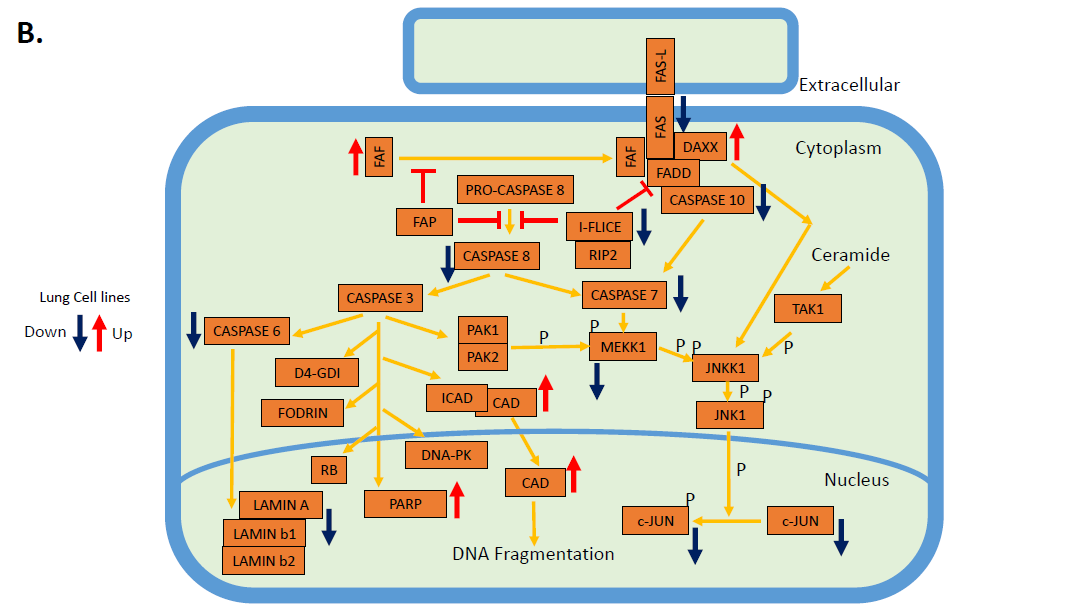


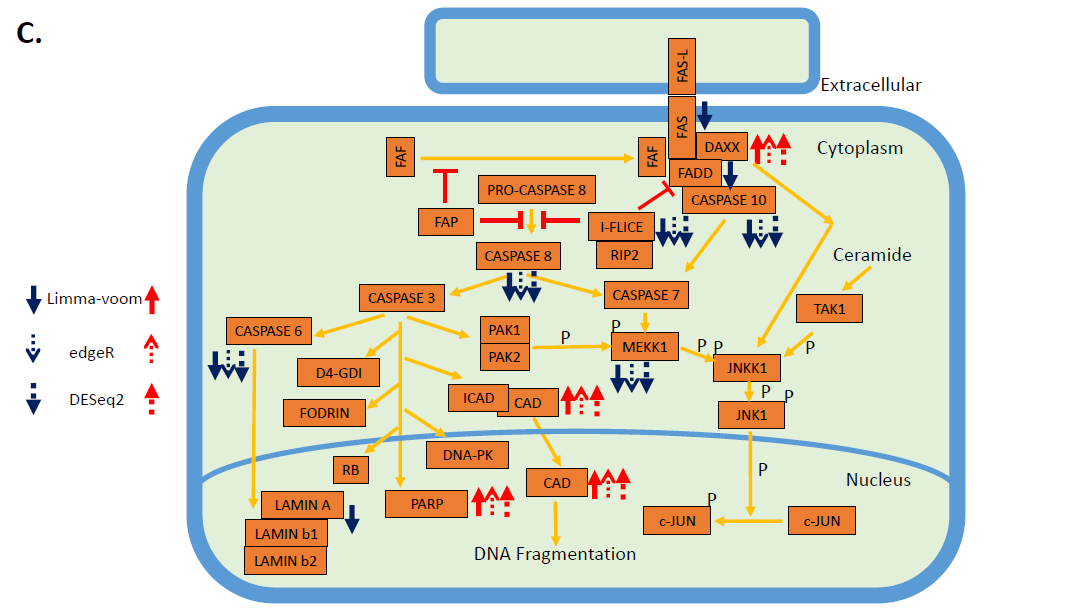


**Supplementary Figure 16.** Differential expressed genes showed coordinate and consensus down-regulated expression pattern for CASPASE genes in Ras-independent lung cell lines in a Biocarta pathway: Fas signaling pathway. **A.** Heatmap of the differential expressed genes (DEGs) from Fas signaling pathway between top 15 vs bottom 15 CCLE lung cell lines with highest vs lowest ssGSEA scores of the EMT signature. DEGs are from CCLE microarray data for lung cell lines, namely L_High_vs_L_Low_AdjP0_05 (detailed in Supplementary Table 1). Green arrows: Ras-dependent cell lines; Red arrows: Ras-independent cell lines.

**B.** Pathway overlay of DEGs from microarray data of lung cell lines of top 15 vs bottom 15 CCLE lung cell lines with highest vs lowest ssGSEA scores (**A**) on Fas signaling pathway. **C.** Pathway overlay of DEGs from RNAseq data of top 15 vs bottom 15 CCLE lung cell lines with highest vs lowest ssGSEA scores analyzed by three methods on Fas signaling pathway. High ssGSEA scores cell lines are more like Ras-dependent lines whereas low ssGSEA score cell lines are more like Ras-independent lines. Blue arrows showed down-regulated of corresponding genes in microarray or RNAseq data of cell lines with low ssGSEA scores (Ras independent samples) for different tumor origins, whereas red arrows for few genes up-regulated in lines with low ssGSEA scores. Gene alias as followed: MEKK1=MAP3K1;TAK1=MAP3K7;I-FLICE=CFLAR. Green arrows: Ras-dependent cell lines; Red arrows: Ras-independent cell lines.


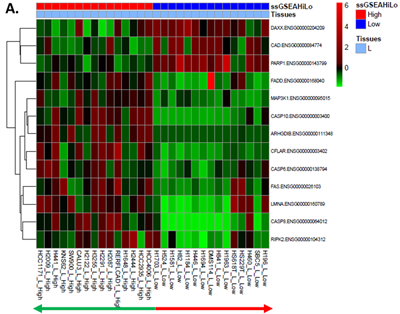


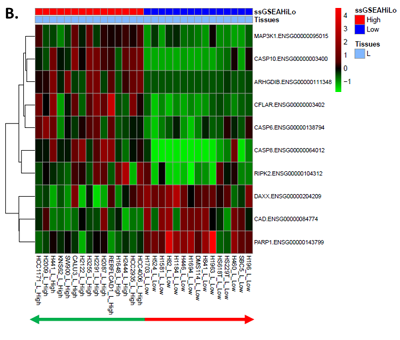


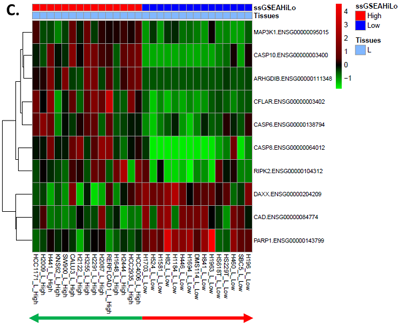


**Supplementary Figure 17**. Differential expressed genes from CCLE RNAseq data showed coordinate and consensus down-regulated expression pattern for CASPASE genes in Ras-independent CCLE lung cell lines in a Biocarta pathway: Fas signaling pathway regardless of the DEGs analysis methods. **A**. Heatmap of DEGs between top 15 vs bottom 15 CCLE lung cell lines with highest vs lowest ssGSEA scores from CCLE RNAseq data analyzed by limma-voom method. Gene list used is: L.High_vs_L.Low_limmavoom_adjP0_05 (Supplementary table 2). **B**. Heatmap of DEGs between top 15 vs bottom 15 CCLE lung cell lines with highest vs lowest ssGSEA scores from CCLE RNAseq data analyzed by edgeR method. Gene list used is: L.High_vs_L.Low_edgeR_FDR0_05 (Supplementary table 2). **C**. Heatmap of DEGs between top 15 vs bottom 15 CCLE lung cell lines with highest vs lowest ssGSEA scores from CCLE RNAseq data analyzed by DESeq2 method. Gene list used is: L.High_vs_L.Low_DESeq2_adjP0_05 (Supplementary table 2). Green arrows: Ras-dependent cell lines; Red arrows: Ras-independent cell lines.


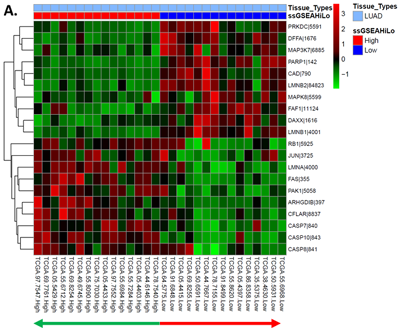


**
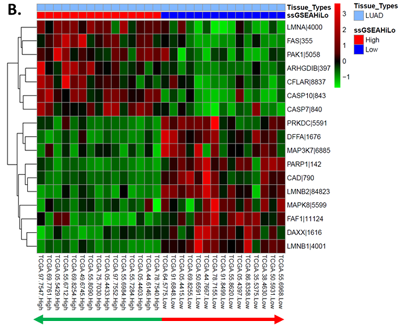
**

**
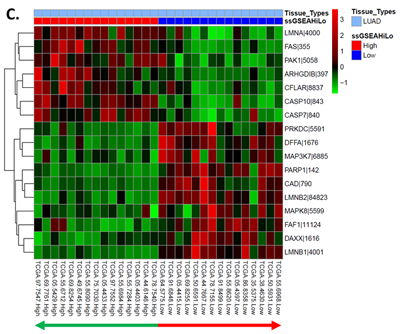
**


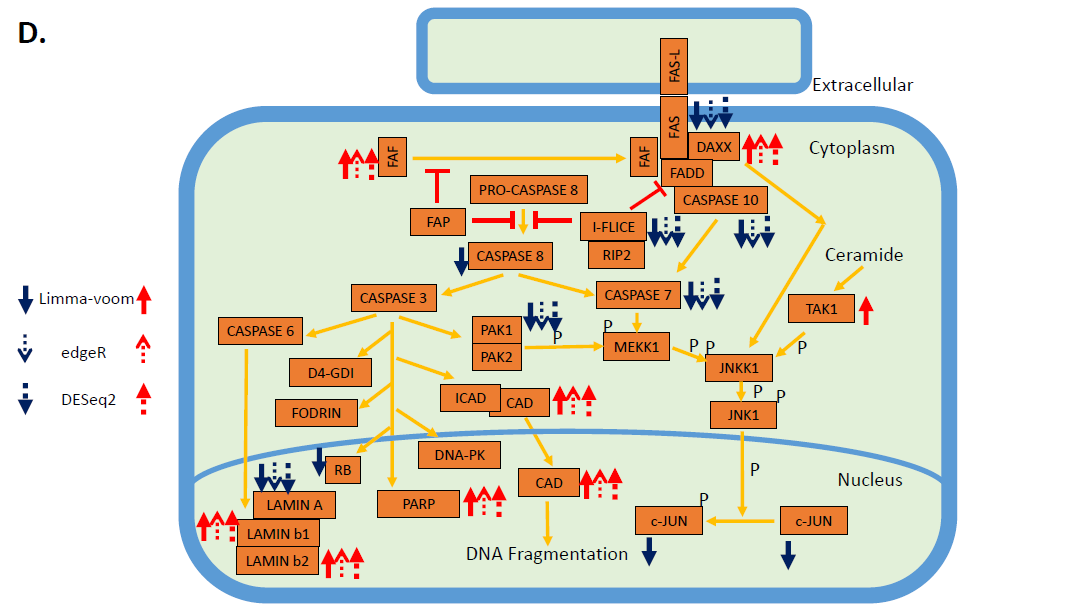


**Supplementary Figure 18**. Differential expressed genes from TCGA RNAseq data showed coordinate and consensus down-regulated expression pattern for Fas and downstream CASPASE genes in Ras-independent TCGA LUAD patient samples in a Biocarta pathway: Fas signaling pathway regardless of the DEGs analysis methods. **A.** Heatmap of DEGs between top 15 vs bottom 15 TCGA LUAD patient samples with highest vs lowest ssGSEA scores from TCGA RNAseq data analyzed by limma-voom method. **B.** Heatmap of DEGs between top 15 vs bottom 15 TCGA LUAD patient samples with highest vs lowest ssGSEA scores from TCGA RNAseq data analyzed by edgeR method. **C**. Heatmap of DEGs between top 15 vs bottom 15 TCGA LUAD patient samples with highest vs lowest ssGSEA scores from TCGA RNAseq data analyzed by DESeq2 method. **D**. Pathway overlay of DEGs from RNAseq data of top 15 vs bottom 15 TCGA LUAD patient samples with highest vs lowest ssGSEA scores analyzed by three methods (**A,B,C**) on Fas signaling pathway. DEGs of LUAD used are at threshold of FDR or adjusted p-value <=0.05. Blue arrows showed down-regulated of corresponding genes in RNAseq data of TCGA patient samples with low ssGSEA scores (Ras independent samples) for different RNAseq analysis methods, whereas red arrows for few genes up-regulated in samples with low ssGSEA scores. In **A., B.,C.,** Green arrows: Ras-dependent cell lines; Red arrows: Ras-independent cell lines.


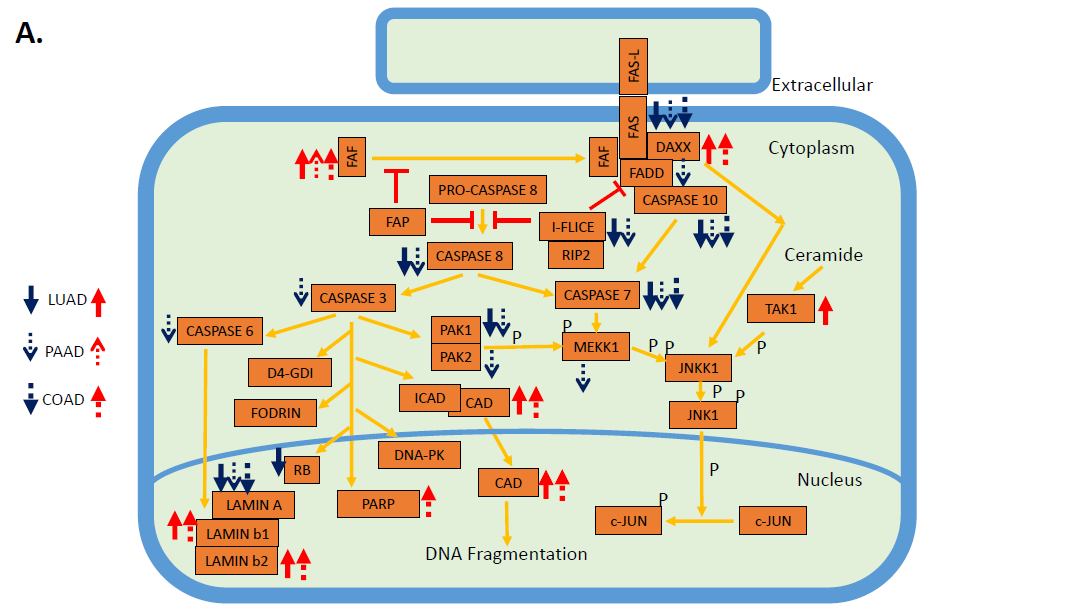


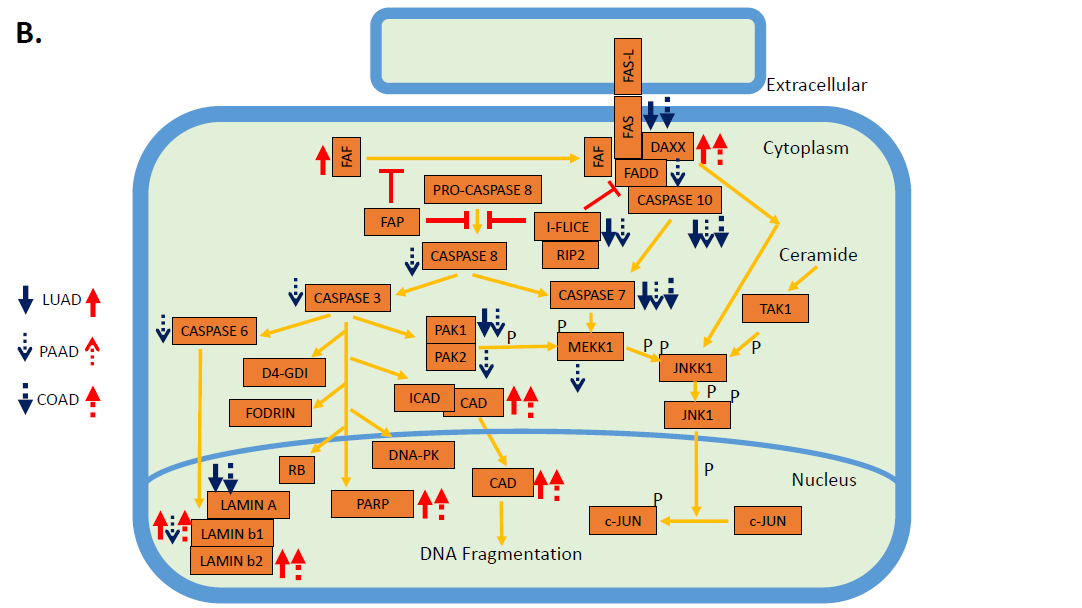


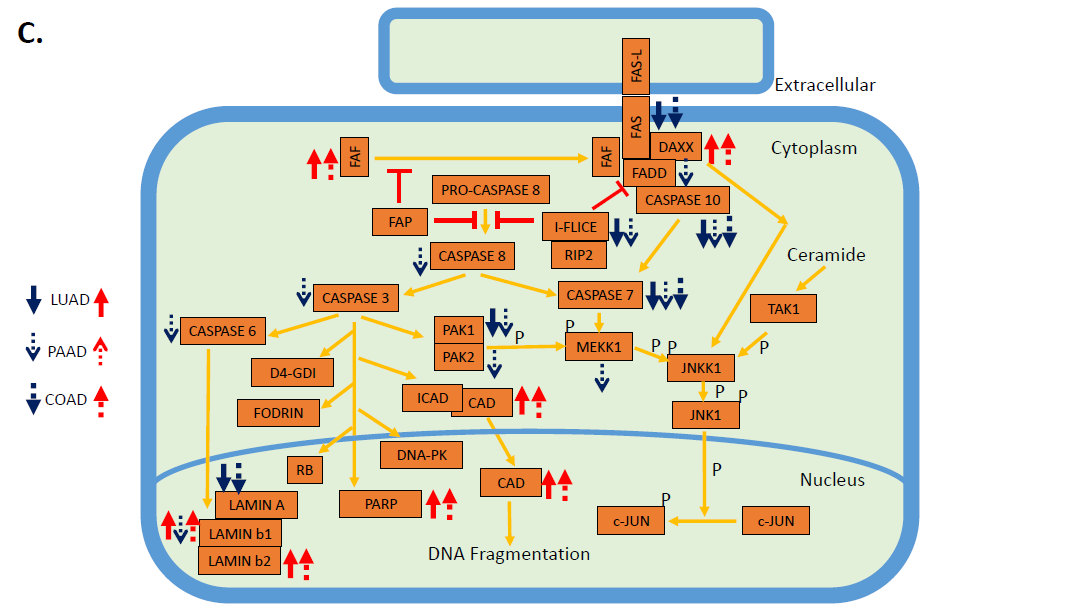


**Supplementary Figure 19**. Differential expressed genes from TCGA RNAseq data showed coordinate and consensus down-regulated expression pattern for Fas and downstream CASPASE genes in Ras-independent TCGA patient samples of three tumor types in a Biocarta pathway: Fas signaling pathway. **A.** Pathway overlay of DEGs from RNAseq data of top 15 vs bottom 15 TCGA LUAD patient samples with highest vs lowest ssGSEA scores from three types of TCGA tumors: LUAD, PAAD, and COAD on Fas signaling pathway. DEGs analyzed by limma-voom method. **B**. Pathway overlay of DEGs from RNAseq data of top 15 vs bottom 15 TCGA patient samples with highest vs lowest ssGSEA scores from three types of TCGA tumors: LUAD, PAAD, and COAD on Fas signaling pathway. DEGs analyzed by edgeR method. **C.** Pathway overlay of DEGs from RNAseq data of top 15 vs bottom 15 TCGA patient samples with highest vs lowest ssGSEA scores from three types of TCGA tumors: LUAD, PAAD, and COAD on Fas signaling pathway. DEGs analyzed by DESeq2 method. All DEGs used are at threshold of FDR or adjusted p-value <=0.05. Blue arrows showed down-regulated of corresponding genes in RNAseq data of TCGA patient samples with low ssGSEA scores (Ras independent samples) for patients of different tumor types, whereas red arrows for few genes up-regulated in samples with low ssGSEA scores.


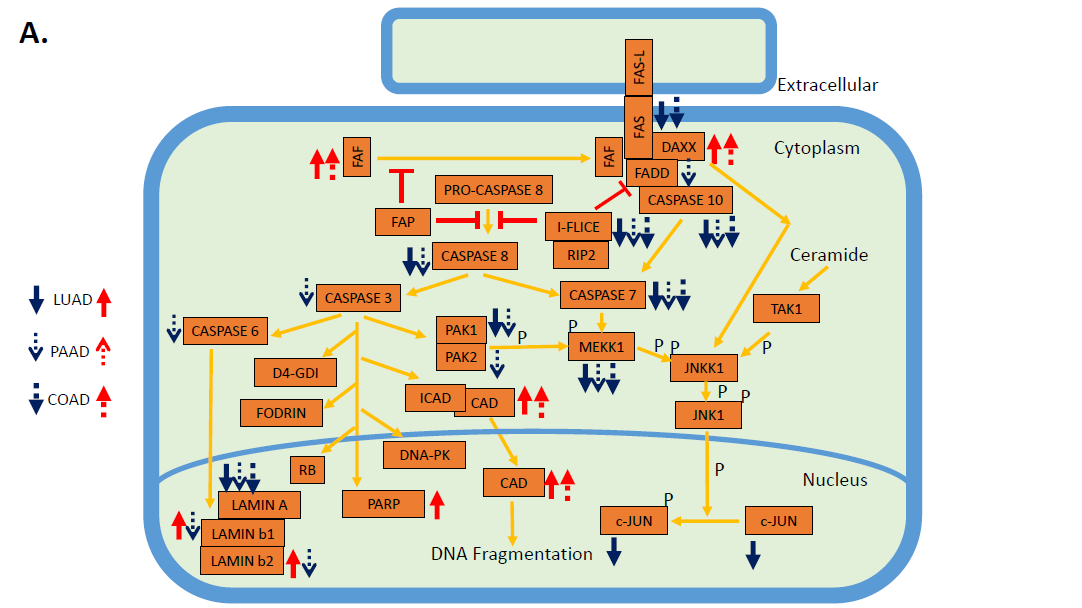


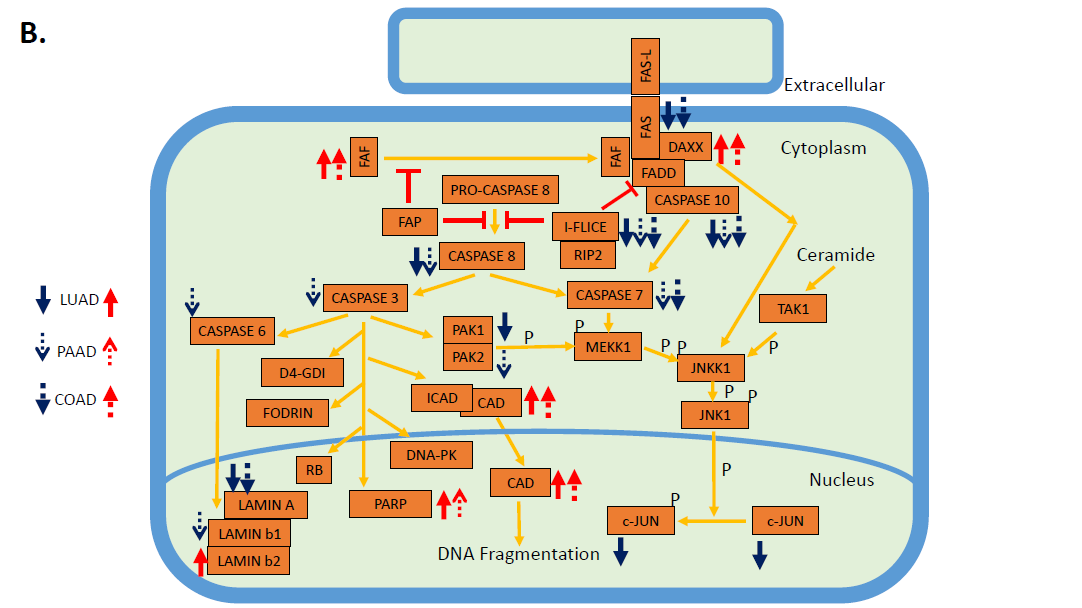


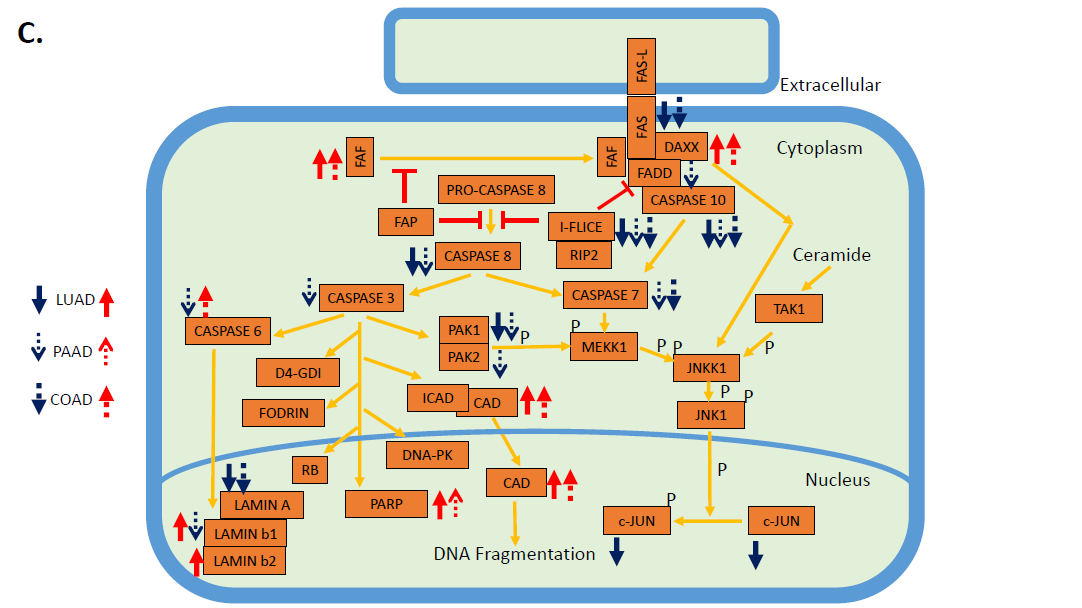


**Supplementary Figure 20.** Differential expressed genes analysis similar to **Supplementary Figure 19** except using top 30 vs bottom 30 TCGA patient samples to test robustness of results . **A.** Pathway overlay of DEGs from RNAseq data of top 30 vs bottom 30 TCGA patient samples with highest vs lowest ssGSEA scores from three types of TCGA tumors: LUAD, PAAD, and COAD on Fas signaling pathway. DEGs analyzed by limma-voom method. **B.** Pathway overlay of DEGs from RNAseq data of top 30 vs bottom 30 TCGA patient samples with highest vs lowest ssGSEA scores from three types of TCGA tumors: LUAD, PAAD, and COAD on Fas signaling pathway. DEGs analyzed by edgeR method. **C.** Pathway overlay of DEGs from RNAseq data of top 30 vs bottom 30 TCGA patient samples with highest vs lowest ssGSEA scores from three types of TCGA tumors: LUAD, PAAD, and COAD on Fas signaling pathway. DEGs analyzed by DESeq2 method. DEGs used are at threshold of FDR or adjusted p-value <=0.05. Blue arrows showed down-regulated of corresponding genes in RNAseq data of TCGA patient samples with low ssGSEA scores (Ras independent samples) for patients of different tumor types. Red arrows for few genes up-regulated in samples with low ssGSEA scores.


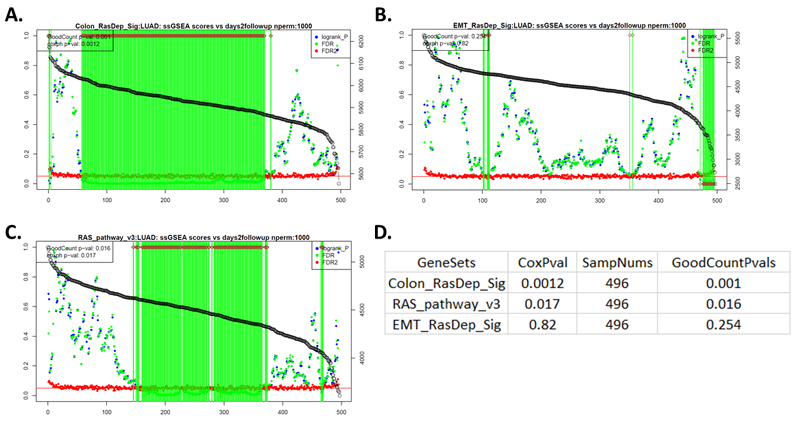


**Supplementary Figure 21.** GradientScanSurv results in TCGA LUAD data (lung adenocarcinoma) using ssGSEA scores of RDI gene signatures and RAS pathway. Survival-gradient plots derived from GradientScanSurv method (see Methods section) with gradients of selected gene signatures: **A.** Colon_RasDep_Sig: Colon Signature ; **B.** EMT_RasDep_Sig: EMT signatures; **C.** RAS_pathway_v3 (Ras pathway genes annotated from Ras central). **D.** Statistical summary in the table, where GoodCountPvals are derived from GradientScanSurv method showing Colon and Ras pathway signatures’ ssGSEA scores but not EMT signature have significant association with patients’ survival outcome. SampNums: numbers of samples in the dataset; CoxPval: Cox regression model derived p-values.


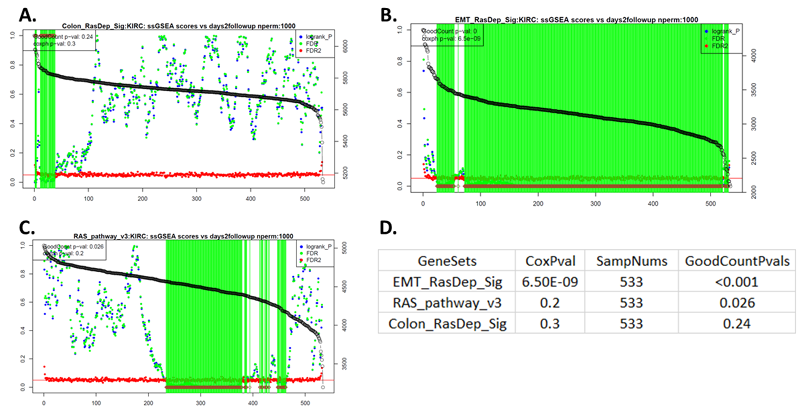


**Supplementary Figure 22.** GradientScanSurv results in TCGA KIRC data (renal clear cell carcinoma) using ssGSEA scores of RDI gene signatures and RAS pathway. Survival-gradient plots derived from GradientScanSurv method (see Methods section) with gradients of selected gene signatures: **A.** Colon_RasDep_Sig: Colon Signature; **B.** EMT_RasDep_Sig: EMT signatures; **C.** RAS_pathway_v3 (Ras pathway genes annotated from Ras central). **D.** Statistical summary in the table, where GoodCountPvals are derived from GradientScanSurv method showing EMT and Ras pathway signatures’ ssGSEA scores but not Colon signature have significant association with patients’ survival outcome. SampNums: numbers of samples in the dataset; CoxPval: Cox regression model derived p-values.


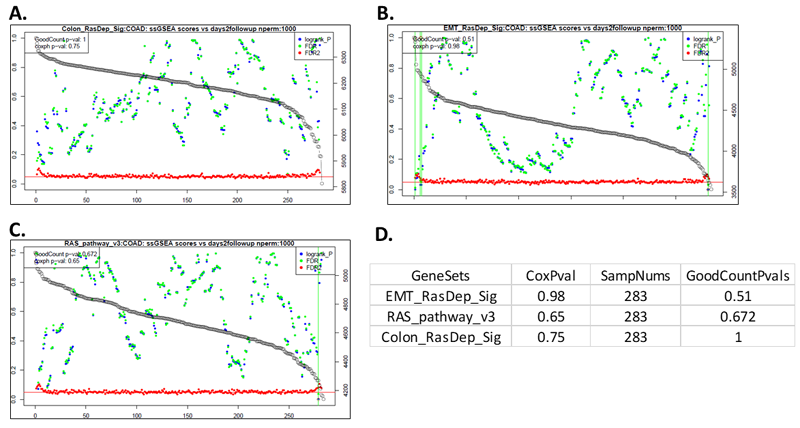


**Supplementary Figure 23.** GradientScanSurv results in TCGA COAD data (colon adenocarcinoma) using ssGSEA scores of RDI gene signatures and RAS pathway. Survival-gradient plots derived from GradientScanSurv method (see Methods section) with gradients of selected gene signatures: **A.** Colon_RasDep_Sig: Colon Signature; **B.** EMT_RasDep_Sig: EMT signatures; **C.** RAS_pathway_v3 (Ras pathway genes annotated from Ras central). **D.** Statistical summary in the table, where GoodCountPvals are derived from GradientScanSurv method showing all signatures’ ssGSEA scores have no significant association with patients’ survival outcome. SampNums: Numbers of samples in the dataset; CoxPval: Cox regression model derived p-values.
